# Antifungal Effects of Volatile Organic Compounds Produced by *Rahnella aquatilis* JZ-GX1 Against *Colletotrichum gloeosporioides* in *Liriodendron chinense* × *tulipifera*

**DOI:** 10.3389/fmicb.2020.01114

**Published:** 2020-05-28

**Authors:** Wei-Liang Kong, Lin Rui, Hang Ni, Xiao-Qin Wu

**Affiliations:** ^1^Co-Innovation Center for Sustainable Forestry in Southern China, College of Forestry, Nanjing Forestry University, Nanjing, China; ^2^Jiangsu Key Laboratory for Prevention and Management of Invasive Species, Nanjing Forestry University, Nanjing, China

**Keywords:** *Rahnella aquatilis*, VOCs, *Colletotrichum gloeosporioides*, mycelial growth, spore germination

## Abstract

The use of volatile organic compounds (VOCs) produced by microorganisms for the biological control of plant diseases has attracted much attention in recent years. In this study, the antifungal activity and identity of VOCs produced by *Rahnella aquatilis* JZ-GX1 isolated from the rhizosphere soil of pine were determined and analyzed. The effect of the VOCs on the mycelial growth of *Colletotrichum gloeosporioides*, the pathogen of *Liriodendron chinense* × *tulipifera* black spot, was determined by a joined-petri dish fumigation method. An *in vitro* leaf inoculation method was used to determine the fumigation effect of the VOCs on *Liriodendron* black spot. VOCs with antifungal activity were collected by headspace solid-phase microextraction (SPME), and their components were analyzed by gas chromatography-mass spectrometry (GC-MS). The results showed that the VOCs secreted by JZ-GX1 inhibited the mycelial growth of the tested pathogen. The VOCs destroyed the morphology of the mycelium, significantly increased the permeability of the cell membrane and downregulated the expression of pathogenicity-related genes during mycelial infection, thus inhibiting the expansion of anthracnose disease spots in leaves. In the volatile compound profile, 3-methyl-1-butanol and 2-phenylethyl methyl ether significantly inhibited the mycelial growth and spore germination of *C. gloeosporioides*. This work provides a new strategy for the research and application of microorganisms and bioactive compounds to control plant anthracnose.

## Introduction

Biological control of plant diseases is an important measure to reduce the use of chemical pesticides and improve plant health (Eva et al., 2019). Research based on the interaction between antagonistic microorganisms and pathogens has always been the focus of biocontrol research. Studies have shown that biocontrol agents such as *Bacillus*, *Pseudomonas*, *Burkholderia*, and *Streptomyces* play important roles in plant pathogen inhibition (Jiang et al., 2018; Roxane et al., 2018; Randa et al., 2019; Jeon et al., 2019). Biocontrol mechanisms mainly include the production of antibiotics, occupation of active sites, and nutrient or mineral competition, of which the most common strategy is the secretion of antifungal metabolites (Huang et al., 2017). At present, the most well-studied antifungal metabolites are antibiotics, cell wall-degrading enzymes and volatile organic compounds (VOCs), and the VOCs produced during microbial metabolism are particularly important biocontrol factors (Tagele et al., 2019).

In recent years, attention to VOC research has increased. Compared with other secondary metabolites of microorganisms, VOCs have many desirable properties, such as low molecular weight, low polarity, high vapor pressure, low boiling point, and lipophilicity (Sharifi and Ryu, 2018). VOCs at low concentrations can be sensed and can be transmitted over long distances, mediating indirect interactions between organisms; thus, VOCs have been used for the biological control of plant diseases (Sharifah et al., 2019). Wan et al. (2008) detected phenylethyl alcohol in the volatile profile of *Streptomyces platensis* F-1, and the application of phenylethyl alcohol slowed the growth of *Botrytis cinerea* and maintained the aroma in strawberry. The VOCs produced by *Pseudomonas aureofaciens* SPS-41 can be used as a biological fumigant to control disease in sweet potato tuber roots (Zhang et al., 2019). The volatiles produced by *Enterobacter asburiae* Vt-7 inhibited aflatoxin production in peanut during storage (Gong et al., 2019).

*Colletotrichum gloeosporioides* is widely distributed in tropical and subtropical regions and can infect mango, peaches, *Liriodendron chinense* × *tulipifera, Cunninghamia lanceolata*, and *Camellia sinensis*, causing anthracnose in leaves and fruits (Huang et al., 2018; Tang et al., 2019; Wu et al., 2019; Zhu et al., 2019; Shang et al., 2020). The conidia of this pathogen can infect the tender leaves, twigs, flowers and fruits of the host, causing leaves, flowers and fruits to fall, and fruit to decay during storage, resulting in serious economic losses. At present, chemical application is the main control strategy for plant anthracnose. However, with the widespread application of chemical pesticides, the selection pressure on pathogens increases. Anthracnose in many areas also has varying degrees of drug resistance. Wang et al. (2019) found significant differences in the sensitivity of 13 *Colletotrichum anthracnose* isolates from walnut fruits and leaves on walnut plantations in China to the same fungicide. Mancozeb, which is used to control anthracnose in mango orchards, has had similar effects (Chanchaichaovivat et al., 2007). Therefore, reducing the drug resistance of pathogens through biological control has become an important issue in the prevention and control of agricultural and forestry diseases.

*Rahnella aquatilis* JZ-GX1 is a plant growth-promoting bacterium isolated from the rhizosphere of *Pinus massoniana* in our laboratory. Previous studies have shown that it has strong inhibitory activity against poplar canker pathogen (*Cytospora chrysosperma*) and seedling quenching pathogen (*Rhizoctonia solani*) (Song et al., 2017; Kong et al., 2019b) and can promote the growth of *Cinnamomum camphora* and *Pinus massoniana* (Li et al., 2013; Kong et al., 2019a). However, it is not clear whether the strain can produce volatile compounds and whether its VOCs have inhibitory effects on plant pathogens. In this study, the antagonistic effect of the VOCs emitted by the JZ-GX1 strain against the anthracnose pathogen *Liriodendron chinense* × *tulipifera* was evaluated to isolate and identify individual volatile compounds with antifungal activity, to reveal the antagonistic mechanism behind the effect of the volatile compounds against plant pathogens, and to develop new microbial resources to control plant diseases caused by *C. gloeosporioides*.

## Materials and Methods

### Tested Bacterial and Fungal Strains

*Rahnella aquatilis* JZ-GX1 is a plant growth-promoting bacterium isolated from the rhizosphere soil of a 28-year-old *P. massoniana* in Nanning, Guangxi. It is now stored in the typical Culture Preservation Center of China (CCTCC, No: M2012439). After activation, the JZ-GX1 strain was cultured overnight on LB liquid medium at 28°C.

The pathogen *C. gloeosporioides* was isolated from a susceptible *Liriodendron chinense* × *tulipifera* on the campus of Nanjing Forestry University. The strain was cultured on potato glucose agar (PDA) medium at 25°C for 7 days.

### Determination of the Antagonistic Effect of VOCs Produced by Strain JZ-GX1 on *C. gloeosporioides*

The antifungal activity of VOCs produced by JZ-GX1 was detected after culturing on two sealed petri dishes (Gao et al., 2017). One petri dish contained 20 mL LB medium, and the other 20 mL PDA medium. The LB medium was coated with 100 μL JZ-GX1 suspension, and a 6 mm diameter plug of *C. gloeosporioides* was placed on the PDA plate. Then, the bottoms of the two petri dishes were sealed with Parafilm and cultured in a constant-temperature incubator at 25°C for 5 days. Each experiment was repeated three times. The inhibition rate = (Cd - Td) × 100%/Cd, where Cd is the colony diameter on the control PDA plate and Td is the colony diameter on the treated PDA plate.

### Scanning Electron Microscopy (SEM) Observation of the Mycelium of *C. gloeosporioides*

The mycelium morphology of *C. gloeosporioides* was observed and analyzed by SEM (Quanta 200FEIJI, United States) in *C. gloeosporioides* after 5 days of VOC treatment and in *C. gloeosporioides* that had not been exposed to the JZ-GX1 strain. A mycelium sample was fixed in 4% glutaraldehyde solution for 24 h and washed with pH = 7.2 phosphate buffer (Edgar et al., 2019). The mycelium samples were dehydrated with an ethanol gradient (70, 80, 90%, and anhydrous ethanol). Then, the gold layer was sputtered with liquid CO_2_ in a critical point dryer (EMITECH K850) for 15 min. The specimens were subsequently mounted on stubs and sputtered with gold (HITACH E-1010). The scanning voltage was 20 kV (Li et al., 2013).

### Determination of Nucleic Acid Exosmosis by Pathogenic Mycelium

To evaluate the effect of VOCs on the nucleic acid leakage of *C. gloeosporioides*, the OD260 of a mycelial cell suspension was determined by ultraviolet spectrophotometry. LB medium was added to one petri dish, onto which 100 μL JZ-GX1 bacterial suspension was coated, and PDA medium was poured into another petri dish. After cooling, sterilized cellophane was spread on the PDA medium, and 100 μL of a spore suspension (10^8^ cfu/mL) of *C. gloeosporioides* was evenly coated on the cellophane. Then, the bottoms of the two dishes were sealed with Parafilm. The plates were cultured at 25°C, and mycelial cells were removed at 12, 24, 36, 48, and 60 h and washed with 5 mL aseptic water. After centrifugation, the OD260 value of the supernatant was determined by an ultraviolet spectrophotometer (Zhang, 2015). The experiment was carried out three times in parallel and repeated three times.

### Determination of Malondialdehyde (MDA) and Soluble Sugar in Pathogenic Mycelium

A total of 0.5 g mycelium of *C. gloeosporioides* fumigated with VOCs, 0.1 g quartz sand and 2 mL 10% trichloroacetic acid were fully ground into a homogenate with a mortar and pestle. Then, 8 mL 10% trichloroacetic acid was added, and grinding continued. The homogenate was centrifuged at 4000 rpm for 10 min, and MDA was recovered in the supernatant. Next, 2 mL supernatant was added to a clean test tube, and a control tube was filled with 2 mL distilled water; 2 mL 0.6% thiobarbituric acid solution was added to each tube. The tubes were shaken and allowed to react in a boiling water bath for 15 min. The tubes were quickly cooled and then centrifuged. The absorbance (A) of the final supernatant was determined at 532, 600, and 450 nm, and the MDA concentration was calculated as follows: c (MDA) (μM) = 6.45 (A532 - A600) - 0.56A450; c (soluble sugar) (μM) = 11.71A450 (Song et al., 2017).

### RNA Extraction and RT-qPCR Analysis of Pathogenic Mycelium

The mycelium of *C. gloeosporioides* was inoculated in CMC liquid medium and shaken at 25°C and 150 rpm for 48 h. The suspension was filtered through monolayer gauze and diluted to obtain a 10^6^ cfu/mL fresh spore suspension. Then, 100 μL conidial suspension was coated on a PDA plate. The LB plate inoculated with JZ-GX1 was joined with the PDA plate containing the conidial suspension of *C. gloeosporioides*. The bottoms of the two culture dishes were sealed with Parafilm and cultured in the dark at 28°C. Conidia on PDA without JZ-GX1 treatment were used as a control. *C. gloeosporioides* was collected 24 h later for RNA extraction. Total RNA was extracted with TRIzol reagent according to the manufacturer’s instructions. After DNaseI treatment, 2 μg ribonucleic acid was added to the 20 μL reaction system, and first-strand cDNA was synthesized by reverse transcription according to the manufacturer’s instructions. HiScript II Q Select RT Supermix was used to prepare cDNA samples for qPCR (China). Using 1.0 μL cDNA diluted 1:10 as the template, the whole reaction system was carried out in an ABI 7500 system (Applied Biosystems, United States). Three pathogenicity-related genes, polyketone synthase (*PKS*), endotoxin galacturonase (*PG*), and cysteine dehydratase (*SCD*), were selected for RT-qPCR analysis (Table 1). The actin gene was used as an internal reference gene because of its relatively stable expression level. The relative quantification of gene expression changes was carried out by the 2^–Δ^ ^Δ^ ^CT^ method (Li Z.Q. et al., 2017; Su et al., 2018).

**TABLE 1:** Primers used in RT-qPCR analysis.

**Gene name**	**Gene functions**	**Primers**
PKS	Polyketone synthesis	TGCTCATGATGGAGACGGAAG
		GCGGGTGATGAAGTTACGGAT
PG	Degraded cell wall	ATCAAGACCATCGCTAAGAAGACC
		TCCTGCTGGATCACGATGC
SCD	Melanin synthesis	CACCCAAGTTCGCCATATCC
		CGAGAAGAACGATGTCAAGGTTG
ACT	Endogenous control, Reference gene	AGCGGAAAGCCTCGCAGT
		TGTCGTTACCATCTCGACCCA

### Determination of Anthracnose Spot on *Liriodendron chinense* × *tulipifera* Treated With VOCs

A petri dish with a diameter of 15 cm was coated with JZ-GX1 bacterial solution, and a blank LB plate was used as the control. Medium-sized hybrid *Liriodendron* leaves were placed at the bottom of another petri dish, and the petioles were wrapped with wet cotton balls for moisturization. Then, the leaf surfaces were sprayed and disinfected with 75% alcohol, and two inoculation points were selected on each side of the main vein. After puncture of the leaf surface, the wound was inoculated with *C. gloeosporioides* plugs (Φ = 6 mm), with 10 dishes per treatment and three replicates. Each petri dish inoculated with JZ-GX1 and each petri dish inoculated with pathogenic fungal leaves were joined together and sealed with Parafilm. After 2 and 4 days of confrontation culture at 25°C, the expansion of lesions on the leaves was observed, and the diameter of each lesion was measured (Xu et al., 2017; Wang et al., 2018).

### Identification of VOCs and Inhibitory Analysis

The analysis of VOCs emitted by JZ-GX1 was performed with headspace solid-phase microextraction (HS-SPME) coupled with gas chromatography-mass spectrometry (GC-MS). A single colony of JZ-GX1 was inserted into a 200 mL flask containing 50 mL of liquid LB medium and fermented at 28°C with shaking at 180 rpm for 2 days. LB liquid medium without bacterial inoculation was used as the control. A 65 μm PDMS/DVB fiber was selected for the determination of VOCs. The SPME fiber was inserted into the flask and cultured at 40°C for 30 min. After the extraction, the fiber was quickly retracted, and the needle was pulled out and immediately inserted into the GC inlet (TraceISQ, Thermo Fisher Scientific, United States). The fiber was pushed down into the inlet at 230°C desorption for 3 min. The CK and JZ-GX1 treatments were tested three times.

Gas chromatographic conditions: DB-5MS capillary column (30 m × 0.25 mm × 0.25 μm), He carrier gas; injector port temperature of 230°C; initial temperature of 40°C, maintained for 3 min, raised to 95°C at 10°C/min, then to 230°C at 3°C/min, and maintained for 5 min.

Mass spectrometry conditions: EI ion source; electron energy of 70 eV; ion source temperature of 250°C; interface temperature of 250°C; data acquisition rate, 0.2 s/time and spectrum retrieval using the NIST 05 and NIST 05s libraries (Zhang, 2013).

To test the antifungal activity of JZ-GX1 VOCs, we used authentic reference standard compounds purchased from Rongshide Trading Co., Ltd. (Nanjing, China). PDA medium (20 ml) was added to a petri dish; 7-day-old anthracnose plugs (6 mm) and conidia (100 μL of conidia at 10^8^ cfu/mL) were inoculated in the center of the PDA medium with a punch. Another petri dish was added with authentic standard to final concentrations of 10, 20, 100, and 200 μL/L (compound volume to airspace volume). The bottoms of the two petri dishes were joined together and sealed with film. After culturing at 25°C for 3 days, the inhibition by the standard was observed; anthrax was inoculated alone as the control. Each process was repeated three times.

### Statistical Analysis

The data were analyzed by analysis of variance and Duncan’s multiple comparison with SPSS 22.0 software, and the standard errors of all mean values were calculated (*P <* 0.05).

## Results

### Antagonistic Effect of VOCs Produced by JZ-GX1 Against *C. gloeosporioides*

**FIGURE 1:**
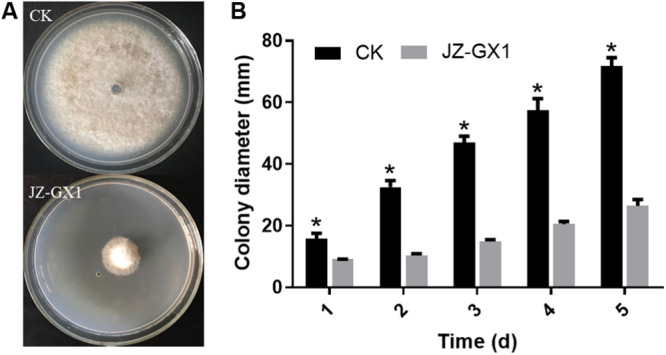
Effect of *R. aquatilis* JZ-GX1 on the mycelial growth of *C. gloeosporioides*. **(A)** Colony morphology of *C. gloeosporioides in vitro*. **(B)** Colony diameter of *C. gloeosporioides*. Vertical bars represent the standard deviation of the average. One-way ANOVA analysis was performed and Duncan’s *post hoc* test was applied. Asterisks indicate statistically significant differences (*p* < 0.05) among treatments.

The determination of the mycelial growth inhibition rate showed that the VOCs produced by JZ-GX1 had a good inhibitory effect on the colony growth of *C. gloeosporioides*. Compared with that of the control group, the colony diameter of *C. gloeosporioides* treated with JZ-GX1 was significantly inhibited (Figure 1A). With extension of the culture time, the relative rate of inhibition by the VOCs produced by JZ-GX1 to *C. gloeosporioides* gradually increased (Figure 1B), reaching 63.16% after 5 days of culture.

### Morphological Changes of Mycelium After Coculture of *C. gloeosporioides* and Strain JZ-GX1

**FIGURE 2:**
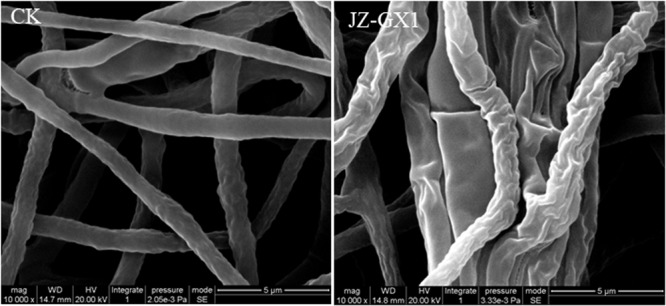
Morphological observation of *C. gloeosporioides* via SEM. CK: untreated control group; JZ-GX1: treated with VOCs produced by *R. aquatilis* JZ-GX1.

To observe the effect of the VOCs produced by JZ-GX1 on the mycelium morphology of *C. gloeosporioides*, changes in mycelium morphology were observed by SEM. The results showed that the surface of the pathogenic mycelium treated with JZ-GX1 VOCs was rough, with obvious wrinkles and collapsed areas, while the surface of the mycelium without JZ-GX1 VOCs treated was neat, plump and smooth (Figure 2).

### Effect of VOCs Released by JZ-GX1 on the Cell Membrane Permeability of *C. gloeosporioides*

**FIGURE 3:**
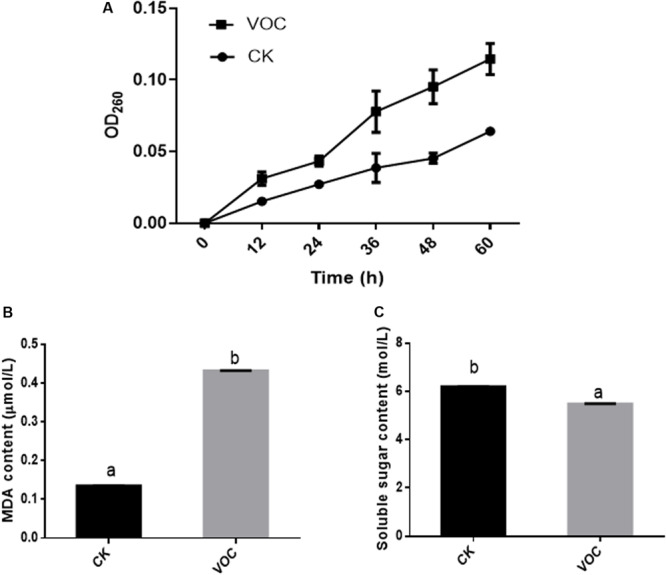
Effects of *R. aquatilis* JZ-GX1 on nucleic acid leakage **(A)**, the MDA content **(B)**, and the soluble sugar content **(C)** in the mycelia of *C. gloeosporioides*. The vertical bars represent the standard deviation of the average. One-way ANOVA analysis was performed and Duncan’s *post hoc* test was applied. Different letters indicate statistically significant differences (*p* < 0.05) among treatments.

After the VOCs produced by JZ-GX1 were incubated with *C. gloeosporioides* for 36 h, the OD260 of the centrifuged mycelial suspension was significantly higher than that of the blank control group (Figure 3A). The longer the incubation time with the VOCs produced by JZ-GX1 was, the higher the OD260 value of the centrifuged mycelial suspension, indicating that the cell membrane of *C. gloeosporioides* was greatly damaged, that is, high leakage of nucleic acids, by the VOCs.

The damage to *C. gloeosporioides* lipids by JZ-GX1 VOCs was determined by measuring the MDA content. Compared with that in the control, the content of MDA in the mycelium of *C. gloeosporioides* increased after treatment with JZ-GX1 VOCs for 60 h (Figure 3B), indicating that the VOCs produced by JZ-GX1 enhanced the oxidative damage in the mycelium of *C. gloeosporioides*. In addition, the soluble sugar concentration in *C. gloeosporioides* in the treatment group was significantly lower than that in the control group (Figure 3C), which also reflected the hydrolysis of the mycelial cell wall of *C. gloeosporioides*.

### *R. aquatilis* JZ-GX1 Inhibited the Expression of Pathogenicity-Related Genes in *C. gloeosporioides*

**FIGURE 4:**
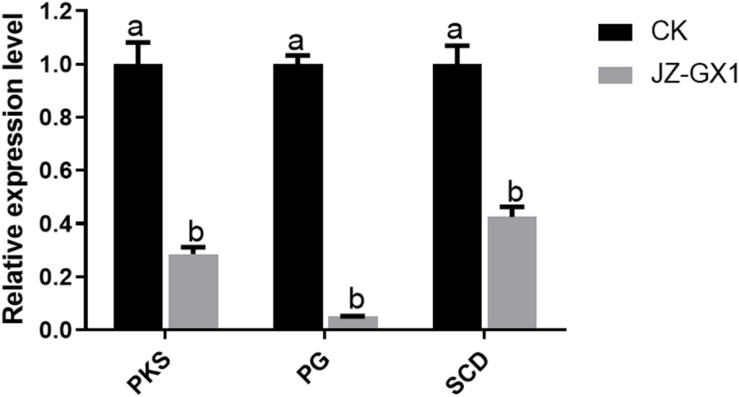
Relative expression of pathogenicity-related genes in *C. gloeosporioides* treated with *R. aquatilis* JZ-GX1 VOCs. The vertical bars represent the standard deviation of the average. One-way ANOVA analysis was performed and Duncan’s *post hoc* test was applied. Different letters indicate statistically significant differences (*p* < 0.05) among treatments.

To better understand the effect of JZ-GX1 VOCs on the infection of *Liriodendron chinense* × *tulipifera* leaves by *C. gloeosporioides*, the differential expression of pathogenicity-related genes in *C. gloeosporioides* was analyzed. Compared with the expression in the control, the selected genes were downregulated in the presence of JZ-GX1 VOCs. In particular, the expression of the *PG* gene was downregulated 19.61-fold. The expression of *PKS* and *SCD* decreased 3.50- and 2.35-fold, respectively, which was significantly different from their expression in the control (Figure 4).

### Inhibition by the VOCs Produced by *R. aquatilis* JZ-GX1 of Infection by *C. gloeosporioides*

**FIGURE 5:**
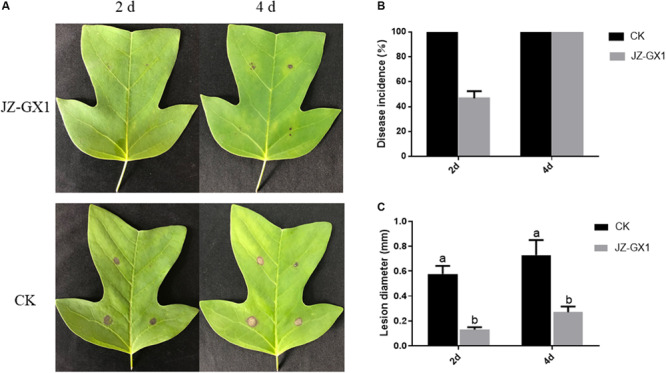
Inhibition by JZ-GX1 of spot expansion on *Liriodendron chinense* × *tulipifera* leaves. **(A)** Symptoms of anthracnose in leaves, **(B)** incidence of anthracnose in leaves, and **(C)** spot diameter of anthracnose. One-way ANOVA analysis was performed and Duncan’s *post hoc* test was applied. The vertical bars represent the standard deviation of the mean. Different letters indicate statistically significant differences (*p* < 0.05) among treatments.

The VOCs produced by JZ-GX1 significantly inhibited leaf anthracnose on *Liriodendron chinense* × *tulipifera* caused by *C. gloeosporioides* (Figure 5A). Two days after inoculation with *C. gloeosporioides*, the control leaves of *Liriodendron chinense* × *tulipifera* were completely infected, while the incidence in leaves treated with the JZ-GX1 strain was 45.7% (Figure 5B). After 2 and 4 days of inoculation, the average spot diameters of leaves treated with the JZ-GX1 strain were 5.67 and 15 mm, respectively, while those of control leaves were 28.33 and 66 mm, respectively (Figure 5C). These results showed that VOCs from the JZ-GX1 strain could significantly inhibit the expansion of anthracnose spots in *Liriodendron chinense* × *tulipifera* leaves.

### Collection and Identification of VOCs From *R. aquatilis* JZ-GX1

**FIGURE 6:**
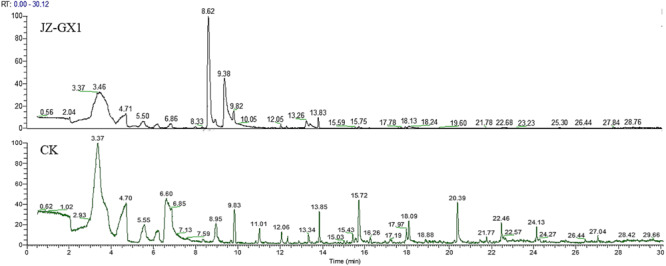
GC-MS/MS spectra of the VOCs emitted from JZ-GX1 incubated in LB medium for 48 h (JZ-GX1) and non-inoculated LB medium (CK).

The VOCs from JZ-GX1 were collected by an SPME syringe and analyzed by a GC-MS/MS system. VOCs that were also detected in the LB medium and VOCs with relative contents less than 0.5% were filtered out. As shown in Figure 6, there is a very obvious difference between the control and strain JZ-GX1 (Supplementary Data Sheets S1, S2). Eight VOCs released by JZ-GX1 were identified: two acids, two alcohols, two ketones, one ester, and one ether. The most abundant VOCs were 2-Phenylethyl methyl ether (22.87 ± 12.34% by GC), followed by phenylethyl alcohol (16.67 ± 6.61%). To evaluate their potential biological activity, we purchased the reference materials in Table 2 and determined their antagonistic activities against mycelium and conidia of *C. gloeosporioides*.

**TABLE 2:** GC-MS/MS VOC profile of strain JZ-GX1.

**Retention time (min)**	**Relative peak area (%)**	**CAS#**	**Compound**
4.11 ± 0.00	0.78 ± 0.65	123-51-3	1-Butanol, 3-methyl-
5.05 ± 0.01	0.51 ± 0.16	123-92-2	1-Butanol, 3- methyl-, acetate
6.21 ± 0.01	1.52 ± 0.39	6068-76-4	3,2′-Dihydroxyflavone
8.62 ± 0.01	22.87 ± 12.34	3558-60-9	2-Phenylethyl methyl ether
8.95 ± 0.01	1.52 ± 0.44	2078-13-9	4-Hydroxybenzoic acid
9.38 ± 0.01	16.67 ± 6.61	60-12-8	Phenylethyl Alcohol
12.05 ± 0.00	0.55 ± 0.33	53044-27-2	Phosphonoacetic Acid, 3TMS derivative
13.27 ± 0.02	1.23 ± 0.53	112-12-9	2-Undecanone

*Data are presented as means of three replicates ± standard deviation (SD).*

### Determination of Antifungal VOCs and Analysis of the Minimum Inhibitory Concentration (MIC) of JZ-GX1 Against *C. gloeosporioides*

**FIGURE 7:**
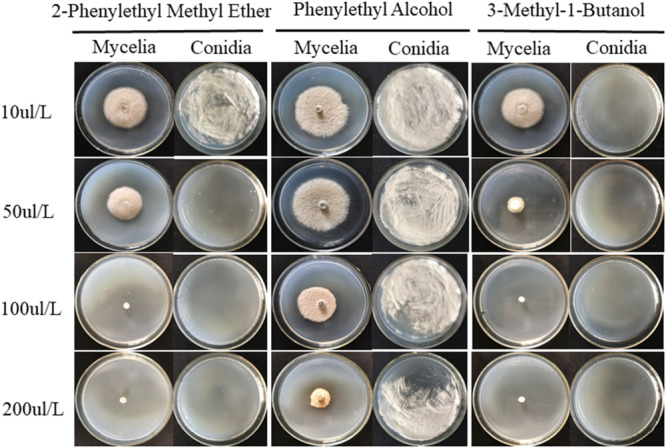
Three individual compounds, 2-phenylethyl methyl ether, phenylethyl alcohol and 3-methyl-1-butanol, at different concentrations were assayed for their inhibitory activity against the mycelial growth and conidial germination of *C. gloeosporioides*.

Standards for 8 VOCs released by JZ-GX1 were used to determine the antifungal activity against *C. gloeosporioides.* None of the standards except phenylethyl alcohol, 3-methyl-1-butanol and 2-phenylethyl methyl ether exhibited antifungal activity. These three standards were diluted to different concentrations and cocultured with *C. gloeosporioides* in a sealed petri dish to detect their antagonistic activity. In the concentration range of 10–200 μL/L, all three standards easily volatilized and inhibited mycelium growth and conidia germination. Among them, 3-methyl-1-butanol showed the best antagonistic activity, with MICs against anthracnose mycelium and conidia of 100 and 10 μL/L, respectively, followed by 2-phenylethyl methyl ether, with MICs of 100 and 50 μL/L, respectively. For phenylethyl alcohol, the inhibitory effect on *C. gloeosporioides* mycelium growth and germination was not ideal, as its MIC was above 200 μL/L (Figure 7).

## Discussion

The VOCs from microorganisms are generally lipophilic and have low boiling points. Therefore, they can be freely released into the external environment from biofilms. Some VOCs can act as signal transducers to communicate with other organisms (Adam et al., 2017; Avalos et al., 2020). In recent years, an increasing number of studies have shown that VOCs produced by beneficial microorganisms will affect the growth of plant pathogenic fungi. However, to date, there has been no report on the production of VOCs with antifungal activity by *Rahnella* spp. Moreover, there are few reports regarding the biological activity of *Rahnella* spp. It has been reported that *R. aquatilis* ZF7 has a strong rhizosphere colonization ability and broad-spectrum plant growth-promoting activity (Yuan et al., 2019). *R. aquatilis* HX2 can control grape root cancer by producing bacteriocin (Li et al., 2014). In this study, we reported for the first time that the VOCs produced by *R. aquatilis* JZ-GX1 can inhibit the mycelium growth and spore germination of *C. gloeosporioides*.

Understanding the antagonistic characteristics of volatile microbial metabolites is helpful to further reveal the biological mechanism of antagonistic bacteria. The cell membrane is the basis for maintaining cell integrity and normal material and energy metabolism. When the cell membrane is destroyed, some intracellular proteins, phosphates, carbonates, DNA and RNA will be released, and these nuclear substances strongly absorb UV at 260 nm (Vitro et al., 2005). Chen and Cooper (2002) reported that some antimicrobial substances can destroy the cell membrane of microorganisms and increase their cell membrane permeability to achieve bacteriostasis. Therefore, a change in cell membrane integrity can be inferred by detecting the ultraviolet absorption of a suspension. In this study, as the treatment time with *R. aquatilis* JZ-GX1 VOCs increased, more nucleic acids leaked out of *C. gloeosporioides*. Similarly, MDA is one of the most important products of membrane lipid peroxidation. The degree of membrane lipid peroxidation can be determined by measuring the content of MDA, and the damage degree of the membrane system can be determined indirectly (Xu et al., 2017). Some studies have shown that the fermentation broth of *Trichoderma virens* T43 can hydrolyze the proteins and sugars of pathogenic fungi, increase the content of MDA, and eventually lead to cell death (Yin et al., 2014). In this study, the MDA content in the mycelium of *C. gloeosporioides* treated with JZ-GX1 VOCs for 4 days was significantly higher than that of the control group, and the soluble sugar content decreased in the mycelia of *C. gloeosporioides* stressed by JZ-GX1 VOCs. Therefore, it is speculated that one of the targets of JZ-GX1 VOCs is a location on the *C. gloeosporioides* cell membrane, and antagonistic effects can be achieved by destroying the integrity of the cell membrane.

In the process of infecting a host, a pathogen will efficiently regulate the expression of its own pathogenicity-related genes, thus regulating the growth of conidia or hyphae in a direction conducive to its own infection, realizing the smooth colonization of the pathogen and causing the host to be susceptible to disease (Lin et al., 2012; Upadhyay et al., 2013). For this reason, we further discussed the expression of three pathogenicity-related genes, *SCD*, *PG*, and *PKS*. Some studies have shown that the appressorium plays an important role in infection by *C. gloeosporioides*: melanin synthesis and accumulation can increase the swelling and pressure of the appressorium, thus promoting successful pathogen infection. *SCD* is one of the key enzymes in the melanin biosynthesis pathway, and the expression of this gene will affect the infection efficiency of *C. gloeosporioides* (Nosanchuk and Casadevall, 2006). *PG* in the pathogen can degrade pectin in the cell wall of the host and promote host colonization (Alkan et al., 2015). *PKS* is a key enzyme that regulates the synthesis of polyketones, secondary metabolites involved in defense or cell-to-cell communication (Noar et al., 2019). In this study, qPCR assays showed that all three key genes were downregulated by VOCs, which suggested that the VOCs of JZ-GX1 interfered with the infection activity, colonization ability and host resistance of *C. gloeosporioides*, thus reducing the degree of infection in the leaves of *Liriodendron chinense* × *tulipifera*.

To determine which VOCs produced by *R. aquatilis* JZ-GX1 inhibited the growth of *C. gloeosporioides*, the commonly used headspace SPME and GC-MS techniques were used to analyse individual VOCs. The main VOCs produced by JZ-GX1 included eight compounds: ketones, hydrocarbons, ethers, esters, and alcohols. Among them, 3-methyl-1-butanol and 2-phenylethyl methyl ether had the best antagonistic effects against *C. gloeosporioides*. Previous studies have reported the antifungal properties of 3-methyl-1-butanol. For example, the VOCs produced by the endophytic fungus *Phaeosphaeria nodorum* include 3-methyl-1-butanol, which inhibits the growth of the mycelium of peach brown rot and leads to mycelial disintegration (Pimenta et al., 2012). The median effective dose of 3-methyl-1-butanol produced by *Muscodor suthepensis* CMUCib462 was 250.29 ± 0.29 μL/L against *Penicillium digitatum* growth (Suwannarach et al., 2016). 2-Phenylethyl methyl ether has a pleasant floral fragrance, so it is widely used as an ingredient and flavoring in the food and cosmetics industries (Pan et al., 2013; Li H. H. et al., 2017). However, there are no reports about this compound in the antifungal volatile components of plants or microorganisms in the existing literature. In this study, we first reported that 2-phenylethyl methyl ether has strong inhibitory activity against *C. gloeosporioides*, but whether it has antagonistic effects against other pathogens remains to be further studied. Although the relative content of 3-methyl-1-butanol in JZ-GX1 VOCs is relatively low, 10 μL of the pure compound can inhibit the germination of *C. gloeosporioides* spores. However, phenylethyl alcohol, which accounts for a relatively high percentage of the VOC profile, did not show antifungal activity. The content of each VOC produced by a microorganism is not directly related to its inhibitory effect on pathogenic fungi: some compounds are abundant, but their inhibitory effect on pathogenic fungi is not obvious, while the content of some compounds is low, but their inhibitory effect is significant.

In summary, this study proved for the first time that VOCs produced by *Rahnella* spp. could directly inhibit the spore germination and mycelial growth of *C. gloeosporioides*, significantly reduce the contents of nucleic acids and soluble sugars in pathogenic mycelia, increase the content of MDA, destroy the integrity of the cell membrane and decrease the expression of pathogenic genes. As a result, the infection activity and vitality of the pathogen were reduced, and the occurrence and damage degree of black spot in the leaves of *Liriodendron chinense* × *tulipifera* were reduced. Two VOCs with antifungal activity in JZ-GX1 were studied and identified. Considering the wide host range of *C. gloeosporioides*, which can infect the leaves and fruits of many plants, the discovery of these antifungal compounds is of practical significance for the development of new fungicides. Furthermore, the application of strain JZ-GX1 to the storage and preservation of postharvest fruit and the biological fumigation of soil-borne diseases will also have good application prospects.

## Data Availability Statement

All datasets generated for this study are included in the article/Supplementary Material.

## Author Contributions

W-LK completed the data analysis and the first draft of the manuscript. W-LK and LR were the finishers of the experimental research. HN participated in the experimental result analysis. X-QW directed experimental design, data analysis, manuscript writing and revision. All authors read and agreed on the final text.

## Conflict of Interest

The authors declare that the research was conducted in the absence of any commercial or financial relationships that could be construed as a potential conflict of interest.

## Supplementary Material

DATA SHEET S1 Report of GC-MS spectra from CK.

DATA SHEET S2 Report of GC-MS spectra from JZ-GX1.
