# Supplementary material for: Antifungal Effects of Volatile Organic Compounds Produced by Rahnella aquatilis JZ-GX1 Against Colletotrichum gloeosporioides in Liriodendron chinense × tulipifera
Source: Front Microbiol. 2020 May 28;11:1114. doi: 10.3389/fmicb.2020.01114 (PMC7271530; doi:10.3389/fmicb.2020.01114)

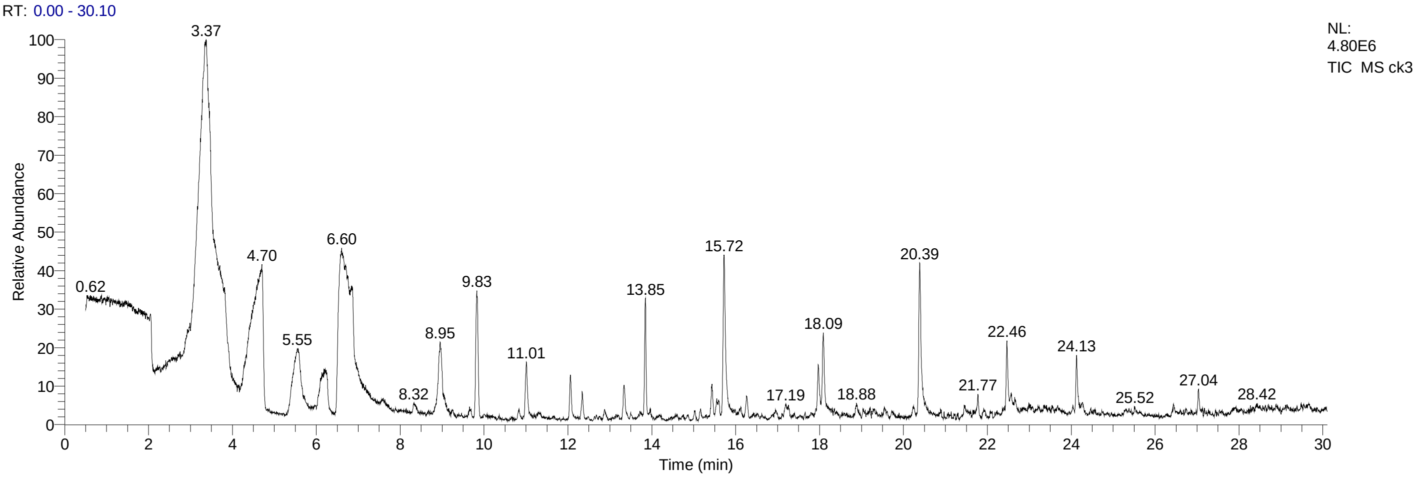

RT	Peak Area	Area %
0.54	869783.67	0.62
2.04	5736277.00	4.06
3.37	19780141.66	13.99
3.82	2608386.27	1.84
4.70	21885270.64	15.48
5.55	6358436.07	4.50
6.21	4846479.15	3.43
6.60	24656477.89	17.44
6.83	4305979.73	3.05
8.33	577945.65	0.41
8.96	4194750.88	2.97
9.83	5929593.51	4.19
11.01	2312492.00	1.64
12.06	1589347.75	1.12
12.34	942866.76	0.67
13.34	1455319.98	1.03
13.85	3897182.90	2.76
15.43	1183325.72	0.84
15.72	7143789.59	5.05
16.26	839849.30	0.59
17.97	1941735.20	1.37
18.09	3142699.86	2.22
18.88	733638.33	0.52
20.39	6362022.43	4.50
21.45	944224.41	0.67
21.77	911822.51	0.64
22.47	2355983.92	1.67
22.57	710574.26	0.50
24.13	2280333.53	1.61
27.03	903429.42	0.64

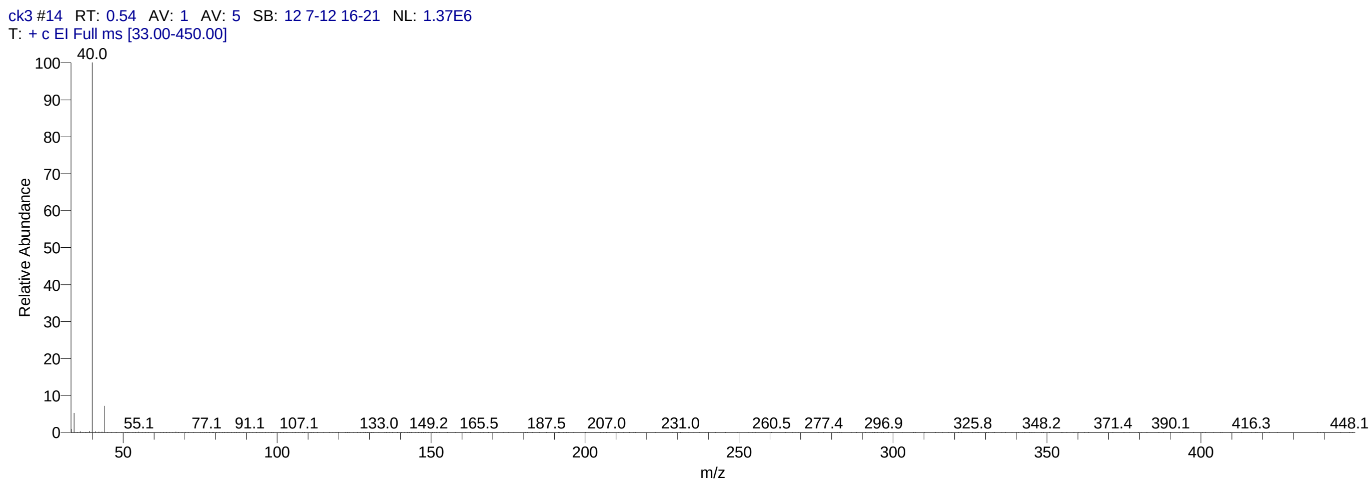

SI	Compound Name	RT	Cas #	Probability
879	Argon	0.54	7440-37-1	96.92
425	3-Butyn-1-ol	0.54	927-74-2	0.96
424	Propyne	0.54	74-99-7	0.93

Compound Structure

Argon
Formula Ar, MW 40, CAS# 7440-37-1, Entry# 1971
Ar

3-Butyn-1-ol
Formula C4H6O, MW 70, CAS# 927-74-2, Entry# 1972
1-Butyn-4-ol

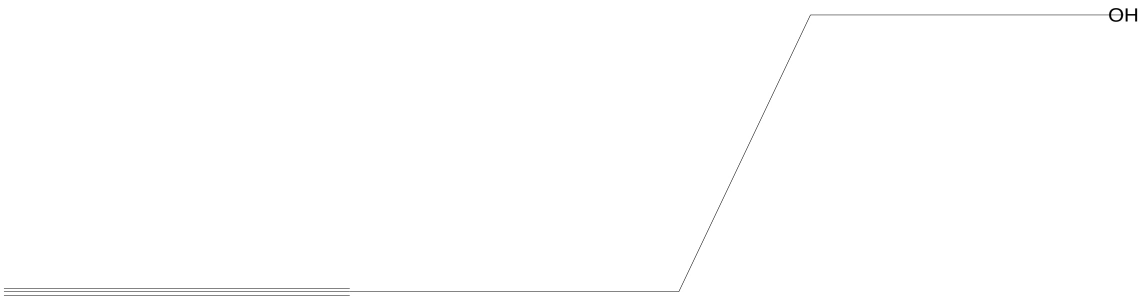

Propyne
Formula C_3H_4, MW 40, CAS# 74-99-7, Entry# 1974
Methylacetylene

ck3 #455 RT: 2.04 AV: 1 AV: 5 SB: 12 448-453 457-462 NL: 1.12E6
T: + c EI Full ms [33.00-450.00]

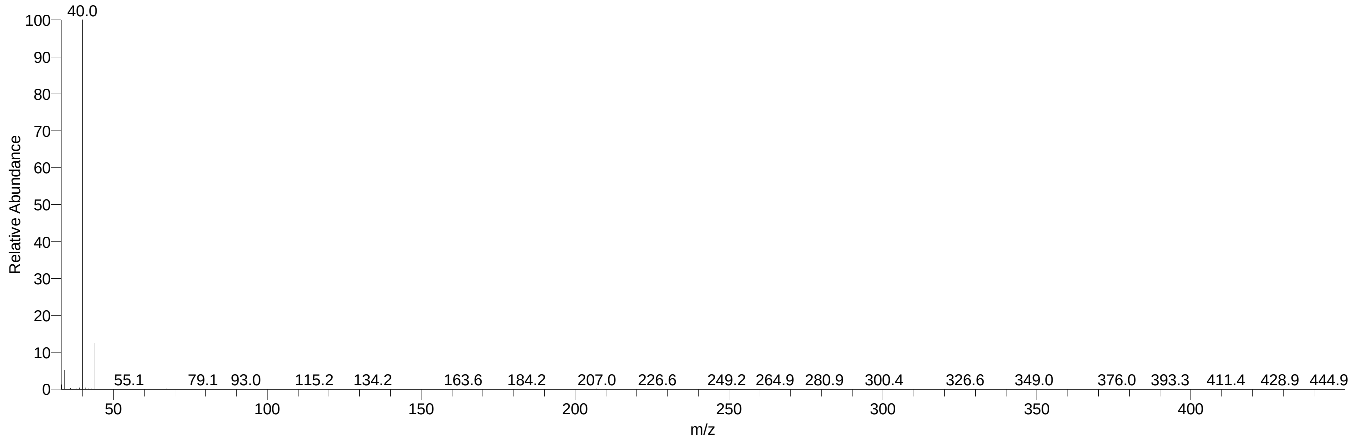

SI	Compound Name	RT	Cas #	Probability
835	Argon	2.04	7440-37-1	96.25
687	Phenethylamine, p,à-dimethyl-	2.04	64-11-9	2.77
655	Phenethylamine, p,à-dimethyl-	2.04	64-11-9	2.77

Compound Structure

Argon
Formula Ar, MW 40, CAS# 7440-37-1, Entry# 1971

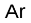

Phenethylamine, p,à-dimethyl-
Formula C10H15N, MW 149, CAS# 64-11-9, Entry# 3576
2-Amino-1-(4-methylphenyl)propane

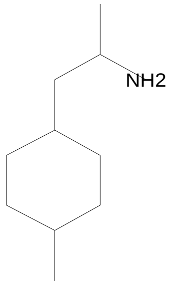

Phenethylamine, p,à-dimethyl-
Formula C10H15N, MW 149, CAS# 64-11-9, Entry# 15953
2-Amino-1-(4-methylphenyl)propane

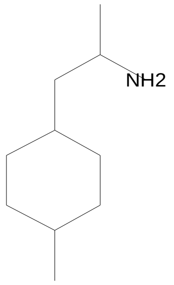

ck3 #845 RT: 3.37 AV: 1 AV: 5 SB: 12 838-843 847-852 NL: 1.50E6
T: + c EI Full ms [33.00-450.00]

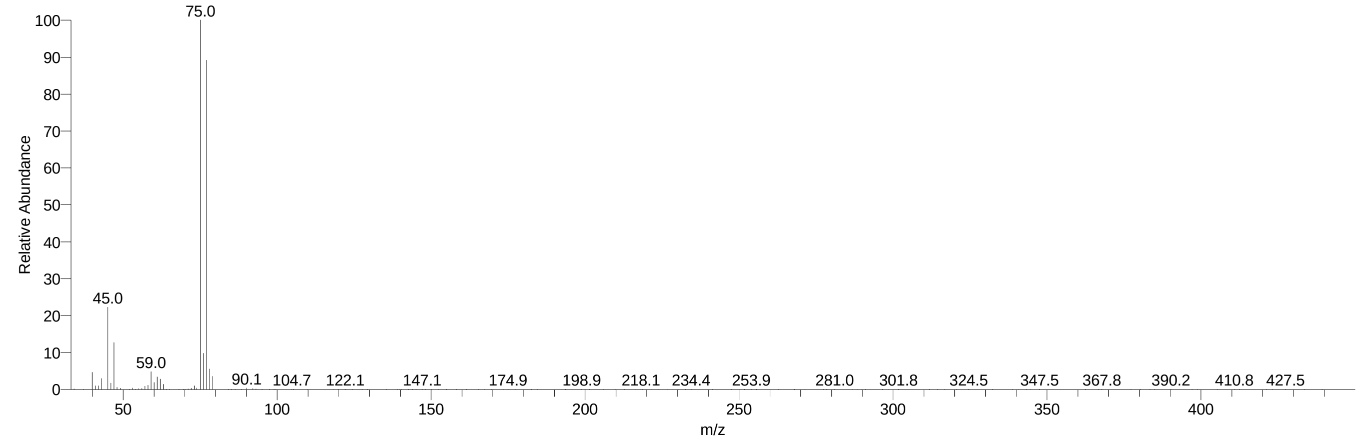

SI	Compound Name	RT	Cas #	Probability
791	2,2'-Dithiodiethanol	3.37	1892-29-1	74.73
770	2,2'-Dithiodiethanol	3.37	1892-29-1	74.73
769	2,2'-Dithiodiethanol	3.37	1892-29-1	74.73

Compound Structure

2,2'-Dithiodiethanol
Formula C4H10O2S2, MW 154, CAS# 1892-29-1, Entry# 169797
$:03[M-H]-

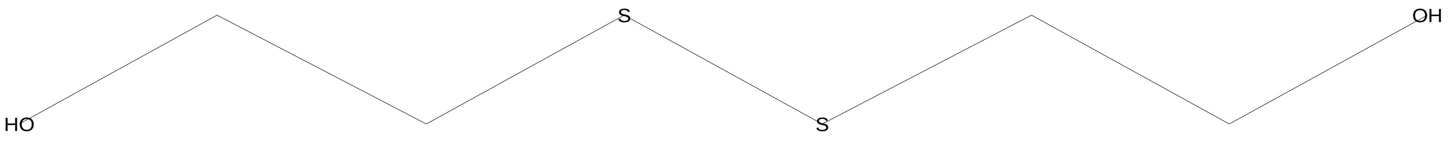

2,2'-Dithiodiethanol
Formula C4H10O2S2, MW 154, CAS# 1892-29-1, Entry# 169796
$:03[M-H]-

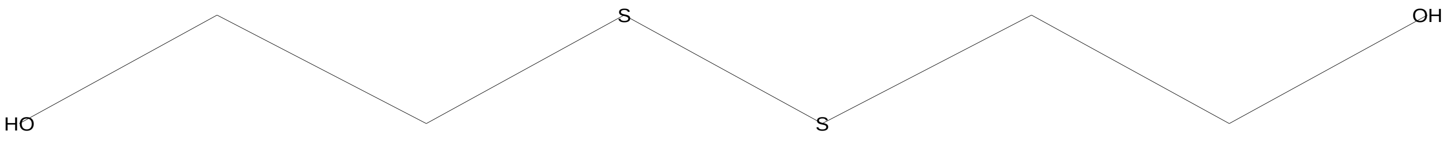

2,2'-Dithiodiethanol
Formula C4H10O2S2, MW 154, CAS# 1892-29-1, Entry# 169805
$:03[M-H]-

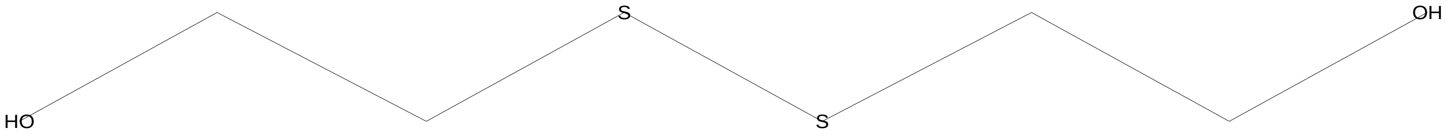

ck3 #976 RT: 3.82 AV: 1 AV: 5 SB: 12 969-974 978-983 NL: 8.18E5
T: + c EI Full ms [33.00-450.00]

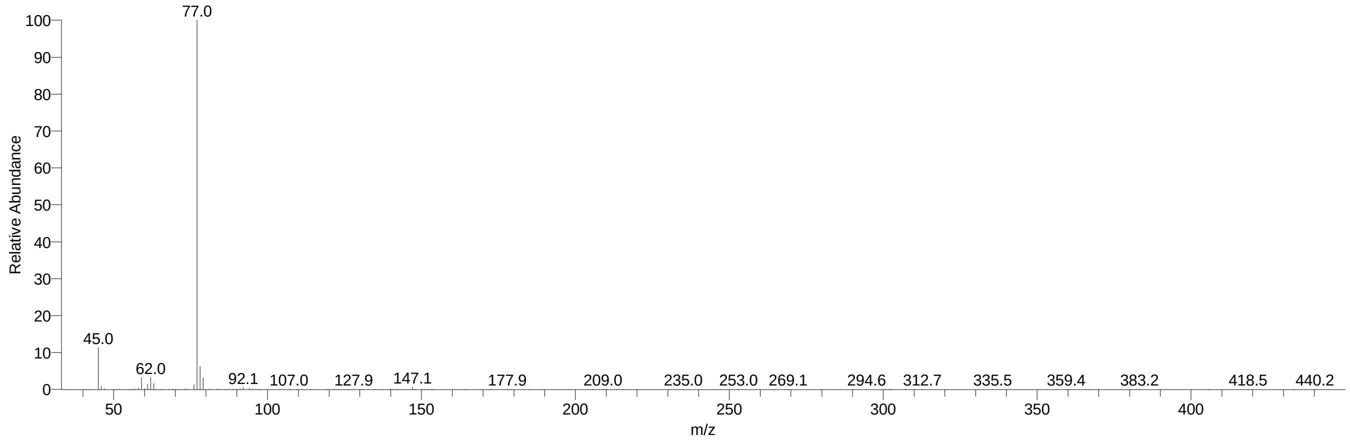

SI	Compound Name	RT	Cas #	Probability
913	Benzoic acid, 2-fluoro-	3.82	445-29-4	2.26
911	1-Phenoxy-2-propanol	3.82	770-35-4	2.08
905	Isopropyl 4-hydroxybenzoate	3.82	4191-73-5	1.64

Compound Structure

Benzoic acid, 2-fluoro-
Formula C7H5FO2, MW 140, CAS# 445-29-4, Entry# 23406
$:30[M+H]+=>97.0

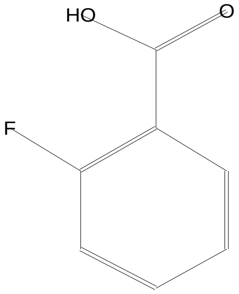

1-Phenoxy-2-propanol
Formula C9H12O2, MW 152, CAS# 770-35-4, Entry# 22447
$:30[M+H-H2O]+=>95.0

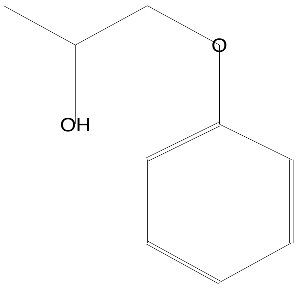

Isopropyl 4-hydroxybenzoate
Formula C10H12O3, MW 180, CAS# 4191-73-5, Entry# 23367
$:30[M+H]+=>95.0

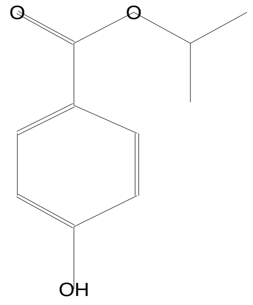

ck3 #1235 RT: 4.70 AV: 1 AV: 5 SB: 12 1228-1233 1237-1242 NL: 4.94E5
T: + c EI Full ms [33.00-450.00]

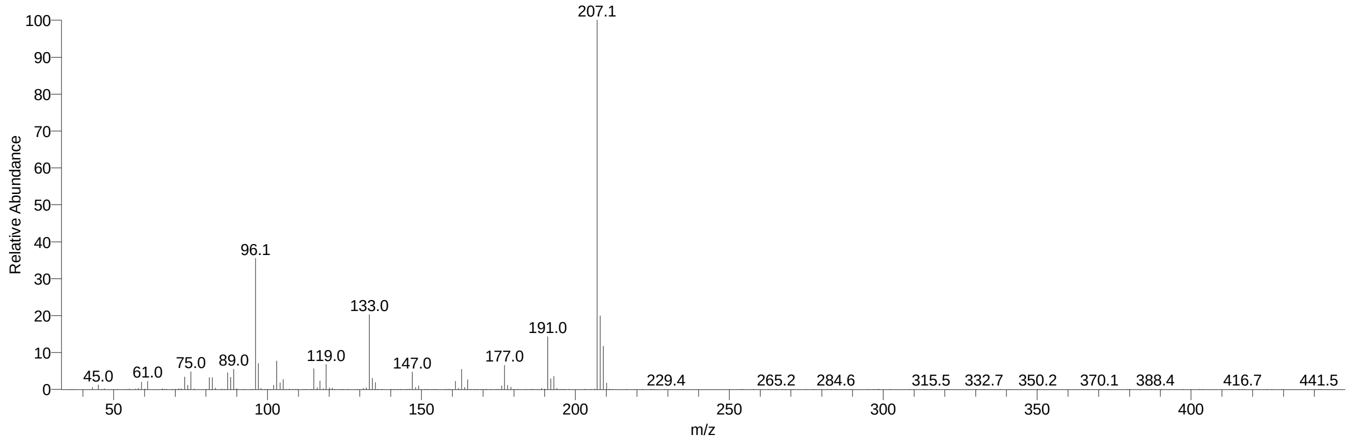

SI	Compound Name	RT	Cas #	Probability
892	Cyclotrisiloxane, hexamethyl-	4.70	541-05-9	91.08
870	Cyclotrisiloxane, hexamethyl-	4.70	541-05-9	91.08
803	Cyclotrisiloxane, hexamethyl-	4.70	541-05-9	91.08

Compound Structure

Cyclotrisiloxane, hexamethyl-
Formula C6H18O3Si3, MW 222, CAS# 541-05-9, Entry# 188945
Dimethylsiloxane cyclic trimer

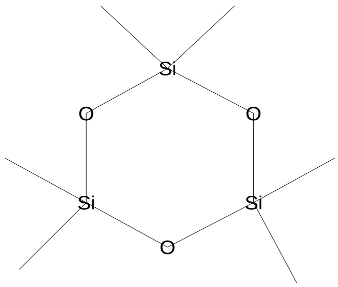

Cyclotrisiloxane, hexamethyl-
Formula C6H18O3Si3, MW 222, CAS# 541-05-9, Entry# 29125
Dimethylsiloxane cyclic trimer

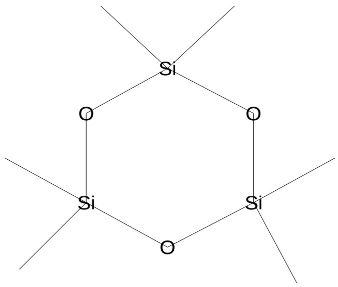

Cyclotrisiloxane, hexamethyl-
Formula C6H18O3Si3, MW 222, CAS# 541-05-9, Entry# 29098
Dimethylsiloxane cyclic trimer

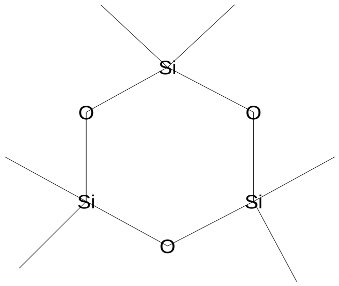

ck3 #1485 RT: 5.55 AV: 1 AV: 5 SB: 12 1478-1483 1487-1492 NL: 2.40E5
T: + c EI Full ms [33.00-450.00]

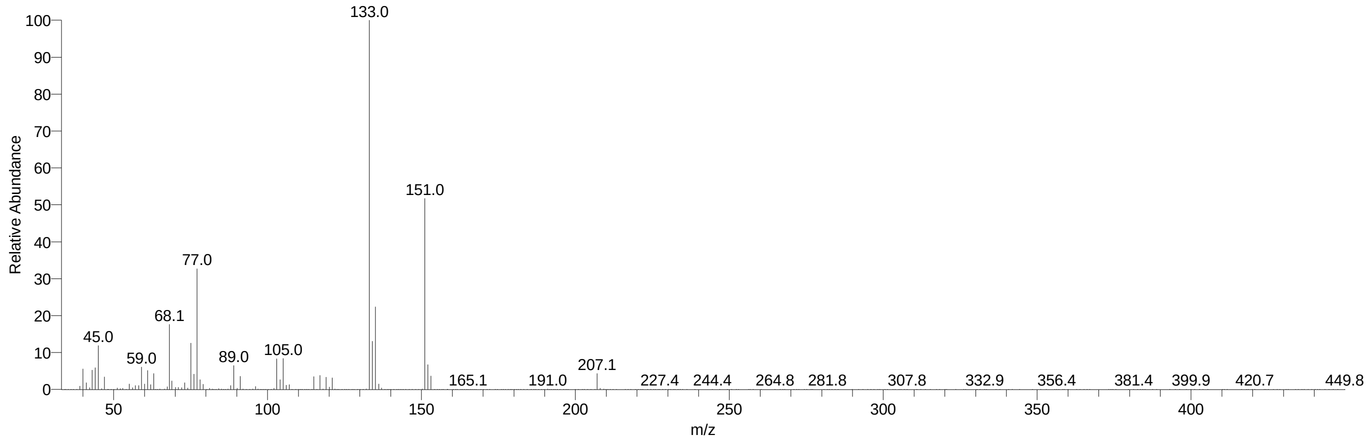

SI	Compound Name	RT	Cas #	Probability
825	Oxime-, methoxy-phenyl-_	5.55	NA	90.95
677	Esculetin	5.55	305-01-1	2.61
667	4-Ethylbenzoic acid, cyclopentyl ester	5.55	NA	1.84

Compound Structure

Oxime-, methoxy-phenyl-_
Formula C8H9NO2, MW 151, CAS# NA, Entry# 117767
Methyl N-hydroxybenzenecarboximidoate #

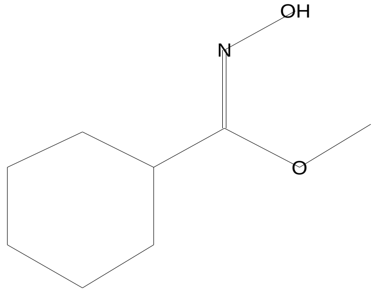

Esculetin
Formula C9H6O4, MW 178, CAS# 305-01-1, Entry# 105707
$:03[M+H]+

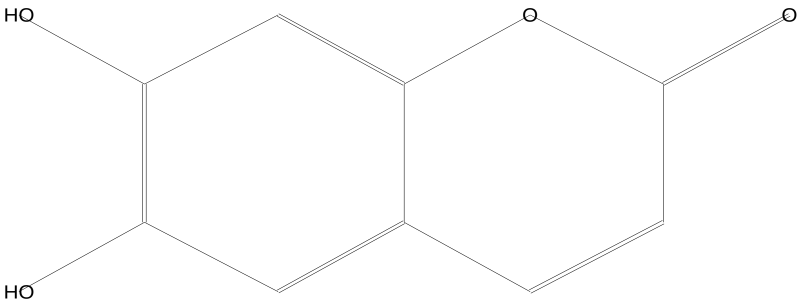

4-Ethylbenzoic acid, cyclopentyl ester
Formula C14H18O2, MW 218, CAS# NA, Entry# 117763
Cyclopentyl 4-ethylbenzoate #

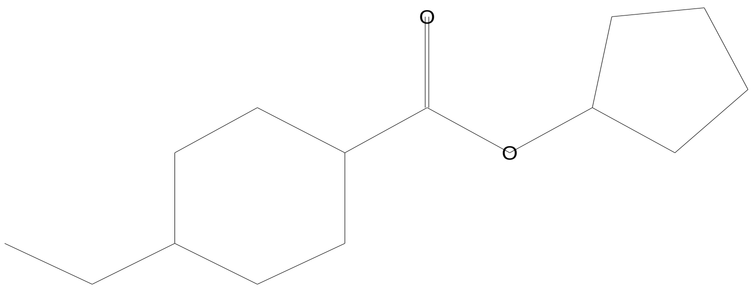

ck3 #1680 RT: 6.21 AV: 1 AV: 5 SB: 12 1673-1678 1682-1687 NL: 1.33E5
T: + c EI Full ms [33.00-450.00]

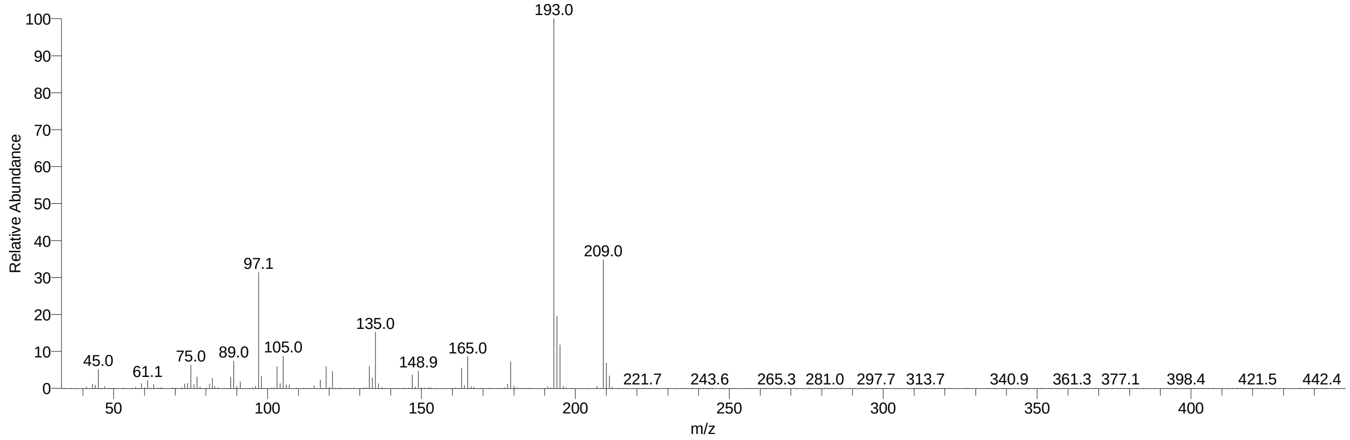

SI	Compound Name	RT	Cas #	Probability
620	Vanillin, TBDMS derivative	6.21	NA	11.53
617	2,5-Dimethoxy-4-methylamphetamine	6.21	15588-95-1	10.18
615	Isovanillin, TBDMS derivative	6.21	NA	9.39

Compound Structure

Vanillin, TBDMS derivative
Formula C14H22O3Si, MW 266, CAS# NA, Entry# 180564
Vanillin, tert-butyldimethylsilyl ether

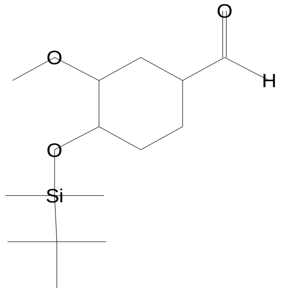

2,5-Dimethoxy-4-methylamphetamine
Formula C12H19NO2, MW 209, CAS# 15588-95-1, Entry# 185799
$:03[M+H]+

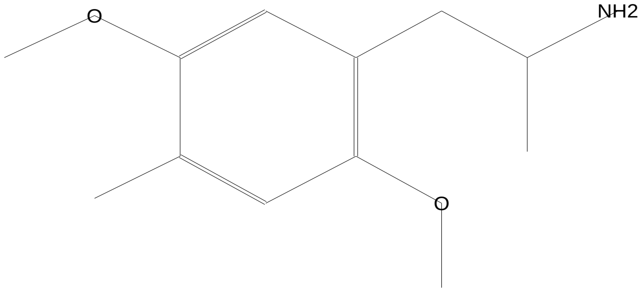

Isovanillin, TBDMS derivative
Formula C14H22O3Si, MW 266, CAS# NA, Entry# 180565
3-Hydroxy-4-methoxybenzaldehyde, tert-butyldimethylsilyl ether

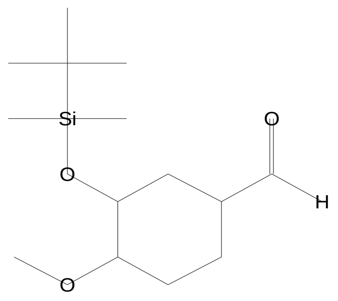

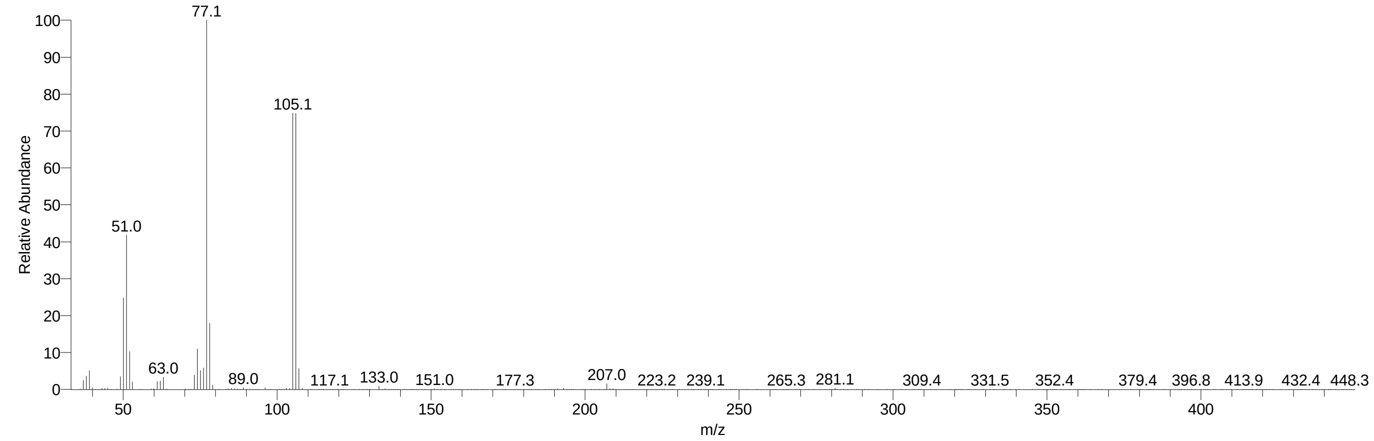

SI	Compound Name	RT	Cas #	Probability
921	Benzaldehyde	6.60	100-52-7	74.06
905	Benzaldehyde	6.60	100-52-7	74.06
898	Benzaldehyde	6.60	100-52-7	74.06

Compound Structure

Benzaldehyde
Formula C7H6O, MW 106, CAS# 100-52-7, Entry# 10911
Artificial Almond Oil

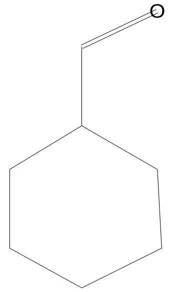

Benzaldehyde
Formula C7H6O, MW 106, CAS# 100-52-7, Entry# 16610
Artificial Almond Oil

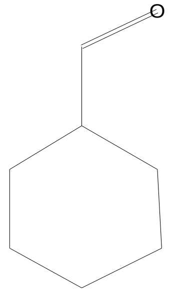

Benzaldehyde
Formula C_7H_6O, MW 106, CAS# 100-52-7, Entry# 1633
Artificial Almond Oil

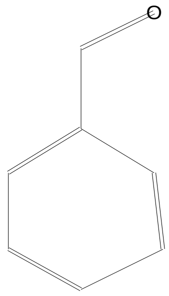

ck3 #1863 RT: 6.83 AV: 1 AV: 5 SB: 12 1856-1861 1865-1870 NL: 1.87E5
T: + c EI Full ms [33.00-450.00]

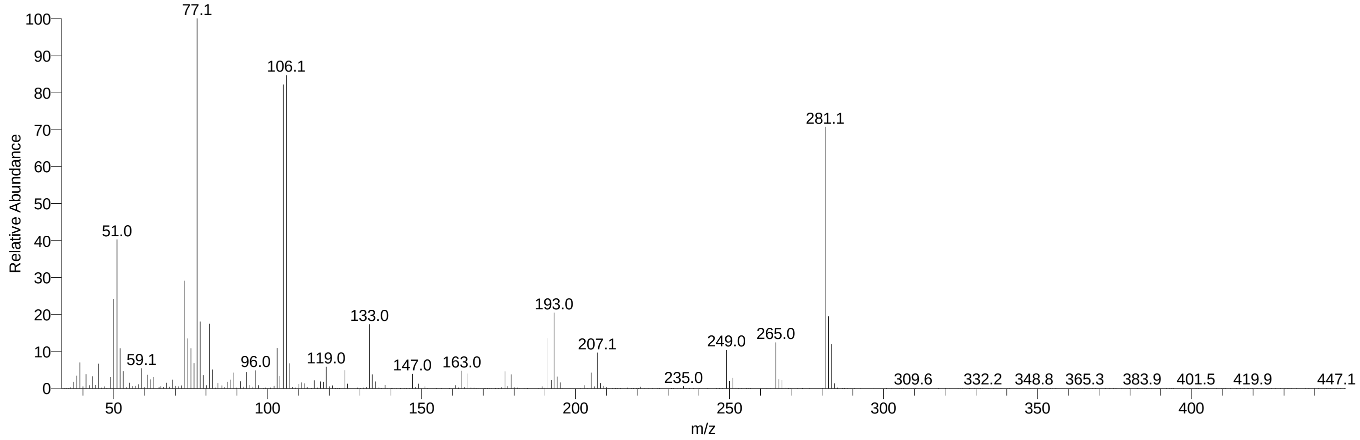

SI	Compound Name	RT	Cas #	Probability
786	Glafenin	6.83	3820-67-5	83.72
689	Staurosporine	6.83	62996-74-1	6.56
689	Glafenin	6.83	3820-67-5	83.72

Compound Structure

Glafenin
Formula C19H17ClN2O4, MW 372, CAS# 3820-67-5, Entry# 22947
$:30[M+H-H2O]+=>299.0

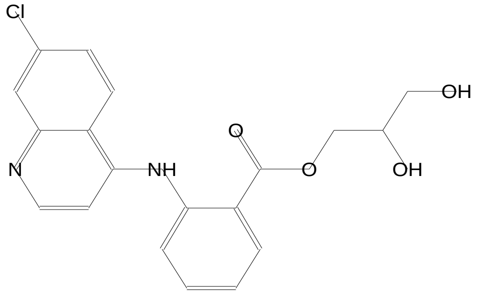

Glafenin
Formula C19H17ClN2O4, MW 372, CAS# 3820-67-5, Entry# 187453
$:03[M+H]+

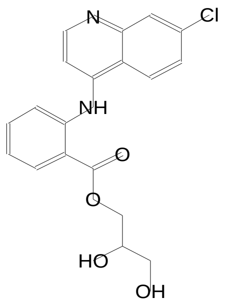

Staurosporine
Formula C28H26N4O3, MW 466, CAS# 62996-74-1, Entry# 8303
$:30[M+H]+=>337.9=>309.1

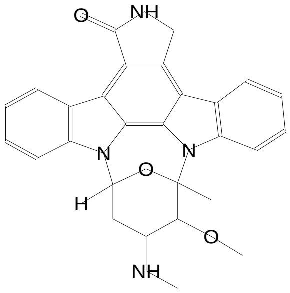

ck3 #2303 RT: 8.33 AV: 1 AV: 5 SB: 12 2296-2301 2305-2310 NL: 2.59E4
T: + c EI Full ms [33.00-450.00]

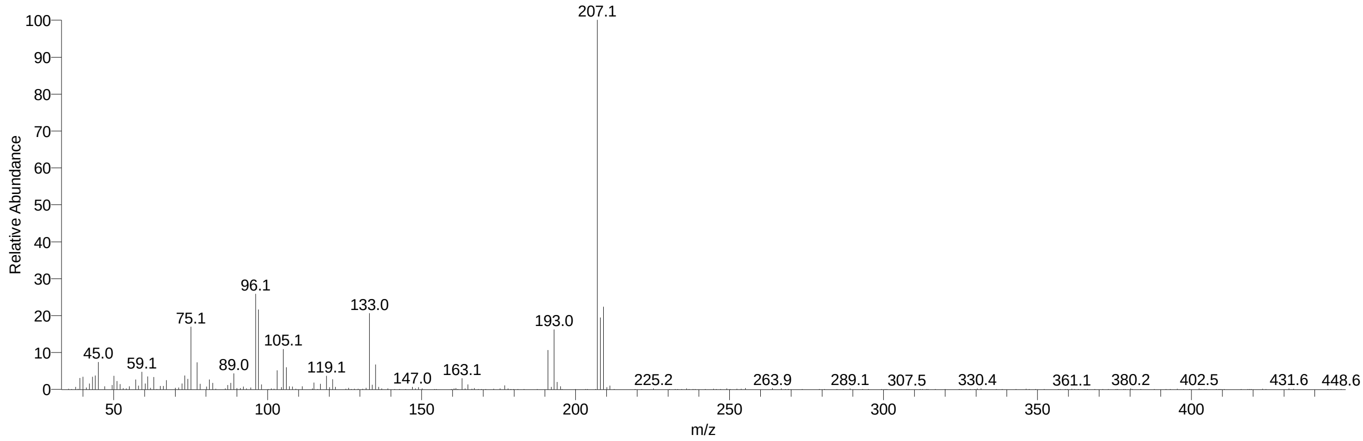

SI	Compound Name	RT	Cas #	Probability
742	Cyclotrisiloxane, hexamethyl-	8.33	541-05-9	26.28
740	Cyclotrisiloxane, hexamethyl-	8.33	541-05-9	26.28
734	Arsenous acid, tris(trimethylsilyl) ester	8.33	55429-29-3	19.60

Compound Structure

Cyclotrisiloxane, hexamethyl-
Formula C6H18O3Si3, MW 222, CAS# 541-05-9, Entry# 188945
Dimethylsiloxane cyclic trimer

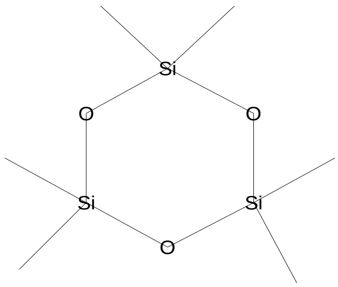

Cyclotrisiloxane, hexamethyl-
Formula C6H18O3Si3, MW 222, CAS# 541-05-9, Entry# 29125
Dimethylsiloxane cyclic trimer

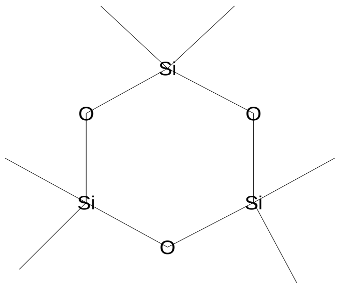

Arsenous acid, tris(trimethylsilyl) ester
Formula C9H27AsO3Si3, MW 342, CAS# 55429-29-3, Entry# 188948
$:28CWZNQWMMDOIFQC-UHFFFAOYSA-N

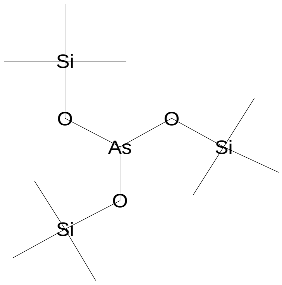

ck3 #2487 RT: 8.96 AV: 1 AV: 5 SB: 12 2480-2485 2489-2494 NL: 1.65E5
T: + c EI Full ms [33.00-450.00]

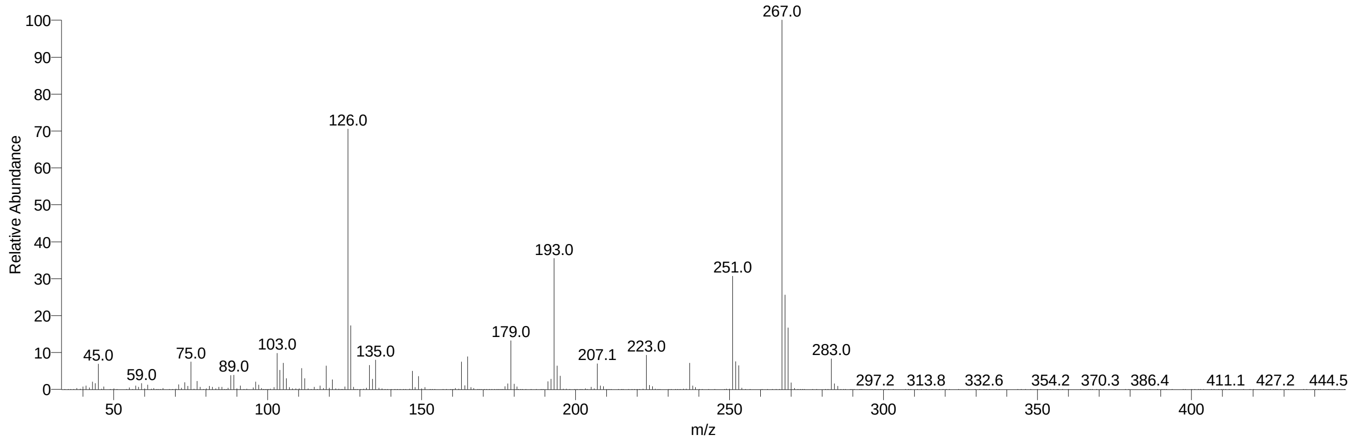

SI	Compound Name	RT	Cas #	Probability
715	5-Hydroxy-3'-methoxyflavone	8.96	6697-60-5	51.43
693	6-Hydroxy-2'-methoxyflavone	8.96	61546-59-6	20.33
692	5-Hydroxy-3'-methoxyflavone	8.96	6697-60-5	51.43

Compound Structure

5-Hydroxy-3'-methoxyflavone
Formula C16H12O4, MW 268, CAS# 6697-60-5, Entry# 74865
$:03[M-H]-

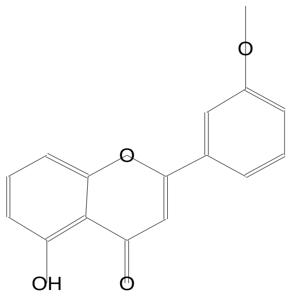

6-Hydroxy-2'-methoxyflavone
Formula C16H12O4, MW 268, CAS# 61546-59-6, Entry# 74879
$:03[M-H]-

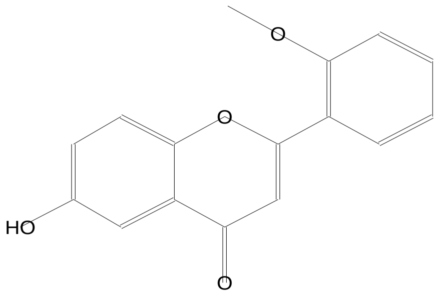

5-Hydroxy-3'-methoxyflavone
Formula C16H12O4, MW 268, CAS# 6697-60-5, Entry# 74864
$:03[M-H]-

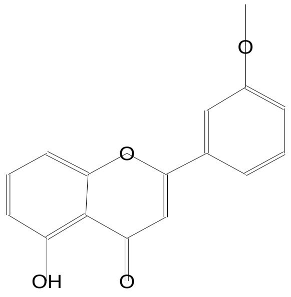

ck3 #2744 RT: 9.83 AV: 1 AV: 5 SB: 12 2737-2742 2746-2751 NL: 6.88E5
T: + c EI Full ms [33.00-450.00]

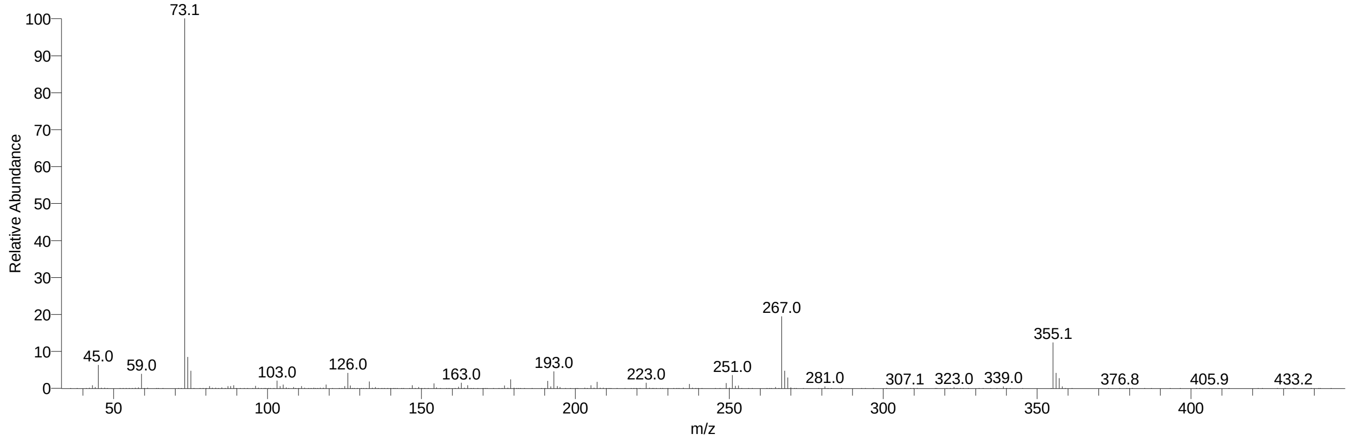

SI	Compound Name	RT	Cas #	Probability
783	Cyclopentasiloxane, decamethyl-	9.83	541-02-6	82.14
764	Cyclopentasiloxane, decamethyl-	9.83	541-02-6	82.14
728	Cyclopentasiloxane, decamethyl-	9.83	541-02-6	82.14

Compound Structure

Cyclopentasiloxane, decamethyl-
Formula C10H30O5Si5, MW 370, CAS# 541-02-6, Entry# 10279
Decamethylcyclopentasiloxane

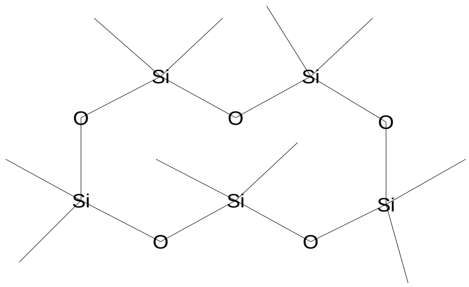

Cyclopentasiloxane, decamethyl-
Formula C10H30O5Si5, MW 370, CAS# 541-02-6, Entry# 33275
Decamethylcyclopentasiloxane

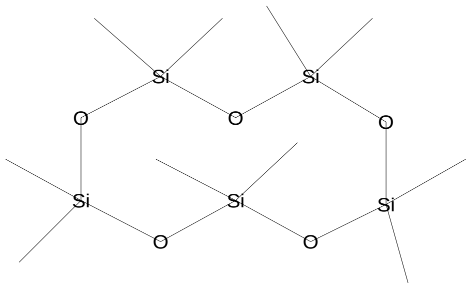

Cyclopentasiloxane, decamethyl-
Formula C10H30O5Si5, MW 370, CAS# 541-02-6, Entry# 234336
Decamethylcyclopentasiloxane

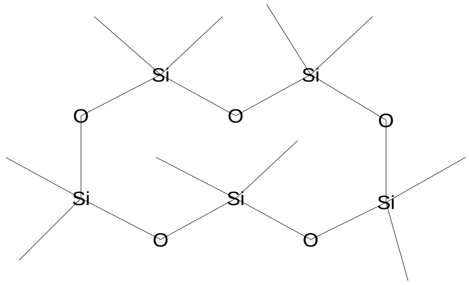

ck3 #3090 RT: 11.01 AV: 1 AV: 5 SB: 12 3083-3088 3092-3097 NL: 1.68E5
T: + c EI Full ms [33.00-450.00]

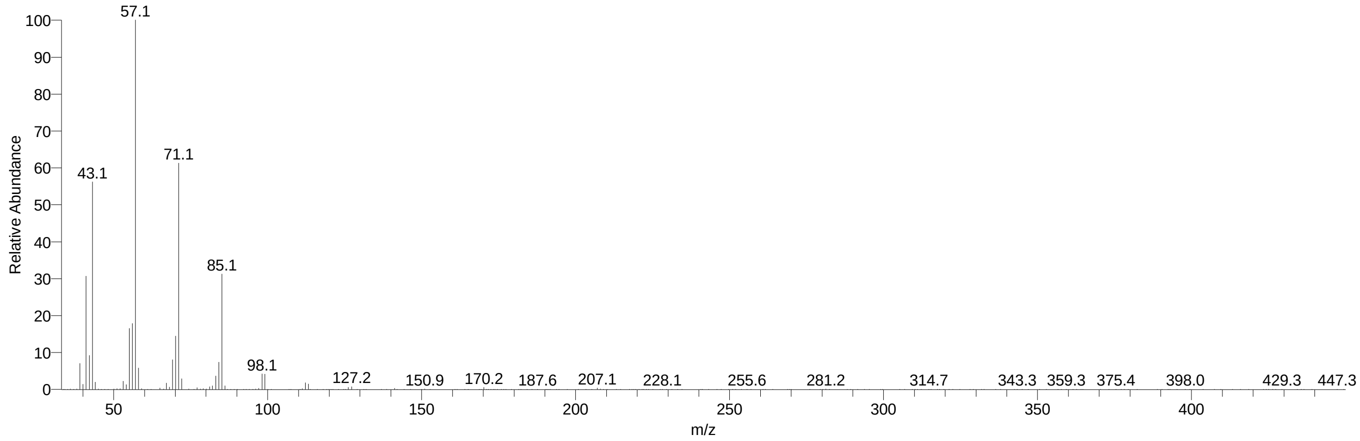

SI	Compound Name	RT	Cas #	Probability
888	Dodecane	11.01	112-40-3	10.94
888	Dodecane	11.01	112-40-3	10.94
885	Undecane	11.01	1120-21-4	9.66

Compound Structure

Dodecane
Formula C12H26, MW 170, CAS# 112-40-3, Entry# 190
n-Dodecane

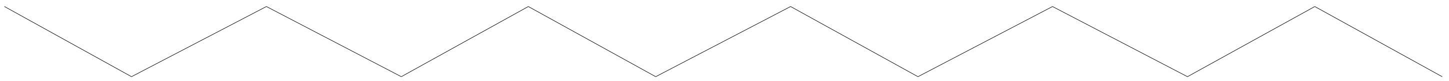

Dodecane
Formula C12H26, MW 170, CAS# 112-40-3, Entry# 24230
n-Dodecane

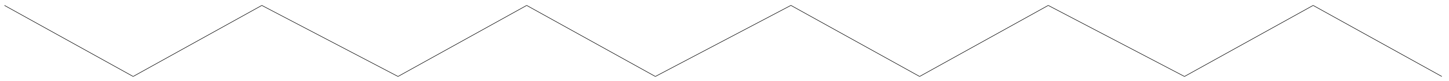

Undecane
Formula $C_{11}H_{24}$, MW 156, CAS# 1120-21-4, Entry# 6157
n-Undecane

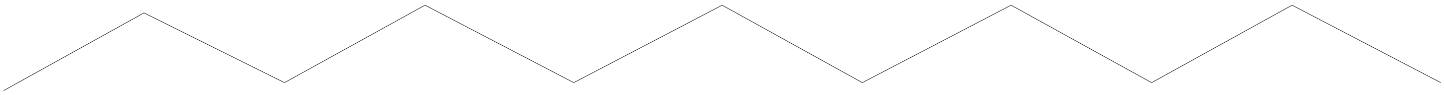

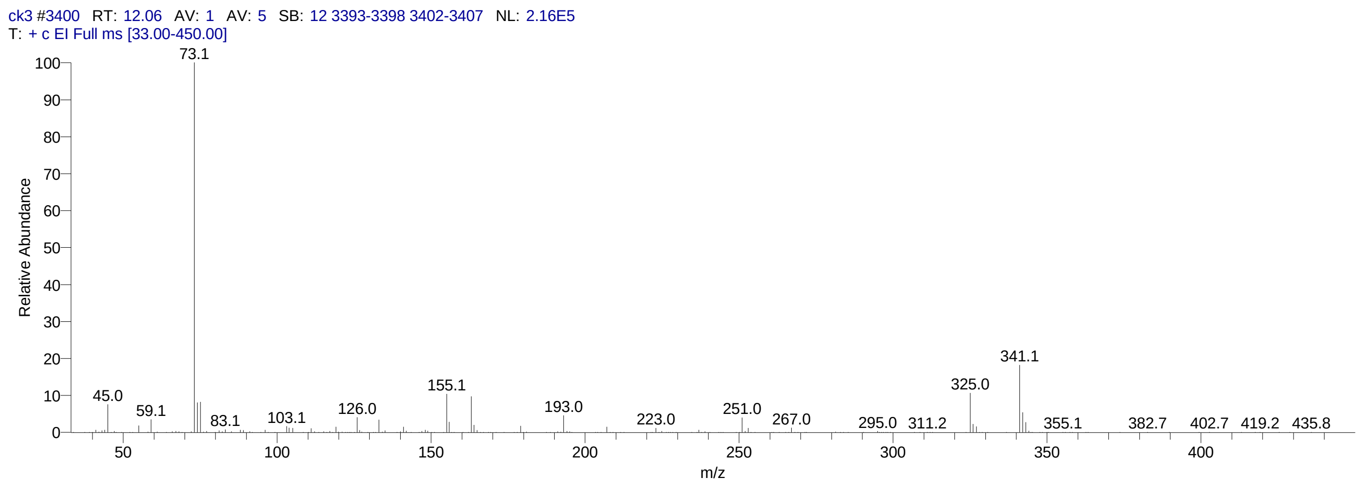

SI	Compound Name	RT	Cas #	Probability
589	Phosphonoacetic Acid, 3TMS derivative	12.06	53044-27-2	63.12
520	Cyclohexasiloxane, dodecamethyl-	12.06	540-97-6	9.38
515	Cyclohexasiloxane, dodecamethyl-	12.06	540-97-6	9.38

Compound Structure

Phosphonoacetic Acid, 3TMS derivative
Formula C11H29O5PSi3, MW 356, CAS# 53044-27-2, Entry# 232513
Acetic acid, [bis[(trimethylsilyl)oxy]phosphinyl]-, trimethylsilyl ester

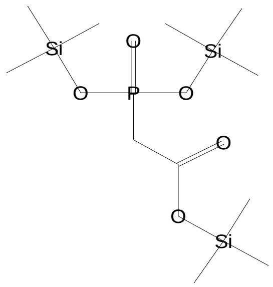

Cyclohexasiloxane, dodecamethyl-
Formula C12H36O6Si6, MW 444, CAS# 540-97-6, Entry# 10270
Dodecamethylcyclohexasiloxane

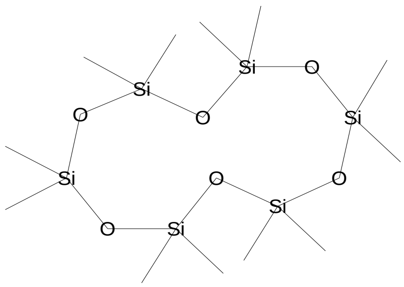

Cyclohexasiloxane, dodecamethyl-
Formula C12H36O6Si6, MW 444, CAS# 540-97-6, Entry# 10269
Dodecamethylcyclohexasiloxane

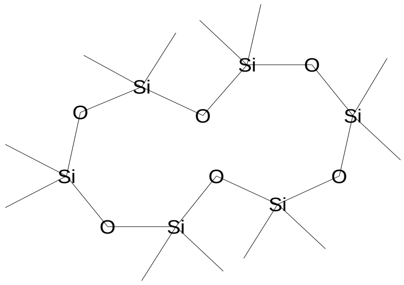

ck3 #3483 RT: 12.34 AV: 1 AV: 5 SB: 12 3476-3481 3485-3490 NL: 1.38E5
T: + c EI Full ms [33.00-450.00]

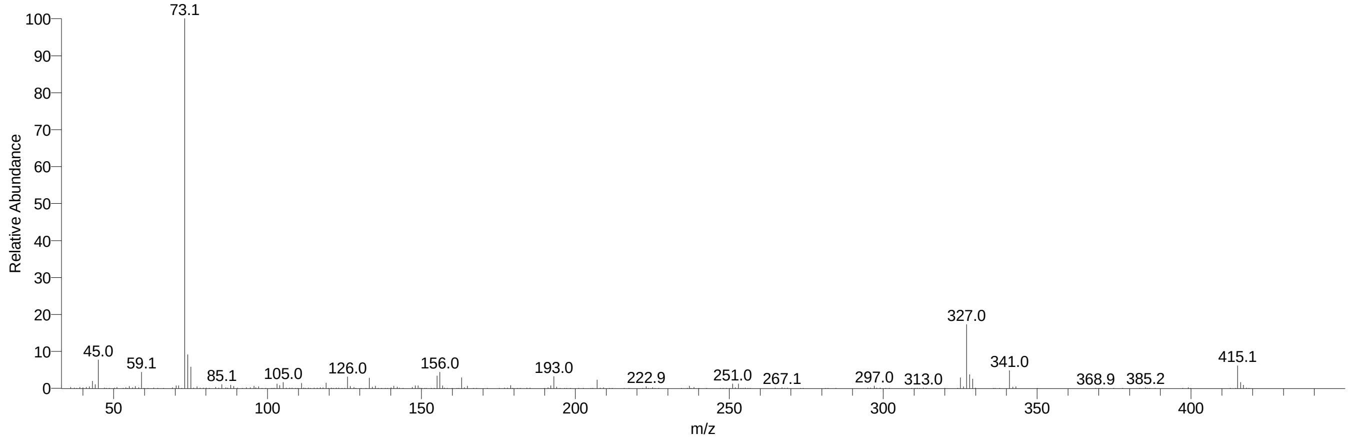

SI	Compound Name	RT	Cas #	Probability
619	3,5-Dibutoxy-1,1,1,7,7,7-hexamethyl-3,5-bis(trimethylsiloxy)tetrasiloxane	12.34	72439-85-1	46.87
607	3,5-Diisopropoxy-1,1,1,7,7,7-hexamethyl-3,5-bis(trimethylsiloxy)tetrasiloxane	12.34	71579-67-4	31.21
581	Tetrasiloxane, 3,5-diethoxy-1,1,1,7,7,7-hexamethyl-3,5-bis(trimethylsiloxy)-	12.34	72439-78-2	9.35

Compound Structure

3,5-Dibutoxy-1,1,1,7,7,7-hexamethyl-3,5-bis(trimethylsiloxy)tetrasiloxane
Formula C20H54O7Si6, MW 574, CAS# 72439-85-1, Entry# 40543
1-Butoxy-3,3,3-trimethyl-1-[(trimethylsilyl)oxy]disiloxanyl butyl bis(trimethylsilyl) orthosilicate #

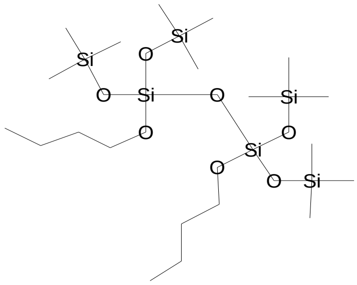

3,5-Diisopropoxy-1,1,1,7,7,7-hexamethyl-3,5-bis(trimethylsiloxy)tetrasiloxane
Formula C18H50O7Si6, MW 546, CAS# 71579-67-4, Entry# 40678
1-Isopropoxy-3,3,3-trimethyl-1-[(trimethylsilyl)oxy]disiloxanyl isopropyl bis(trimethylsilyl) orthosilicate #

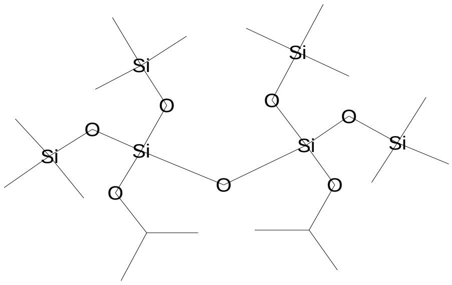

Tetrasiloxane, 3,5-diethoxy-1,1,1,7,7,7-hexamethyl-3,5-bis(trimethylsiloxy)-
Formula C16H46O7Si6, MW 518, CAS# 72439-78-2, Entry# 44057
1-Ethoxy-3,3,3-trimethyl-1-[(trimethylsilyl)oxy]disiloxanyl ethyl bis(trimethylsilyl) orthosilicate #

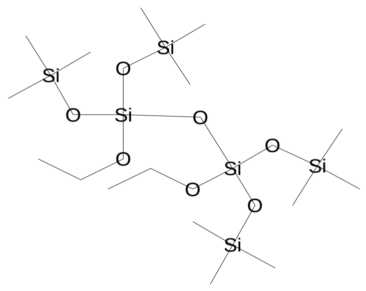

ck3 #3776 RT: 13.34 AV: 1 AV: 5 SB: 12 3769-3774 3778-3783 NL: 9.65E4
T: + c EI Full ms [33.00-450.00]

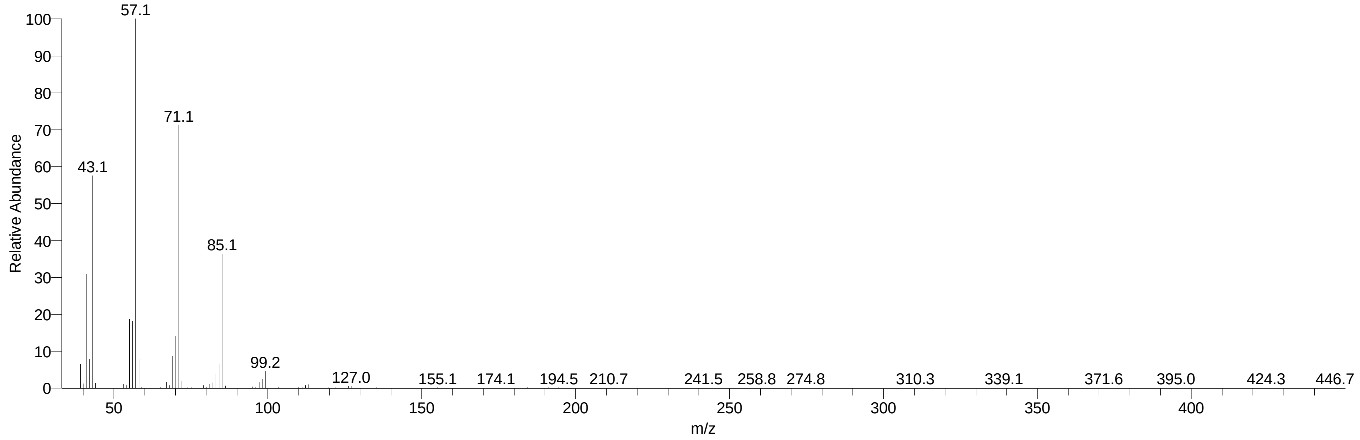

SI	Compound Name	RT	Cas #	Probability
883	Hexadecane	13.34	544-76-3	9.24
880	Hexadecane	13.34	544-76-3	9.24
878	Tetradecane	13.34	629-59-4	7.45

Compound Structure

Hexadecane
Formula C16H34, MW 226, CAS# 544-76-3, Entry# 6165
n-Cetane

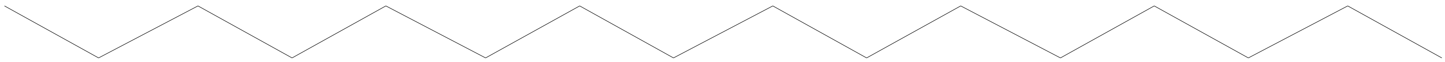

Hexadecane
Formula C16H34, MW 226, CAS# 544-76-3, Entry# 6166
n-Cetane

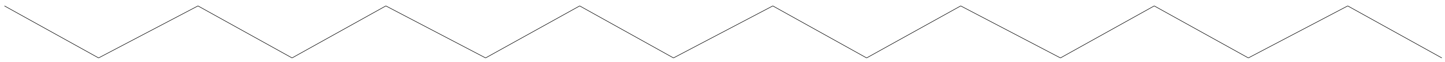

Tetradecane
Formula $C_{14}H_{30}$, MW 198, CAS# 629-59-4, Entry# 6116
n-Tetradecane

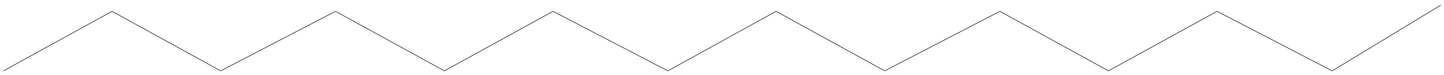

ck3 #3925 RT: 13.85 AV: 1 AV: 5 SB: 12 3918-3923 3927-3932 NL: 6.10E5
T: + c EI Full ms [33.00-450.00]

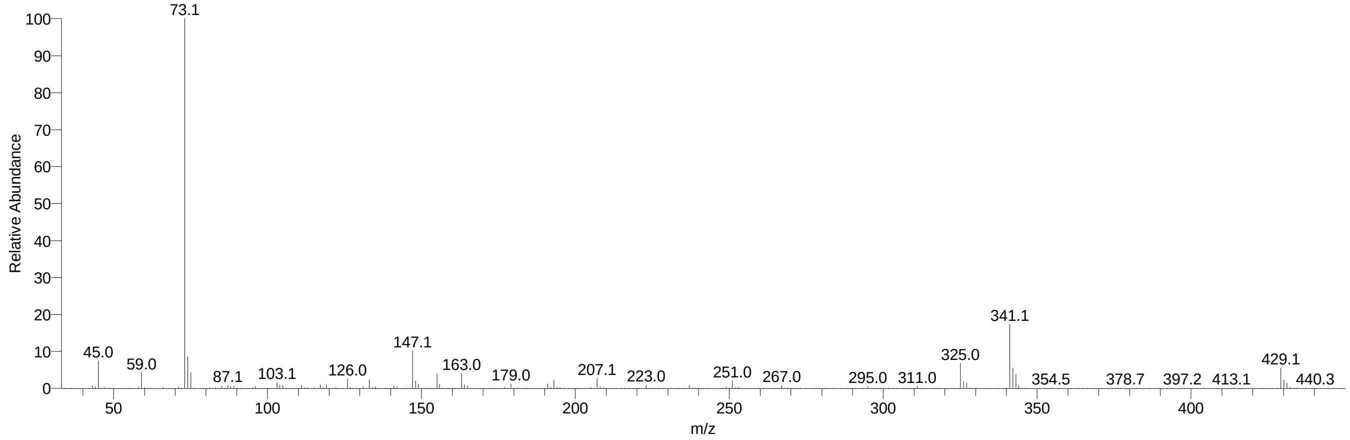

SI	Compound Name	RT	Cas #	Probability
775	Cyclohexasiloxane, dodecamethyl-	13.85	540-97-6	95.48
773	Cyclohexasiloxane, dodecamethyl-	13.85	540-97-6	95.48
755	Cyclohexasiloxane, dodecamethyl-	13.85	540-97-6	95.48

Compound Structure

Cyclohexasiloxane, dodecamethyl-
Formula C12H36O6Si6, MW 444, CAS# 540-97-6, Entry# 10270
Dodecamethylcyclohexasiloxane

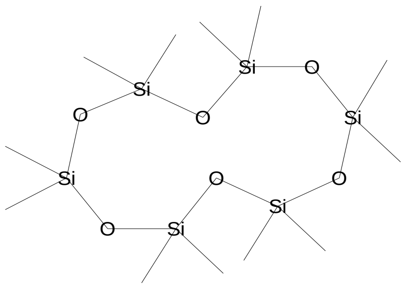

Cyclohexasiloxane, dodecamethyl-
Formula C12H36O6Si6, MW 444, CAS# 540-97-6, Entry# 44297
Dodecamethylcyclohexasiloxane

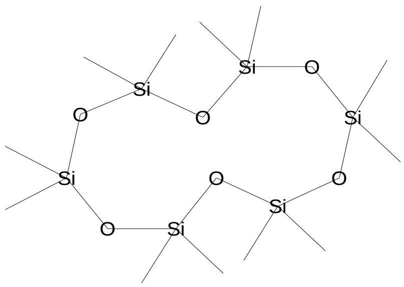

Cyclohexasiloxane, dodecamethyl-
Formula C12H36O6Si6, MW 444, CAS# 540-97-6, Entry# 10269
Dodecamethylcyclohexasiloxane

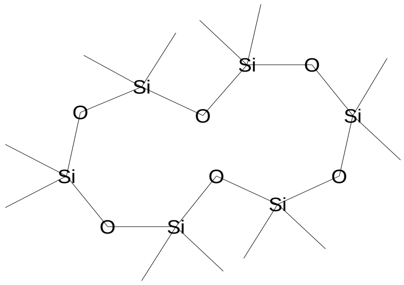

ck3 #4391 RT: 15.43 AV: 1 AV: 5 SB: 12 4384-4389 4393-4398 NL: 4.33E4
T: + c EI Full ms [33.00-450.00]

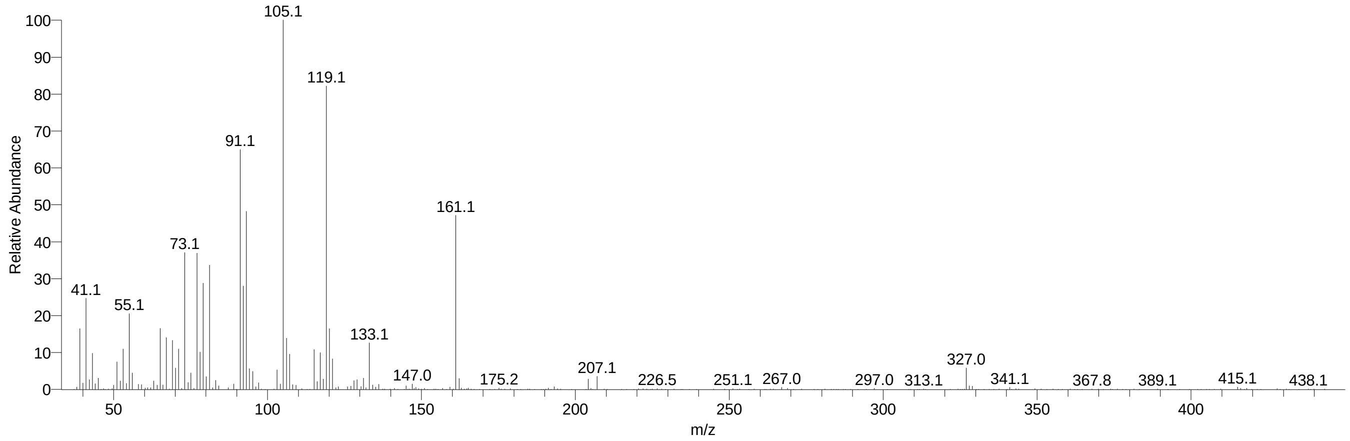

SI	Compound Name	RT	Cas #	Probability
790	Copaene	15.43	3856-25-5	21.99
764	Copaene	15.43	3856-25-5	21.99
758	Germacrene D	15.43	23986-74-5	5.87

Compound Structure

Copaene
Formula C15H24, MW 204, CAS# 3856-25-5, Entry# 18672
Tricyclo[4.4.0.02,7]dec-3-ene, 1,3-dimethyl-8-(1-methylethyl)-, stereoisomer

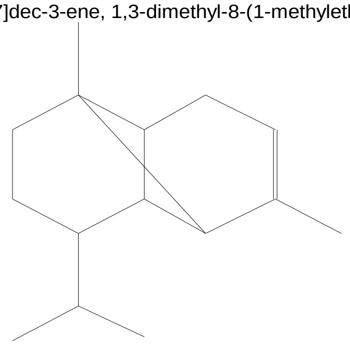

Copaene
Formula C15H24, MW 204, CAS# 3856-25-5, Entry# 24894
Tricyclo[4.4.0.02,7]dec-3-ene, 1,3-dimethyl-8-(1-methylethyl)-, stereoisomer

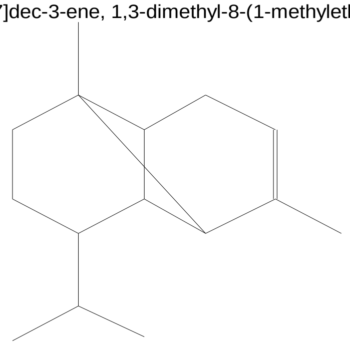

Germacrene D
Formula C15H24, MW 204, CAS# 23986-74-5, Entry# 150487
(S,1Z,6Z)-8-Isopropyl-1-methyl-5-methylenecyclodeca-1,6-diene

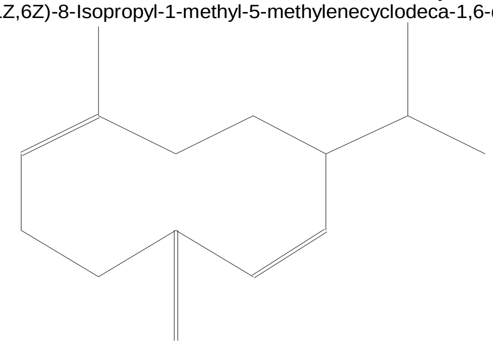

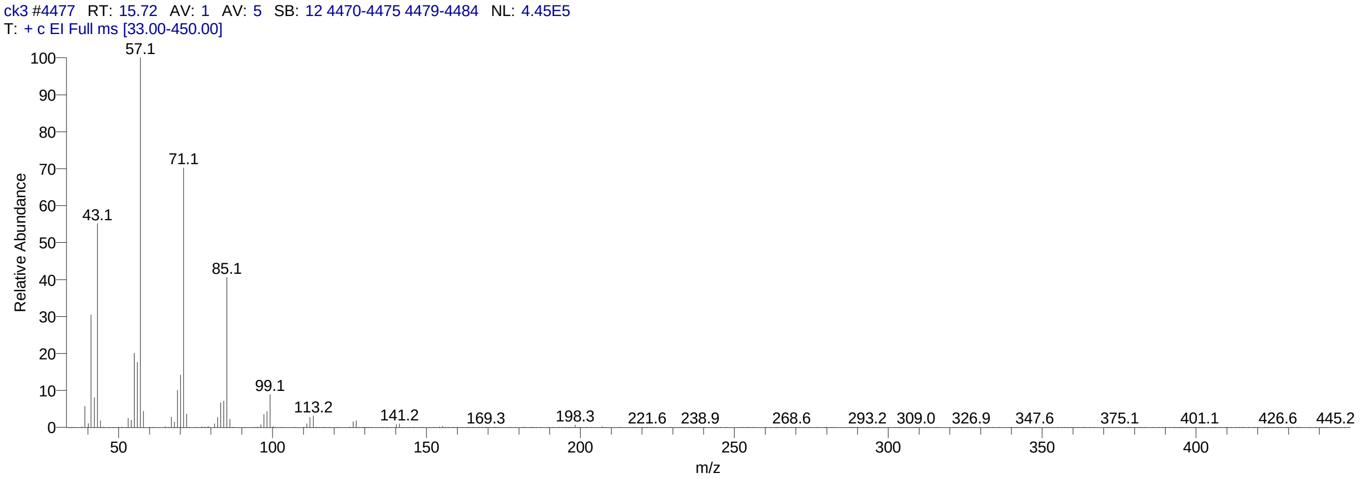

SI	Compound Name	RT	Cas #	Probability
911	Tetradecane	15.72	629-59-4	16.88
903	Tetradecane	15.72	629-59-4	16.88
897	Tetradecane	15.72	629-59-4	16.88

Compound Structure

Tetradecane
Formula C14H30, MW 198, CAS# 629-59-4, Entry# 6116
n-Tetradecane

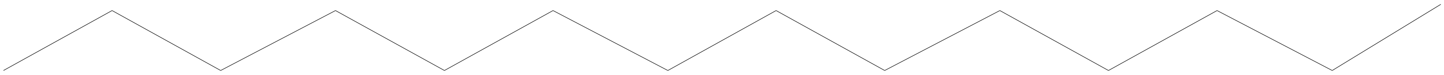

Tetradecane
Formula C14H30, MW 198, CAS# 629-59-4, Entry# 6117
n-Tetradecane

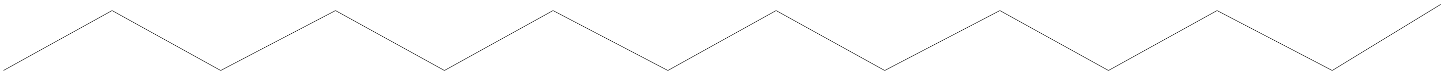

Tetradecane
Formula C14H30, MW 198, CAS# 629-59-4, Entry# 6115
n-Tetradecane

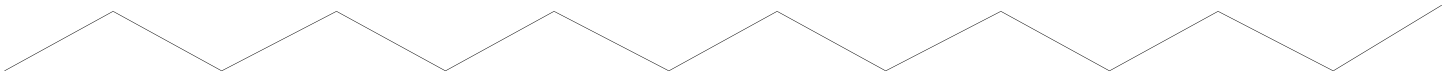

ck3 #4636 RT: 16.26 AV: 1 AV: 5 SB: 12 4629-4634 4638-4643 NL: 2.35E4
T: + c EI Full ms [33.00-450.00]

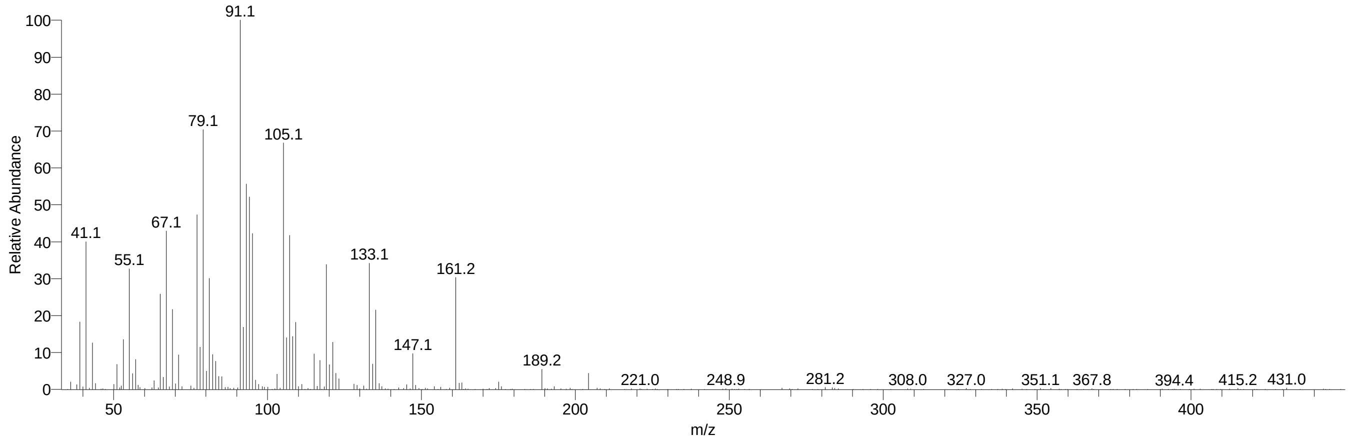

SI	Compound Name	RT	Cas #	Probability
774	Bicyclo[5.2.0]nonane, 2-methylene-4,8,8-trimethyl-4-vinyl-	16.26	242794-76-9	5.38
773	Longifolene	16.26	475-20-7	5.17
773	1,4-Methanocycloocta[d]pyridazine, 1,4,4a,5,6,9,10,10a-octahydro-11,11-dimethyl-, (1à,4à,4aà,10aà)-	16.26	NA	5.17

Compound Structure

Bicyclo[5.2.0]nonane, 2-methylene-4,8,8-trimethyl-4-vinyl-
Formula C15H24, MW 204, CAS# 242794-76-9, Entry# 66571
4,8,8-Trimethyl-2-methylene-4-vinylbicyclo[5.2.0]nonane #

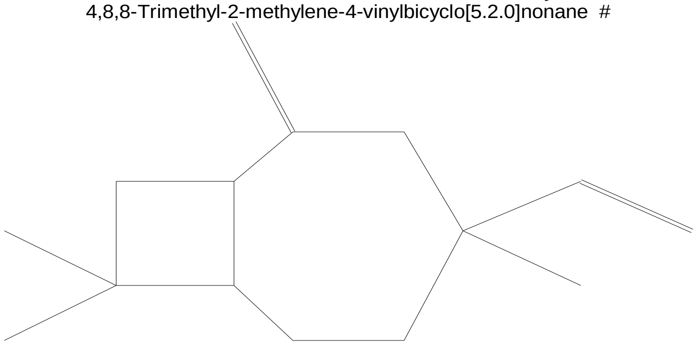

1,4-Methanocycloocta[d]pyridazine, 1,4,4a,5,6,9,10,10a-octahydro-11,11-dimethyl-, (1à,4à,4aà,10aà)-
Formula C13H20N2, MW 204, CAS# NA, Entry# 83802
$:28ILGXCBRAVUOOIM-ARJAWSKDSA-N

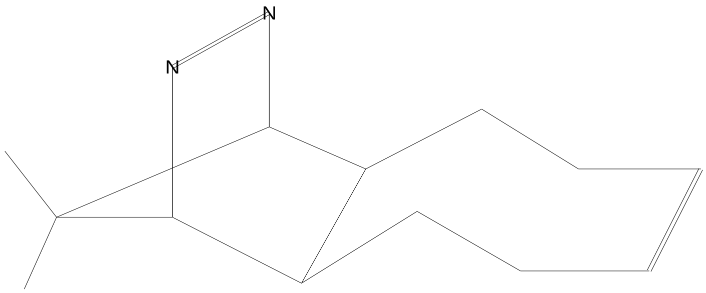

Longifolene
Formula C15H24, MW 204, CAS# 475-20-7, Entry# 13688
1,4-Methanoazulene, decahydro-4,8,8-trimethyl-9-methylene-, [1S-(1à,3aá,4à,8aá)]-

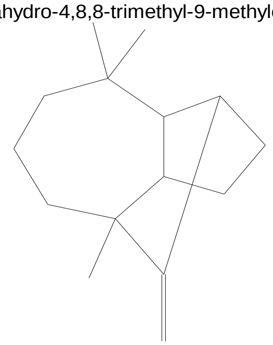

ck3 #5137 RT: 17.97 AV: 1 AV: 5 SB: 12 5130-5135 5139-5144 NL: 2.32E5
T: + c EI Full ms [33.00-450.00]

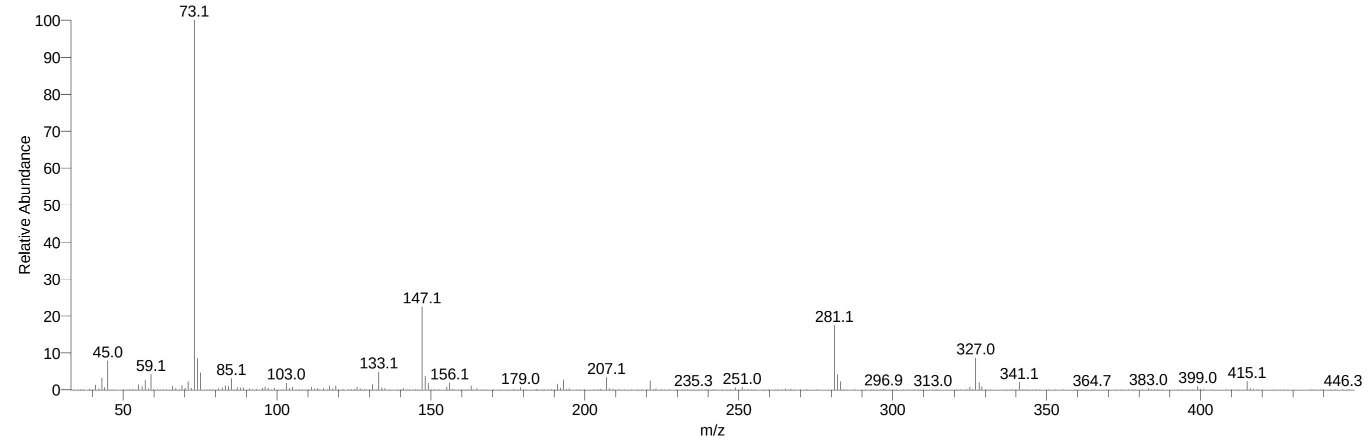

SI	Compound Name	RT	Cas #	Probability
673	3-Isopropoxy-1,1,1,7,7,7-hexamethyl-3,5,5-tris(trimethylsiloxy)tetrasiloxane	17.97	71579-69-6	39.22
663	3-Butoxy-1,1,1,7,7,7-hexamethyl-3,5,5-tris(trimethylsiloxy) tetrasiloxane	17.97	72439-84-0	27.68
627	Cycloheptasiloxane, tetradecamethyl-	17.97	107-50-6	6.84

Compound Structure

3-Isopropoxy-1,1,1,7,7,7-hexamethyl-3,5,5-tris(trimethylsiloxy)tetrasiloxane
Formula C18H52O7Si7, MW 576, CAS# 71579-69-6, Entry# 42479
1-Isopropoxy-3,3,3-trimethyl-1-[(trimethylsilyl)oxy]disiloxanyl tris(trimethylsilyl) orthosilicate #

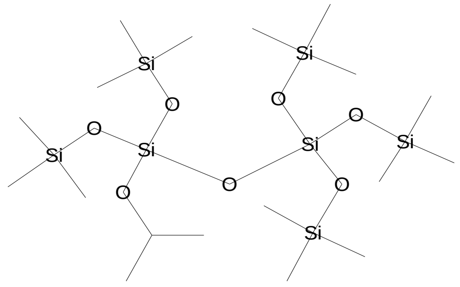

3-Butoxy-1,1,1,7,7,7-hexamethyl-3,5,5-tris(trimethylsiloxy)tetrasiloxane
Formula C19H54O7Si7, MW 590, CAS# 72439-84-0, Entry# 42504
1-Butoxy-3,3,3-trimethyl-1-[(trimethylsilyl)oxy]disiloxanyl tris(trimethylsilyl) orthosilicate #

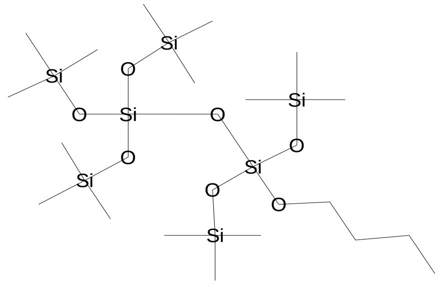

Cycloheptasiloxane, tetradecamethyl-
Formula C14H42O7Si7, MW 518, CAS# 107-50-6, Entry# 43769
2,2,4,4,6,6,8,8,10,10,12,12,14,14-Tetradecamethylcycloheptasiloxane #

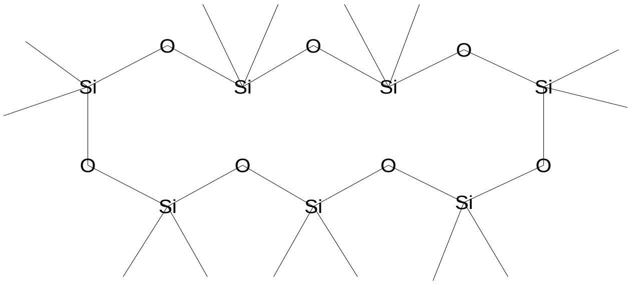

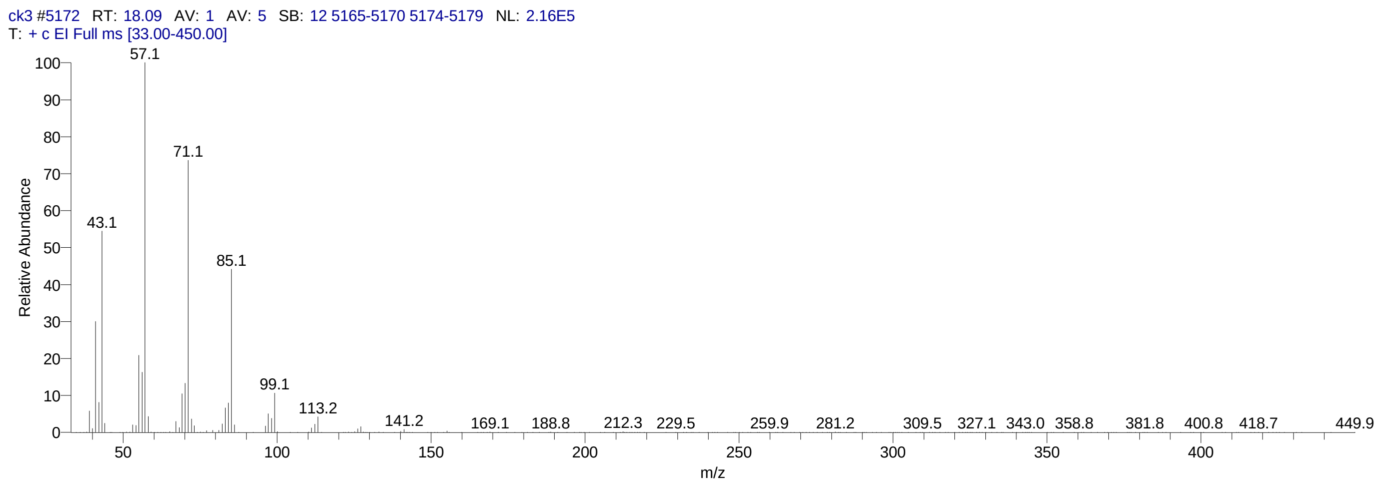

SI	Compound Name	RT	Cas #	Probability
885	Hexadecane	18.09	544-76-3	8.29
874	Hexadecane	18.09	544-76-3	8.29
874	Hexadecane	18.09	544-76-3	8.29

Compound Structure

Hexadecane
Formula C16H34, MW 226, CAS# 544-76-3, Entry# 6166
n-Cetane

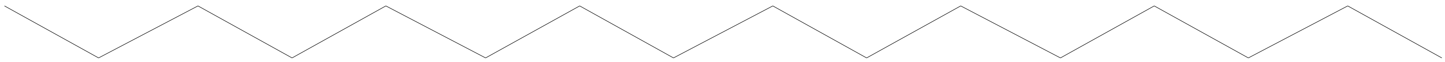

Hexadecane
Formula C16H34, MW 226, CAS# 544-76-3, Entry# 6165
n-Cetane

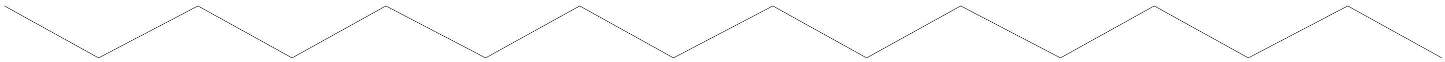

Hexadecane
Formula C16H34, MW 226, CAS# 544-76-3, Entry# 6168
n-Cetane

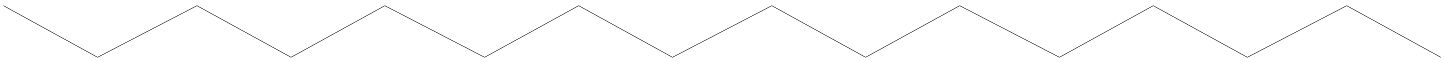

ck3 #5405 RT: 18.88 AV: 1 AV: 5 SB: 12 5398-5403 5407-5412 NL: 1.45E4
T: + c EI Full ms [33.00-450.00]

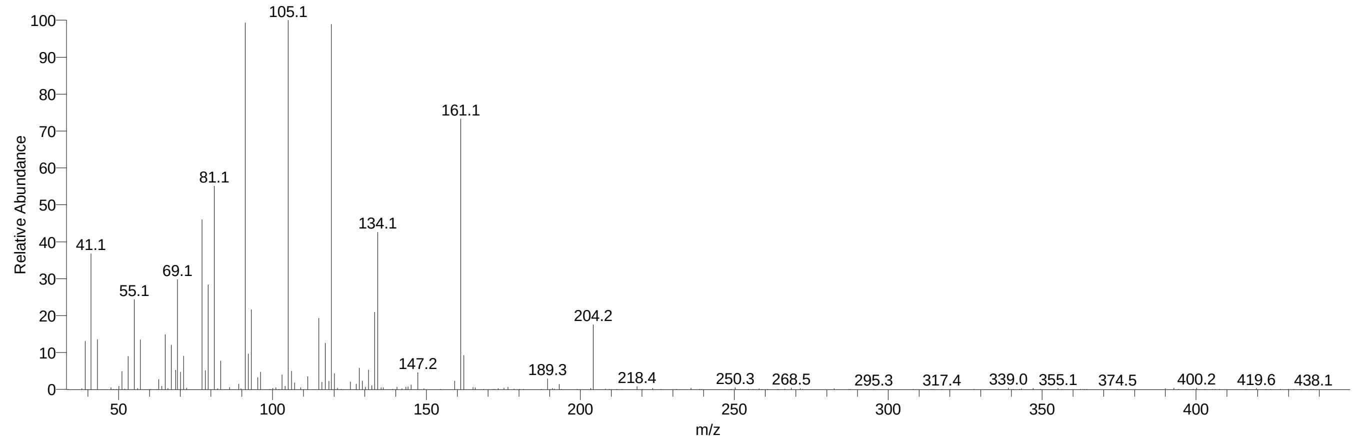

SI	Compound Name	RT	Cas #	Probability
756	cis-muurola-3,5-diene	18.88	NA	14.69
753	1-Isopropyl-4,7-dimethyl-1,2,3,5,6,8a-hexahydronaphthalene	18.88	16729-01-4	12.98
740	Naphthalene, 1,2,3,5,6,8a-hexahydro-4,7-dimethyl-1-(1-methylethyl)-, (1S-cis)-	18.88	483-76-1	8.38

Compound Structure

cis-muurola-3,5-diene
Formula C15H24, MW 204, CAS# NA, Entry# 150496
$:28JOCWPECWTZZSSX-UHFFFAOYSA-N

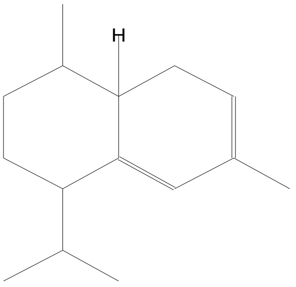

1-Isopropyl-4,7-dimethyl-1,2,3,5,6,8a-hexahydronaphthalene
Formula C15H24, MW 204, CAS# 16729-01-4, Entry# 150583
Cadina-1(10),4-diene

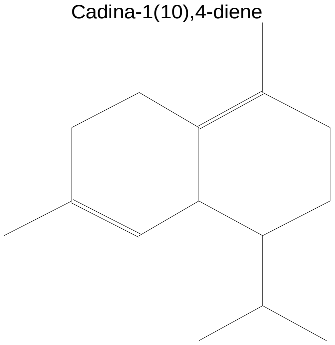

Naphthalene, 1,2,3,5,6,8a-hexahydro-4,7-dimethyl-1-(1-methylethyl)-, (1S-cis)-
Formula $C_{15}H_{24}$, MW 204, CAS# 483-76-1, Entry# 24907
Cadina-1(10),4-diene

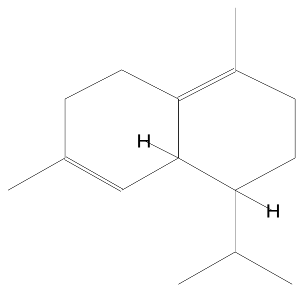

ck3 #5848 RT: 20.39 AV: 1 AV: 5 SB: 12 5841-5846 5850-5855 NL: 3.93E5
T: + c EI Full ms [33.00-450.00]

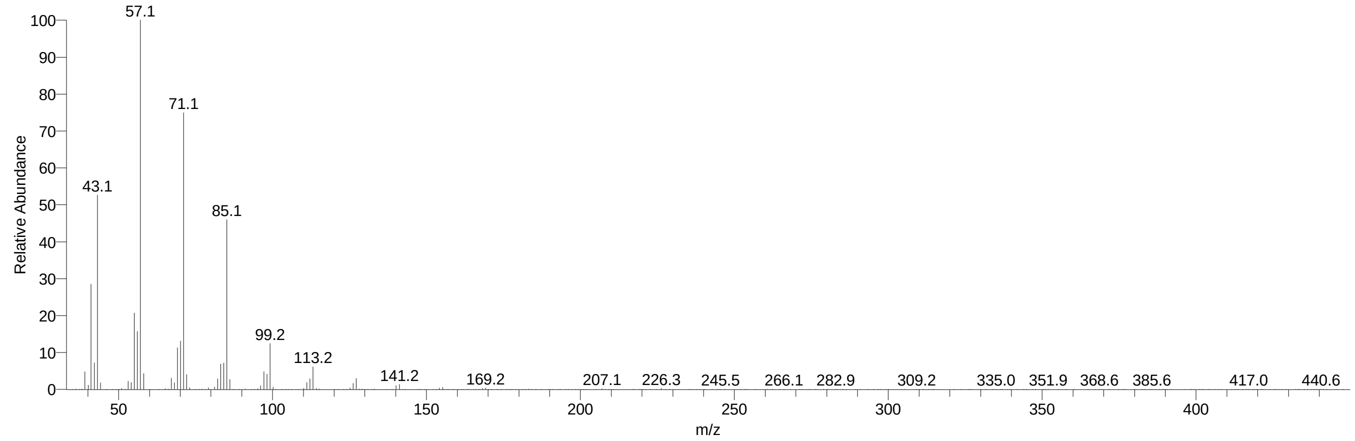

SI	Compound Name	RT	Cas #	Probability
914	Hexadecane	20.39	544-76-3	11.72
912	Nonadecane	20.39	629-92-5	10.81
901	Hexadecane	20.39	544-76-3	11.72

Compound Structure

Hexadecane
Formula C16H34, MW 226, CAS# 544-76-3, Entry# 6168
n-Cetane

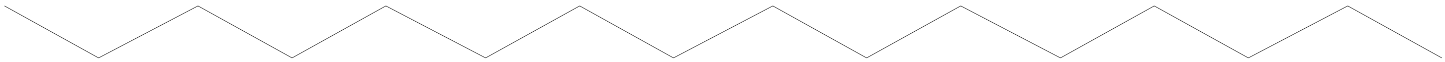

Nonadecane
Formula C19H40, MW 268, CAS# 629-92-5, Entry# 6141
n-Nonadecane

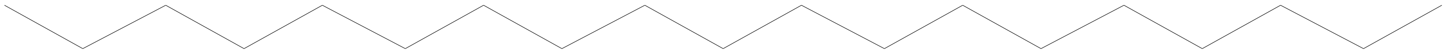

Hexadecane
Formula $C_{16}H_{34}$, MW 226, CAS# 544-76-3, Entry# 6165
n-Cetane

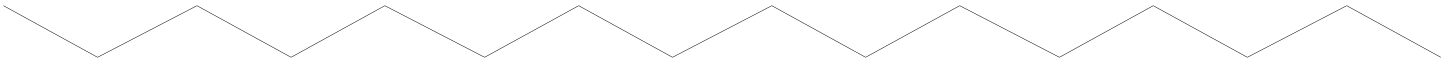

ck3 #6162 RT: 21.45 AV: 1 AV: 5 SB: 12 6155-6160 6164-6169 NL: 1.93E4
T: + c EI Full ms [33.00-450.00]

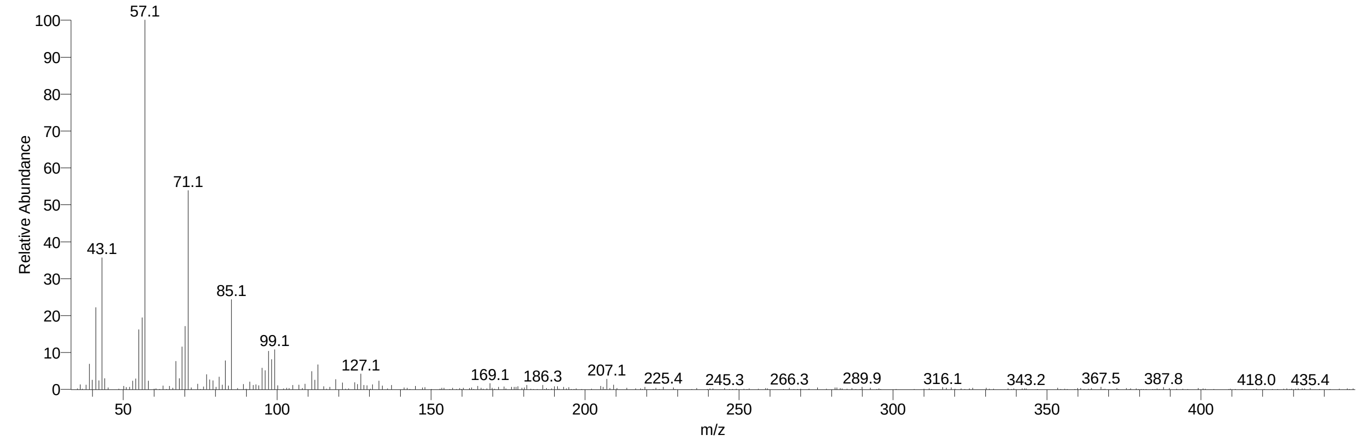

SI	Compound Name	RT	Cas #	Probability
674	Tetratetracontane	21.45	7098-22-8	7.56
666	Octadecane, 3-ethyl-5-(2-ethylbutyl)-	21.45	55282-12-7	5.64
658	Tetratetracontane	21.45	7098-22-8	7.56

Compound Structure

Tetratetracontane
Formula C44H90, MW 618, CAS# 7098-22-8, Entry# 6126
n-Tetratetracontane

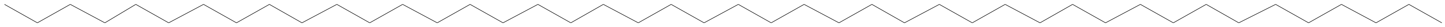

Octadecane, 3-ethyl-5-(2-ethylbutyl)-
Formula C26H54, MW 366, CAS# 55282-12-7, Entry# 7878
3-Ethyl-5-(2'-ethylbutyl)octadecane

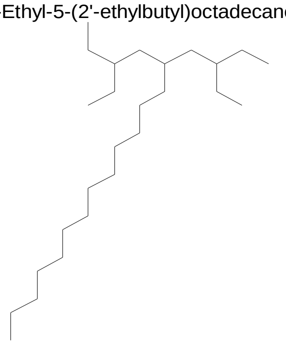

Tetratetracontane
Formula $C_{44}H_{90}$, MW 618, CAS# 7098-22-8, Entry# 6125
n-Tetratetracontane

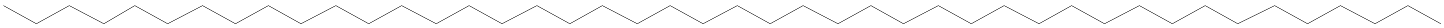

ck3 #6256 RT: 21.77 AV: 1 AV: 5 SB: 12 6249-6254 6258-6263 NL: 7.86E4
T: + c EI Full ms [33.00-450.00]

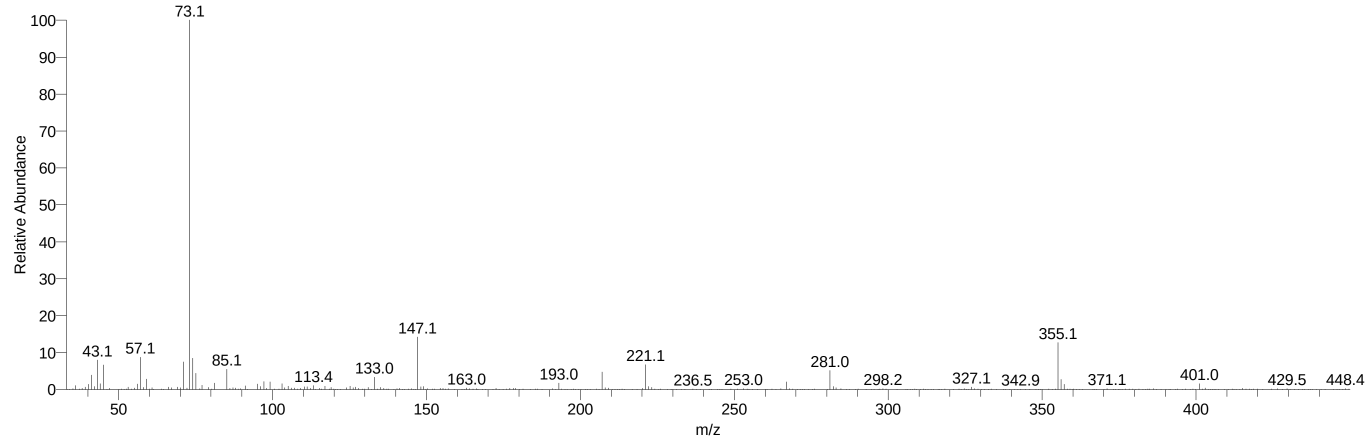

SI	Compound Name	RT	Cas #	Probability
625	Heptasiloxane, 1,1,3,3,5,5,7,7,9,9,11,11,13,13-tetradecamethyl-	21.77	19095-23-9	17.45
612	Octasiloxane, 1,1,3,3,5,5,7,7,9,9,11,11,13,13,15,15-hexadecamethyl-	21.77	19095-24-0	11.27
607	2,5-Dihydroxybenzoic acid, 3TMS derivative	21.77	3618-20-0	9.08

Compound Structure

Heptasiloxane, 1,1,3,3,5,5,7,7,9,9,11,11,13,13-tetradecamethyl-
Formula C14H44O6Si7, MW 504, CAS# 19095-23-9, Entry# 43295
1,1,3,3,5,5,7,7,9,9,11,11,13,13-Tetradecamethylheptasiloxane #

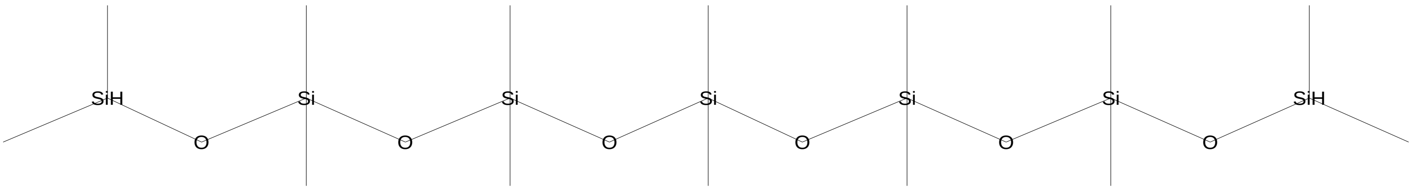

Octasiloxane, 1,1,3,3,5,5,7,7,9,9,11,11,13,13,15,15-hexadecamethyl-
Formula C16H50O7Si8, MW 578, CAS# 19095-24-0, Entry# 43294
1,1,3,3,5,5,7,7,9,9,11,11,13,13,15,15-Hexadecamethyloctasiloxane #

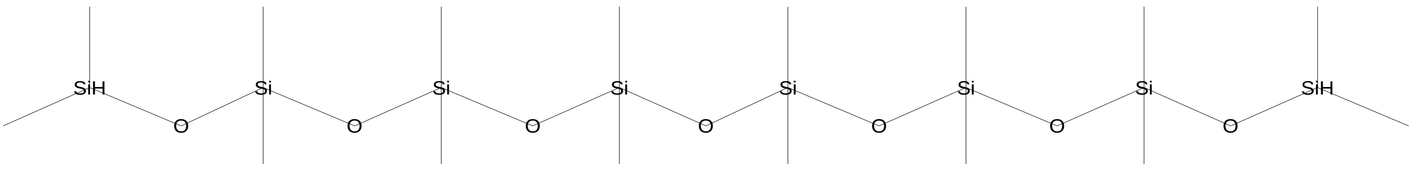

2,5-Dihydroxybenzoic acid, 3TMS derivative
Formula C16H30O4Si3, MW 370, CAS# 3618-20-0, Entry# 10280
Benzoic acid, 2,5-bis(trimethylsiloxy)-, trimethylsilyl ester

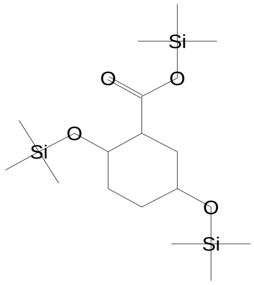

ck3 #6460 RT: 22.47 AV: 1 AV: 5 SB: 12 6453-6458 6462-6467 NL: 1.85E5
T: + c EI Full ms [33.00-450.00]

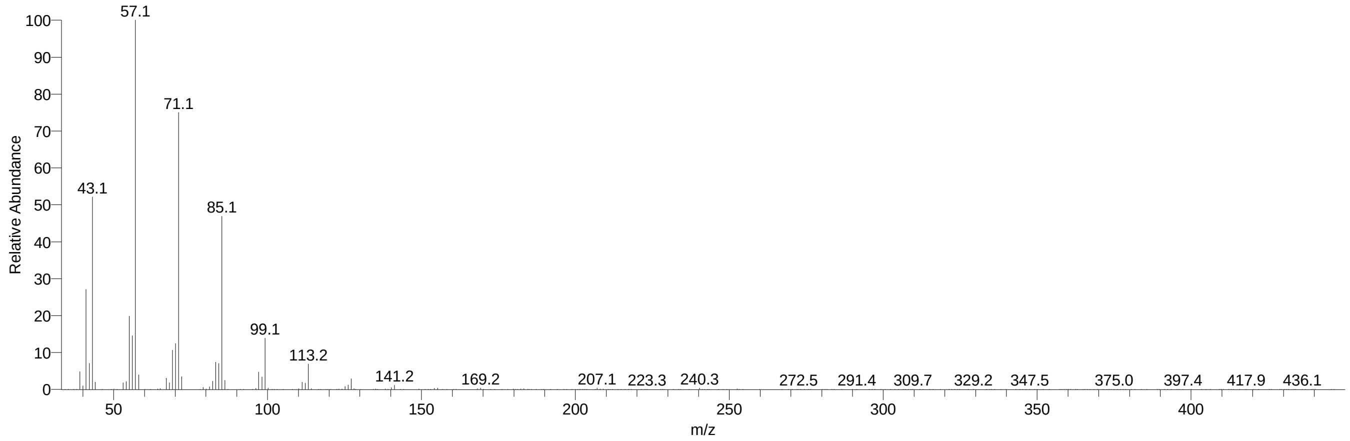

SI	Compound Name	RT	Cas #	Probability
899	Nonadecane	22.47	629-92-5	10.99
893	Eicosane	22.47	112-95-8	8.64
879	Hexadecane	22.47	544-76-3	5.41

Compound Structure

Nonadecane
Formula C19H40, MW 268, CAS# 629-92-5, Entry# 6141
n-Nonadecane

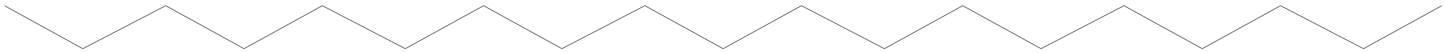

Eicosane
Formula C20H42, MW 282, CAS# 112-95-8, Entry# 6107
n-Eicosane

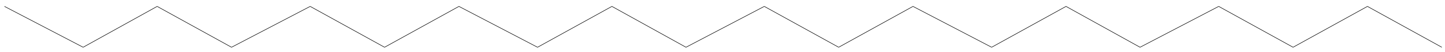

Hexadecane
Formula C16H34, MW 226, CAS# 544-76-3, Entry# 6168
n-Cetane

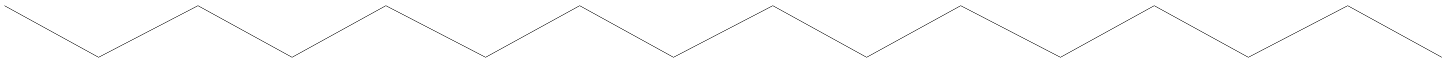

ck3 #6489 RT: 22.57 AV: 1 AV: 5 SB: 12 6482-6487 6491-6496 NL: 4.76E4
T: + c EI Full ms [33.00-450.00]

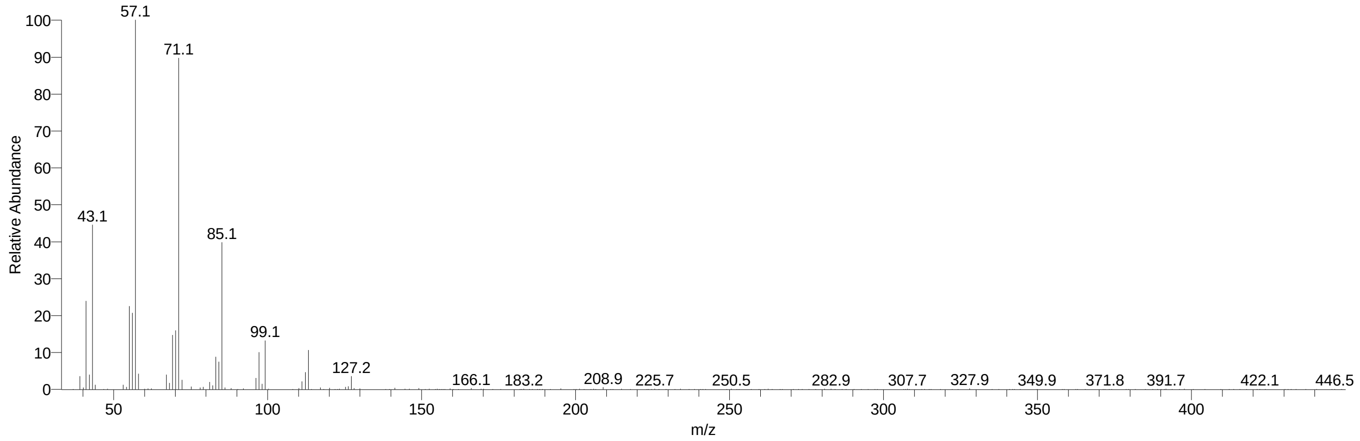

SI	Compound Name	RT	Cas #	Probability
855	Dodecane, 2,6,10-trimethyl-	22.57	3891-98-3	9.52
853	Dodecane, 2,6,10-trimethyl-	22.57	3891-98-3	9.52
845	Heptadecane, 2,6,10,15-tetramethyl-	22.57	54833-48-6	6.72

Compound Structure

Dodecane, 2,6,10-trimethyl-
Formula C15H32, MW 212, CAS# 3891-98-3, Entry# 6373
Farnesan

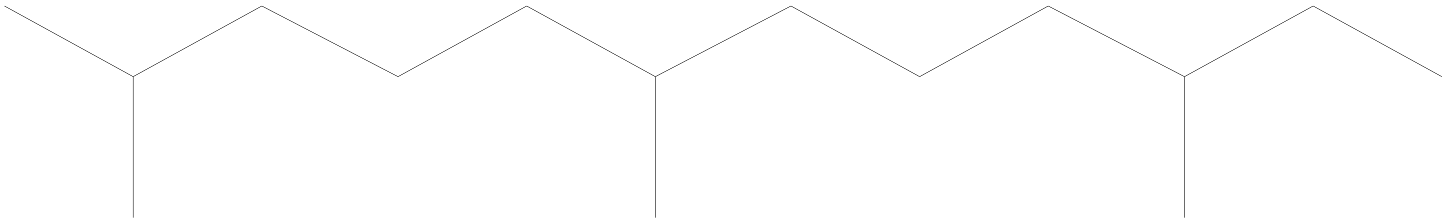

Dodecane, 2,6,10-trimethyl-
Formula C15H32, MW 212, CAS# 3891-98-3, Entry# 25322
Farnesan

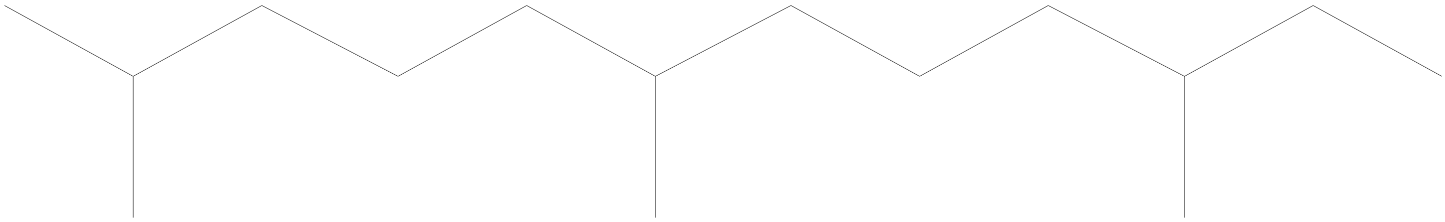

Heptadecane, 2,6,10,15-tetramethyl-
Formula C21H44, MW 296, CAS# 54833-48-6, Entry# 25344
2,6,10,15-Tetramethylheptadecane #

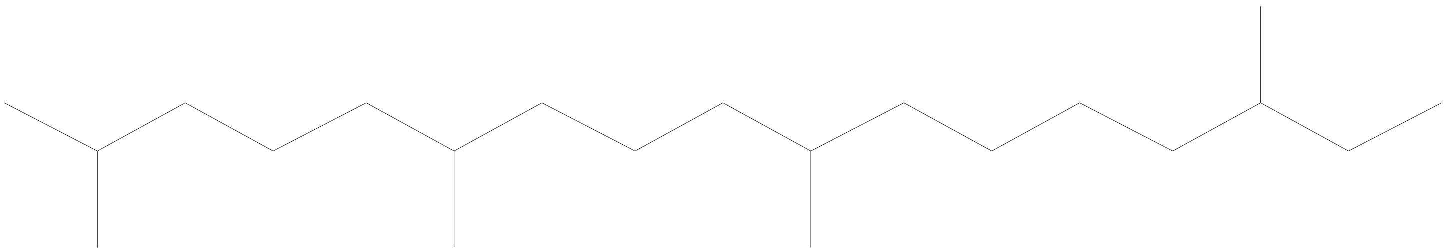

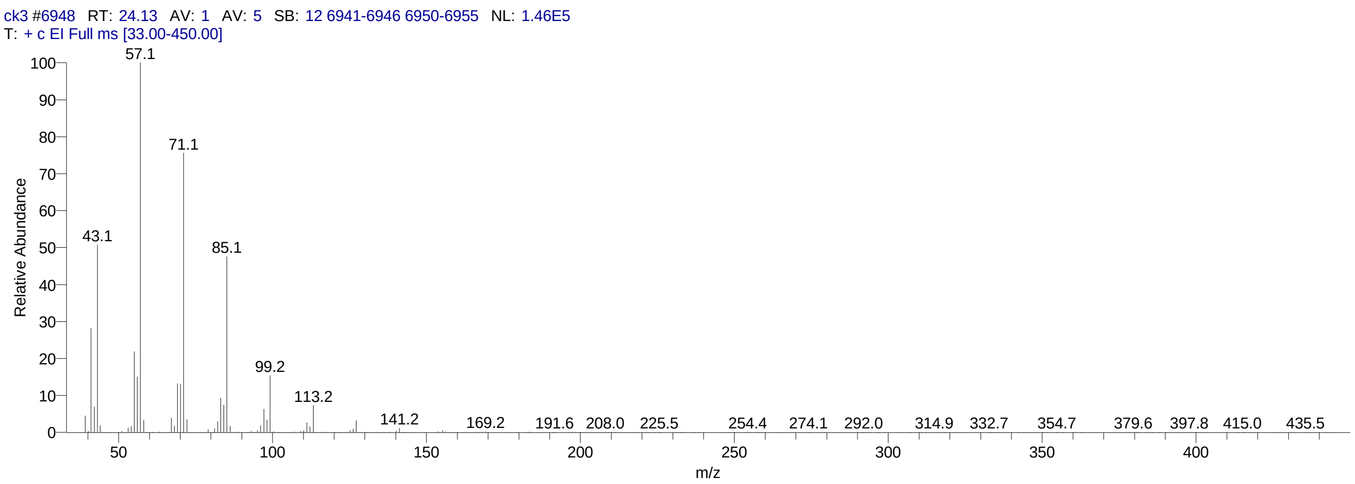

SI	Compound Name	RT	Cas #	Probability
873	Nonadecane	24.13	629-92-5	6.85
864	Heptadecane	24.13	629-78-7	4.97
862	1-Iodo-2-methylundecane	24.13	73105-67-6	4.59

Compound Structure

Nonadecane
Formula C19H40, MW 268, CAS# 629-92-5, Entry# 6141
n-Nonadecane

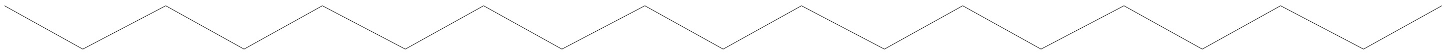

Heptadecane
Formula C17H36, MW 240, CAS# 629-78-7, Entry# 6087
n-Heptadecane

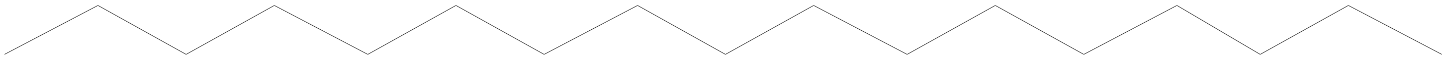

1-Iodo-2-methylundecane
Formula $C_{12}H_{25}I$, MW 296, CAS# 73105-67-6, Entry# 24226
$:28RTWBFGUVCAVDFO-UHFFFAOYSA-N

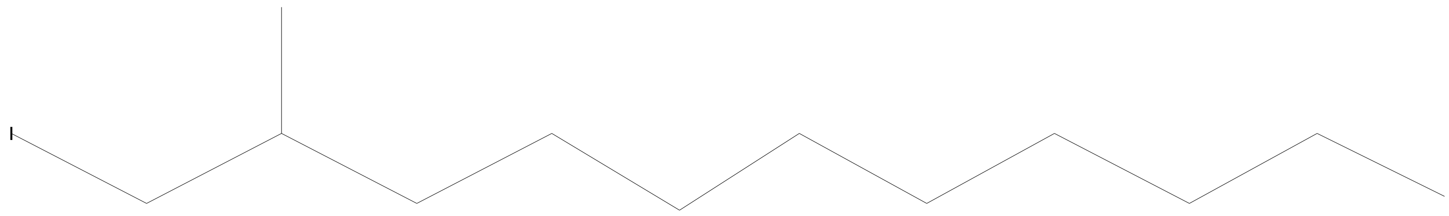

ck3 #7802 RT: 27.03 AV: 1 AV: 5 SB: 12 7795-7800 7804-7809 NL: 2.77E4
T: + c EI Full ms [33.00-450.00]

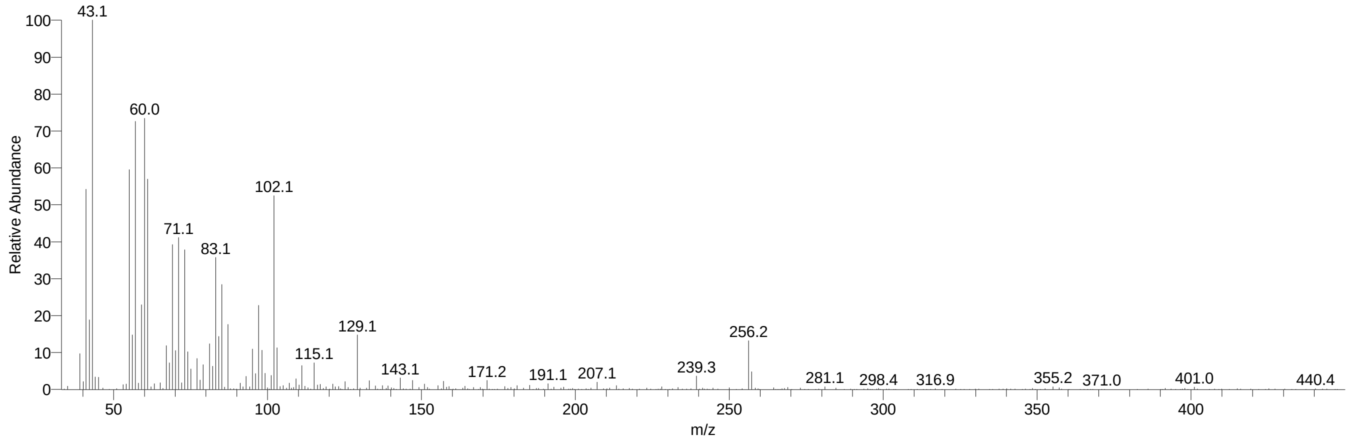

SI	Compound Name	RT	Cas #	Probability
731	Isopropyl palmitate	27.03	142-91-6	31.36
729	Isopropyl palmitate	27.03	142-91-6	31.36
713	n-Hexadecanoic acid	27.03	57-10-3	16.17

Compound Structure

Isopropyl palmitate
Formula C19H38O2, MW 298, CAS# 142-91-6, Entry# 15470
Hexadecanoic acid, 1-methylethyl ester

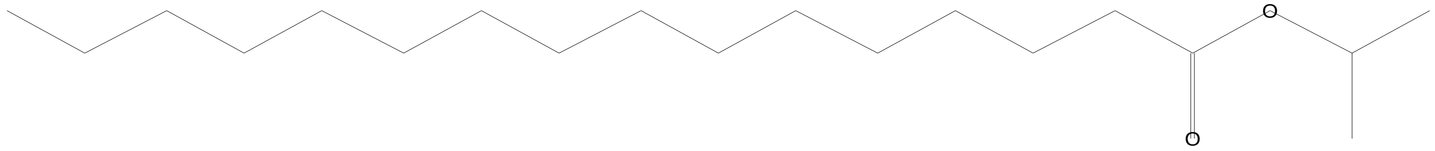

Isopropyl palmitate
Formula C19H38O2, MW 298, CAS# 142-91-6, Entry# 3083
Hexadecanoic acid, 1-methylethyl ester

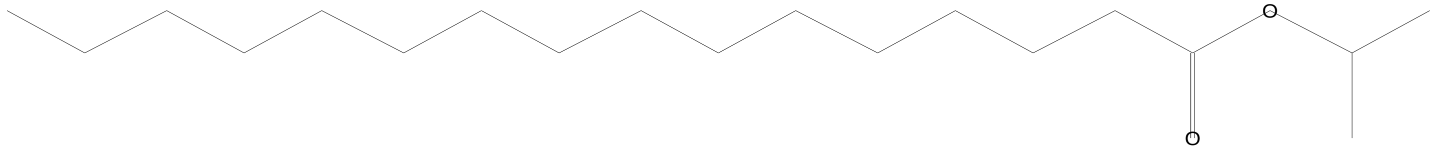

n-Hexadecanoic acid
Formula $C_{16}H_{32}O_2$, MW 256, CAS# 57-10-3, Entry# 154
Hexadecanoic acid

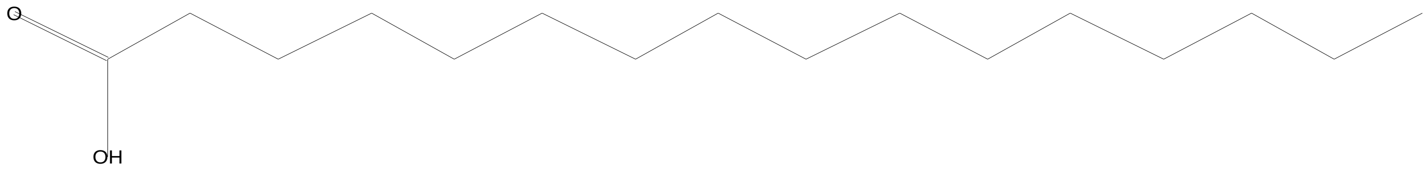

Supplement: DATA SHEET S1 — Report of GC-MS spectra from CK. [file Data_Sheet_1.PDF]
