# Supplementary material for: Antifungal Effects of Volatile Organic Compounds Produced by Rahnella aquatilis JZ-GX1 Against Colletotrichum gloeosporioides in Liriodendron chinense × tulipifera
Source: Front Microbiol. 2020 May 28;11:1114. doi: 10.3389/fmicb.2020.01114 (PMC7271530; doi:10.3389/fmicb.2020.01114)

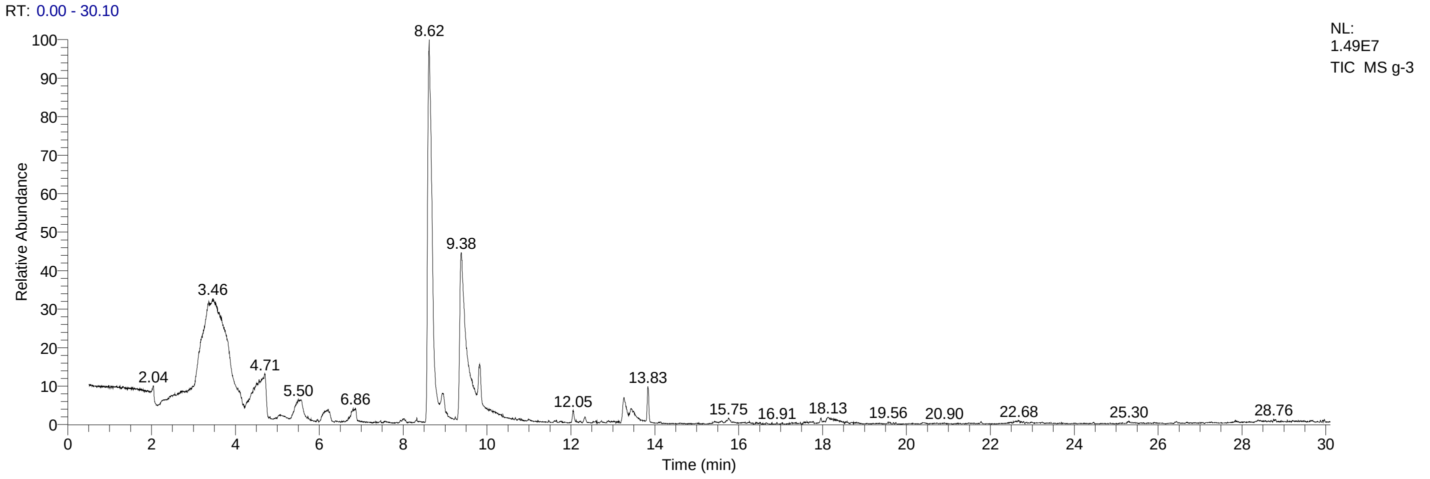

RT	Peak Area	Area %
2.04	2631998.39	0.99
2.26	468740.33	0.18
2.70	518521.93	0.20
3.36	36025372.44	13.56
3.46	58780055.57	22.12
4.11	841030.62	0.32
4.70	20044481.11	7.54
5.06	1205259.36	0.45
5.50	3135346.64	1.18
5.56	2762083.30	1.04
6.20	4295854.27	1.62
6.86	2695369.89	1.01
8.01	858695.85	0.32
8.32	463785.16	0.17
8.62	78646486.49	29.60
8.95	3026405.24	1.14
9.38	29357551.34	11.05
9.83	5230195.99	1.97
12.05	1389382.66	0.52
12.34	755156.83	0.28
13.26	4059895.89	1.53
13.44	1393507.39	0.52
13.84	3550497.85	1.34
15.43	367605.75	0.14
15.76	720276.84	0.27
17.77	415404.04	0.16
17.96	400104.43	0.15
18.13	772095.01	0.29
22.68	569836.43	0.21
25.30	352416.87	0.13

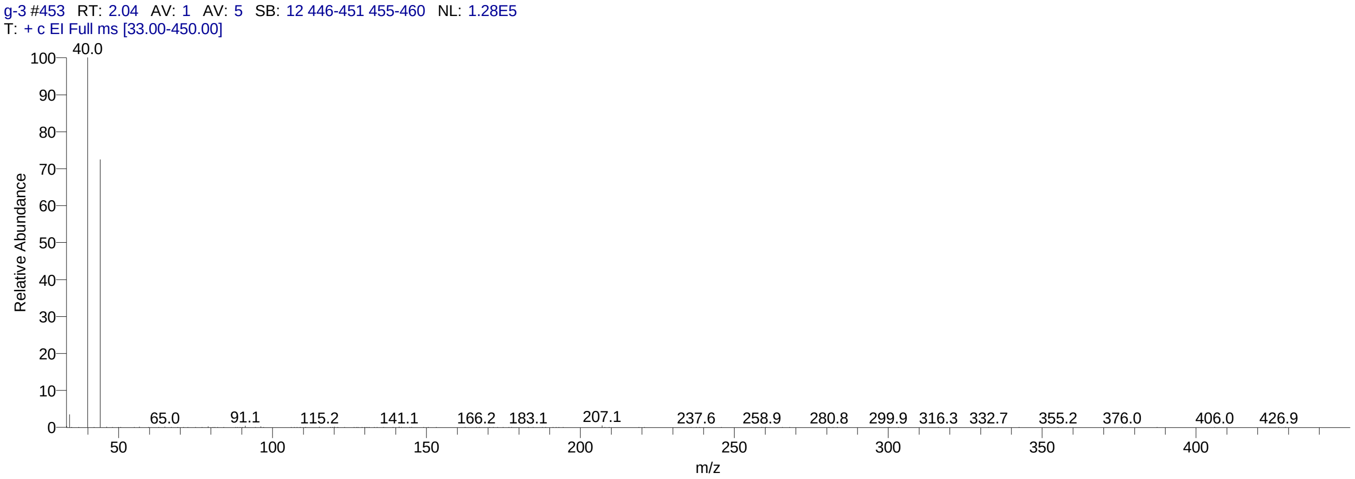

SI	Compound Name	RT	Cas #	Probability
943	L-Ala-L-Ala-L-Ala	2.04	5874-90-8	21.12
943	L-Ala-L-Ala-L-Ala	2.04	5874-90-8	21.12
943	Sarcosine	2.04	107-97-1	21.12

Compound Structure

Sarcosine
Formula C3H7NO2, MW 89, CAS# 107-97-1, Entry# 130003
$:03[M+H]+

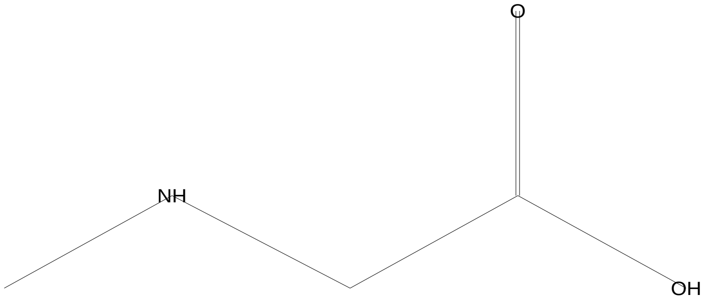

L-Ala-L-Ala-L-Ala
Formula C9H17N3O4, MW 231, CAS# 5874-90-8, Entry# 133078
$:03[M+H]+

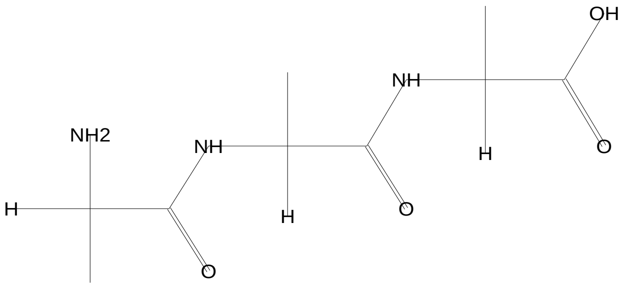

L-Ala-L-Ala-L-Ala
Formula C9H17N3O4, MW 231, CAS# 5874-90-8, Entry# 133079
$:03[M+H]+

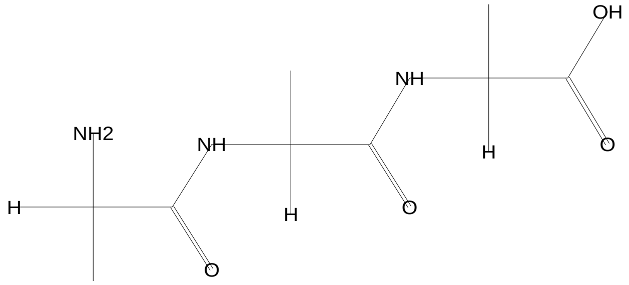

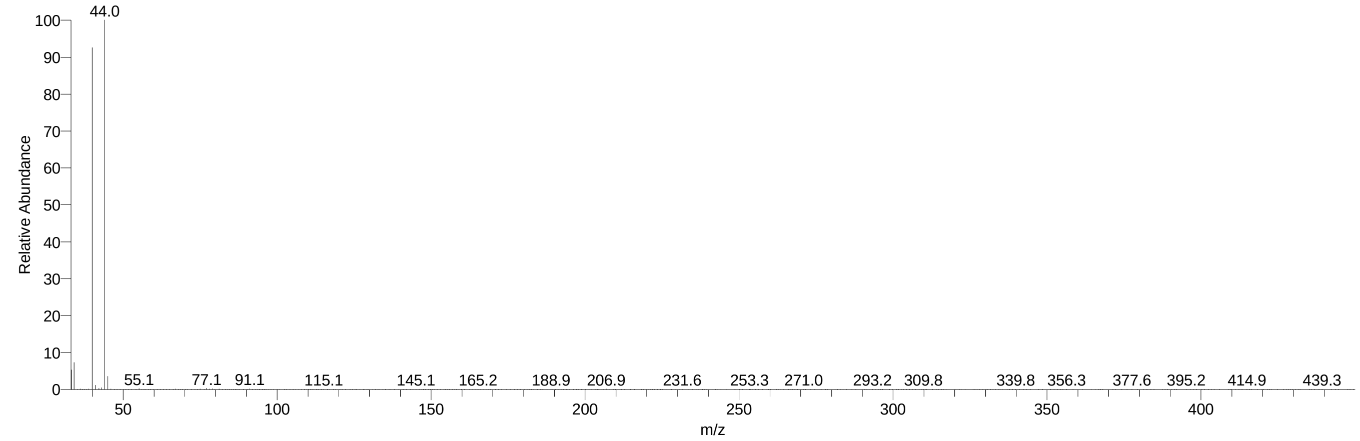

SI	Compound Name	RT	Cas #	Probability
972	Ethanolamine	2.26	141-43-5	33.60
951	L-Ala-L-Ala-L-Ala	2.26	5874-90-8	14.29
951	Sarcosine	2.26	107-97-1	14.29

Compound Structure

Ethanolamine
Formula C2H7NO, MW 61, CAS# 141-43-5, Entry# 122613
$:03[M+H]+

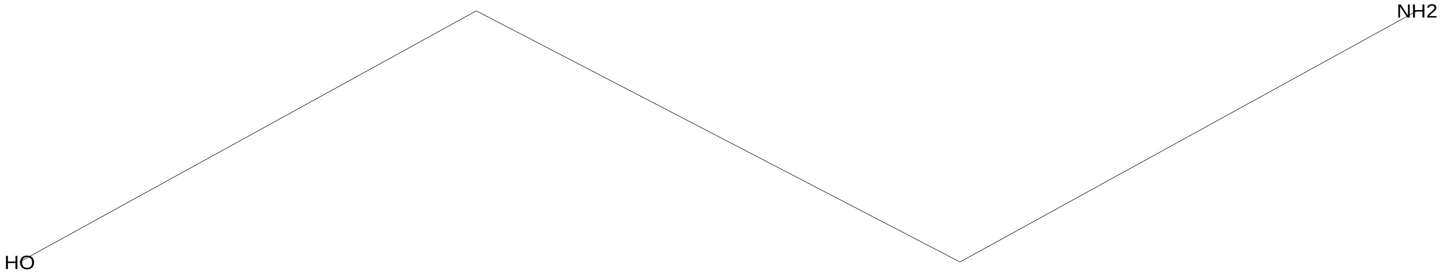

Sarcosine
Formula C3H7NO2, MW 89, CAS# 107-97-1, Entry# 130003
$:03[M+H]+

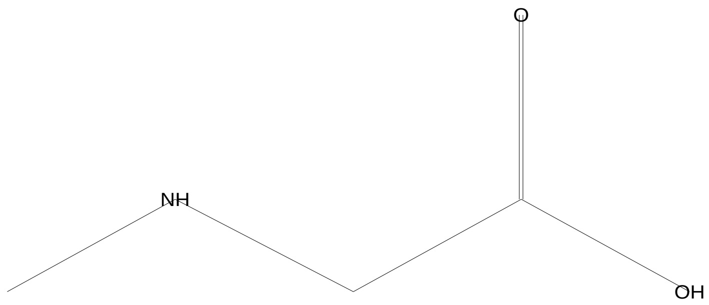

L-Ala-L-Ala-L-Ala
Formula C9H17N3O4, MW 231, CAS# 5874-90-8, Entry# 133078
$:03[M+H]+

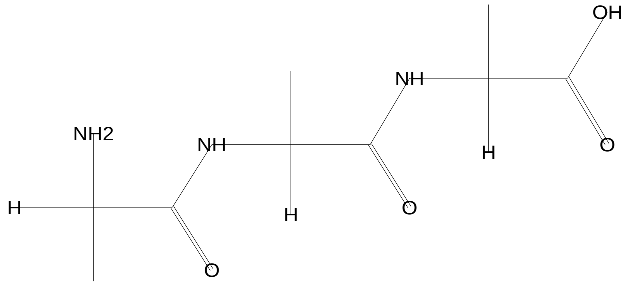

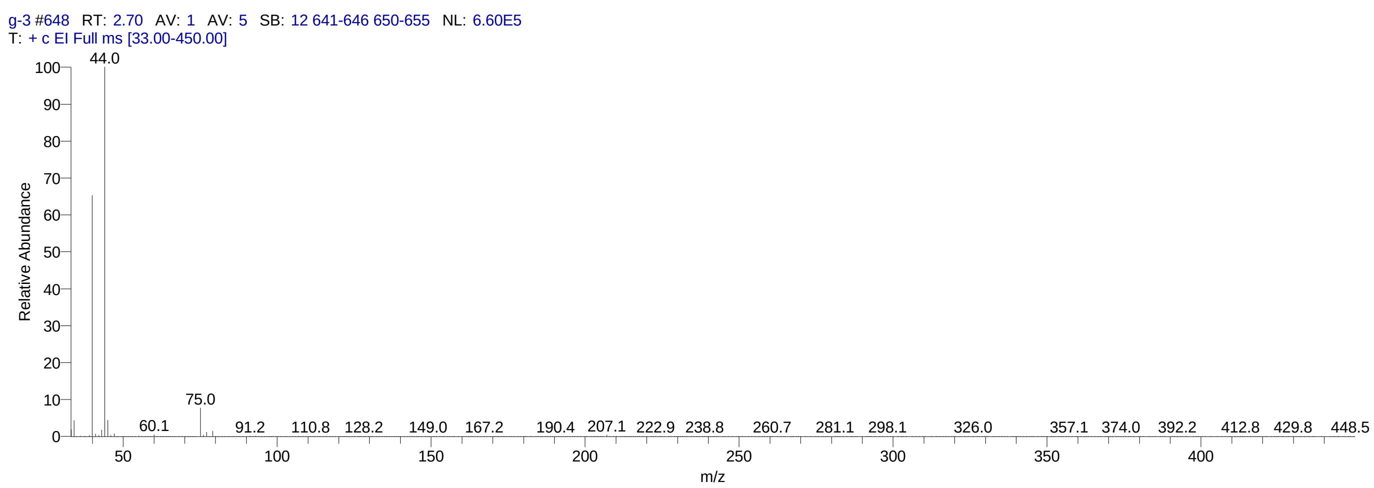

SI	Compound Name	RT	Cas #	Probability
868	Ethanolamine	2.70	141-43-5	57.96
791	L-Ala-L-Ala-L-Ala	2.70	5874-90-8	7.07
791	Sarcosine	2.70	107-97-1	7.07

Compound Structure

Ethanolamine
Formula C2H7NO, MW 61, CAS# 141-43-5, Entry# 122613
$:03[M+H]+

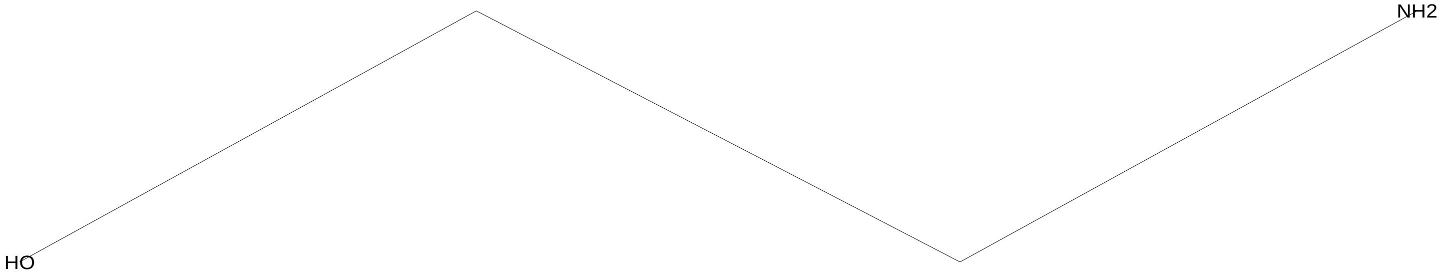

Sarcosine
Formula C3H7NO2, MW 89, CAS# 107-97-1, Entry# 130003
$:03[M+H]+

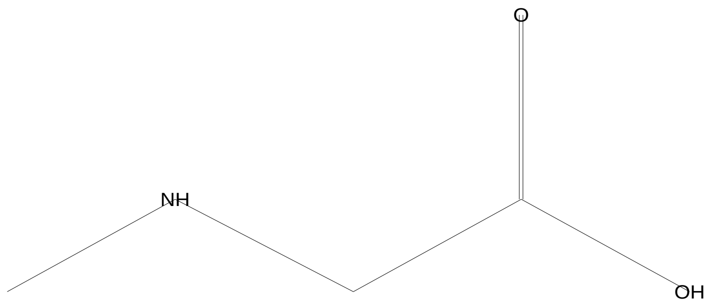

L-Ala-L-Ala-L-Ala
Formula C9H17N3O4, MW 231, CAS# 5874-90-8, Entry# 133078
$:03[M+H]+

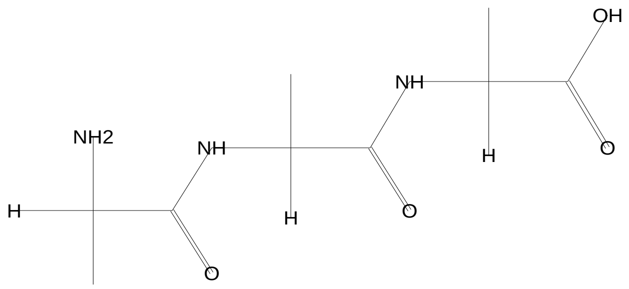

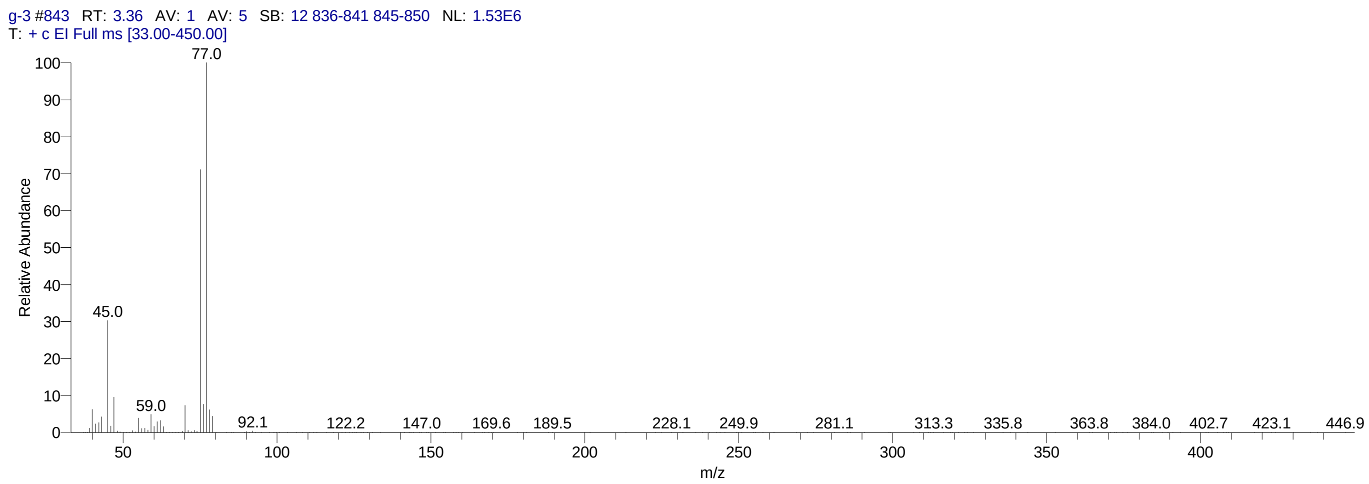

SI	Compound Name	RT	Cas #	Probability
742	2,2'-Dithiodiethanol	3.36	1892-29-1	43.81
730	Ethanol, 2-mercapto-	3.36	60-24-2	29.18
723	2,2'-Dithiodiethanol	3.36	1892-29-1	43.81

Compound Structure

2,2'-Dithiodiethanol
Formula C4H10O2S2, MW 154, CAS# 1892-29-1, Entry# 169797
$:03[M-H]-

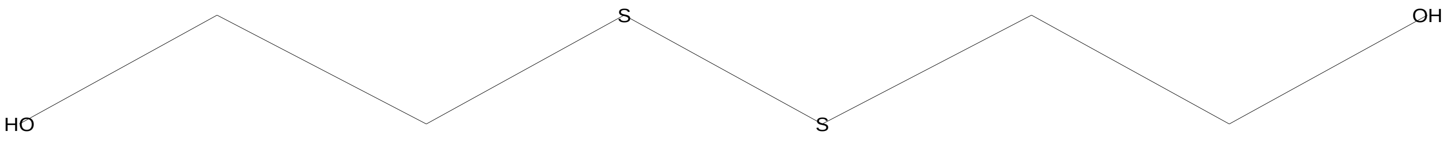

Ethanol, 2-mercapto-
Formula C2H6OS, MW 78, CAS# 60-24-2, Entry# 169779
$:03[M-H]-

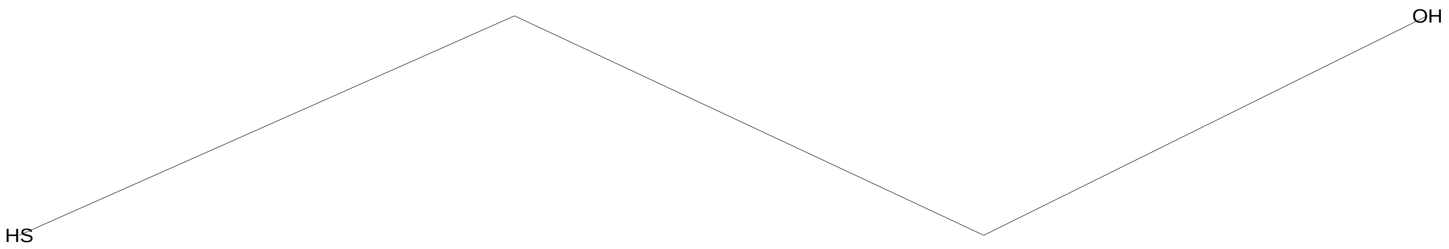

2,2'-Dithiodiethanol
Formula C4H10O2S2, MW 154, CAS# 1892-29-1, Entry# 169796
$:03[M-H]-

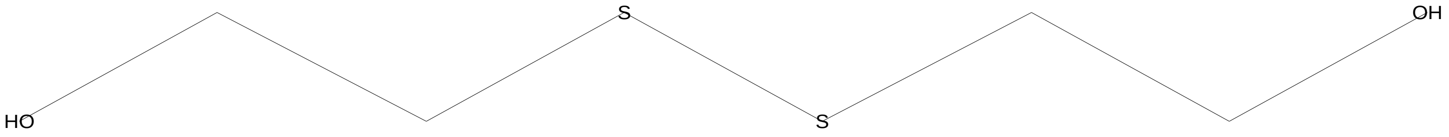

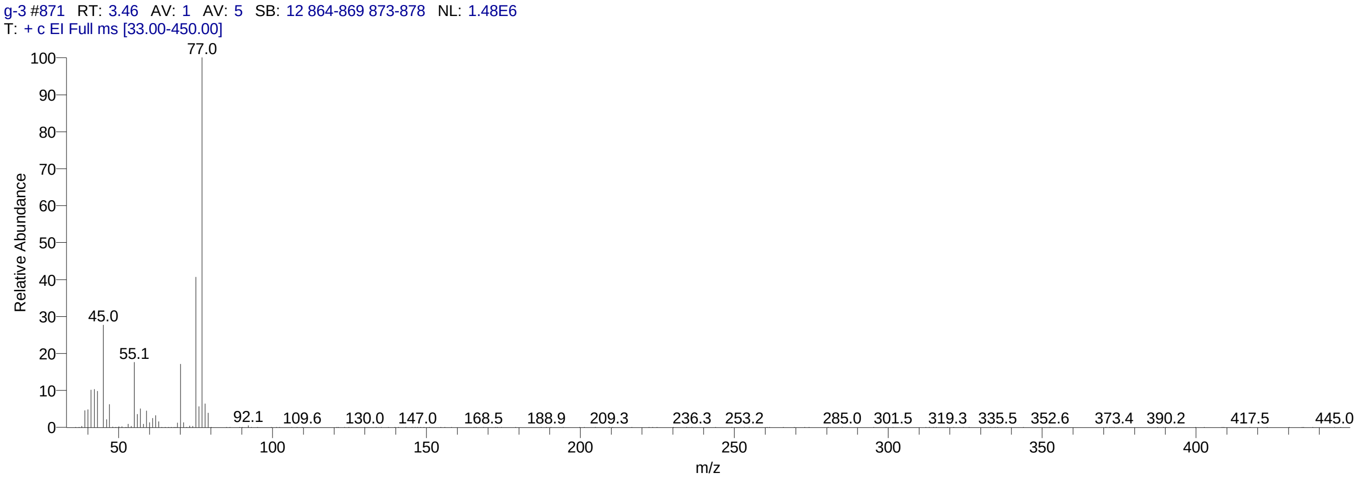

SI	Compound Name	RT	Cas #	Probability
744	Silanediol, dimethyl-	3.46	1066-42-8	62.75
725	1,2-Benzenedicarboxylic acid	3.46	88-99-3	30.47
670	Silanediol, dimethyl-	3.46	1066-42-8	62.75

Compound Structure

Silanediol, dimethyl-
Formula C2H8O2Si, MW 92, CAS# 1066-42-8, Entry# 46514
Dihydroxydimethylsilane

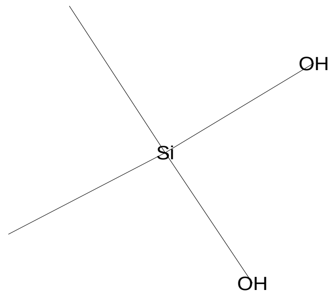

1,2-Benzenedicarboxylic acid
Formula C8H6O4, MW 166, CAS# 88-99-3, Entry# 177012
$:03[M-H]-

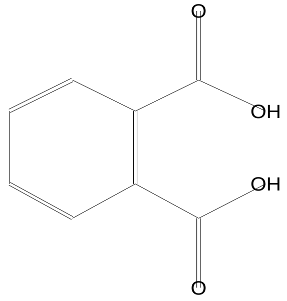

Silanediol, dimethyl-
Formula C2H8O2Si, MW 92, CAS# 1066-42-8, Entry# 10820
Dihydroxydimethylsilane

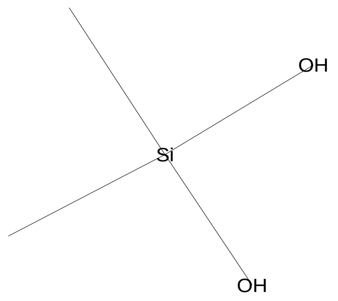

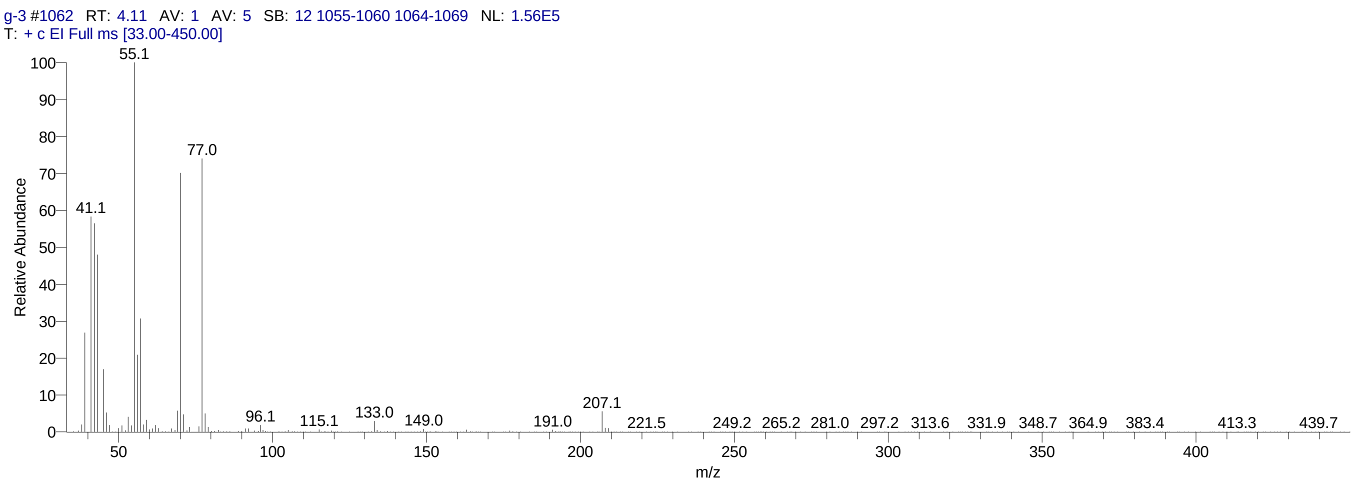

SI	Compound Name	RT	Cas #	Probability
735	1-Butanol, 3-methyl-	4.11	123-51-3	33.46
717	1-Butanol, 3-methyl-	4.11	123-51-3	33.46
717	1-Butanol, 3-methyl-	4.11	123-51-3	33.46

Compound Structure

1-Butanol, 3-methyl-
Formula C5H12O, MW 88, CAS# 123-51-3, Entry# 4806
Isopentyl alcohol

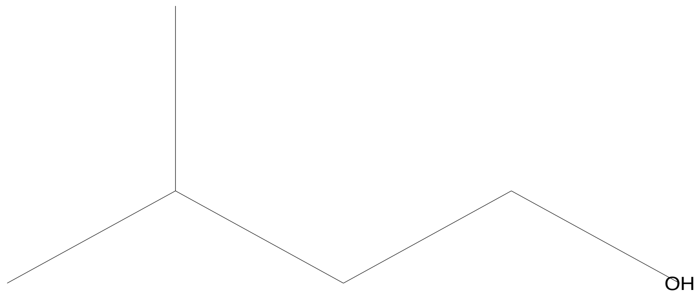

1-Butanol, 3-methyl-
Formula C5H12O, MW 88, CAS# 123-51-3, Entry# 1146
Isopentyl alcohol

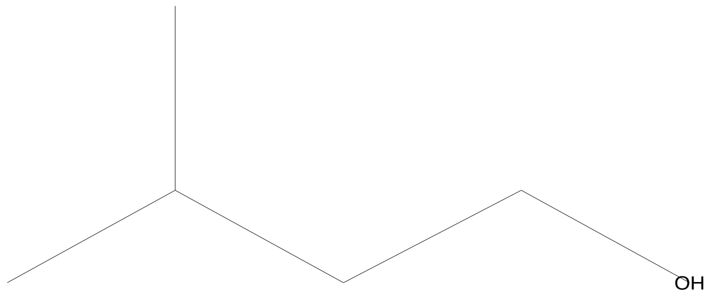

1-Butanol, 3-methyl-
Formula C5H12O, MW 88, CAS# 123-51-3, Entry# 19562
Isopentyl alcohol

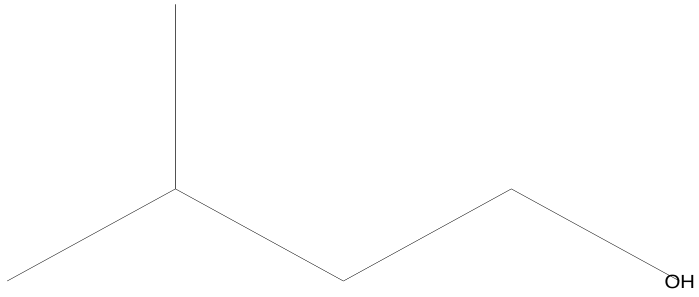

g-3 #1236 RT: 4.70 AV: 1 AV: 5 SB: 12 1229-1234 1238-1243 NL: 3.92E5
T: + c EI Full ms [33.00-450.00]

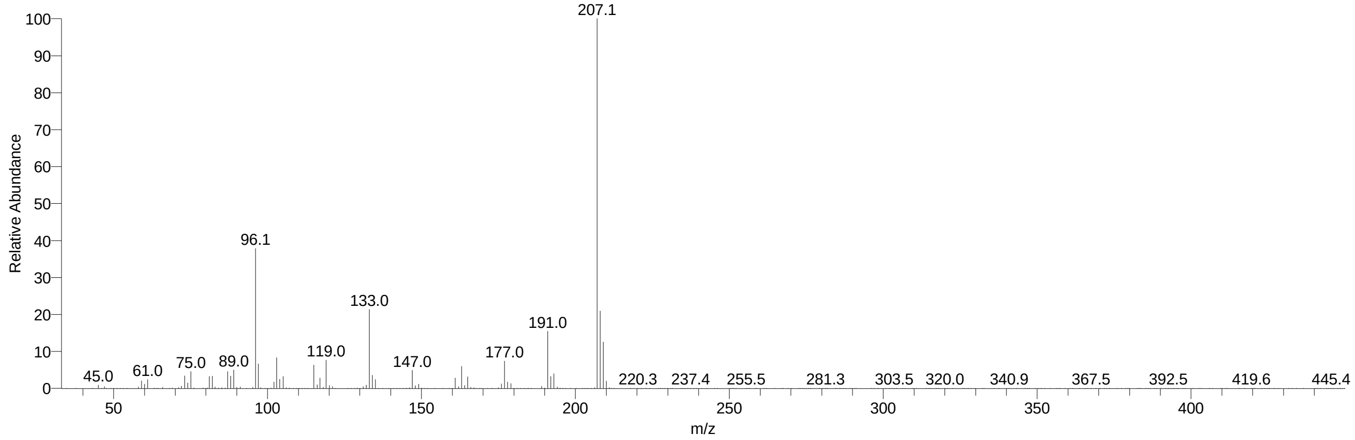

SI	Compound Name	RT	Cas #	Probability
900	Cyclotrisiloxane, hexamethyl-	4.70	541-05-9	91.19
868	Cyclotrisiloxane, hexamethyl-	4.70	541-05-9	91.19
785	Arsenous acid, tris(trimethylsilyl) ester	4.70	55429-29-3	5.47

Compound Structure

Cyclotrisiloxane, hexamethyl-
Formula C6H18O3Si3, MW 222, CAS# 541-05-9, Entry# 188945
Dimethylsiloxane cyclic trimer

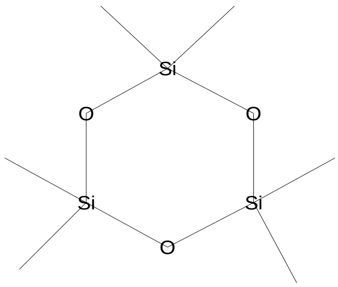

Cyclotrisiloxane, hexamethyl-
Formula C6H18O3Si3, MW 222, CAS# 541-05-9, Entry# 29125
Dimethylsiloxane cyclic trimer

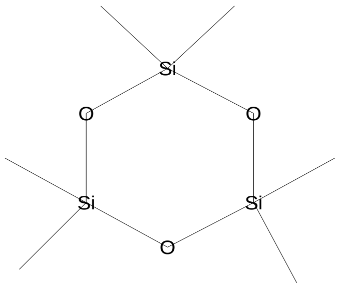

Arsenous acid, tris(trimethylsilyl) ester
Formula C9H27AsO3Si3, MW 342, CAS# 55429-29-3, Entry# 188948
$:28CWZNQWMMDOIFQC-UHFFFAOYSA-N

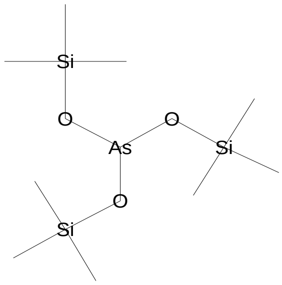

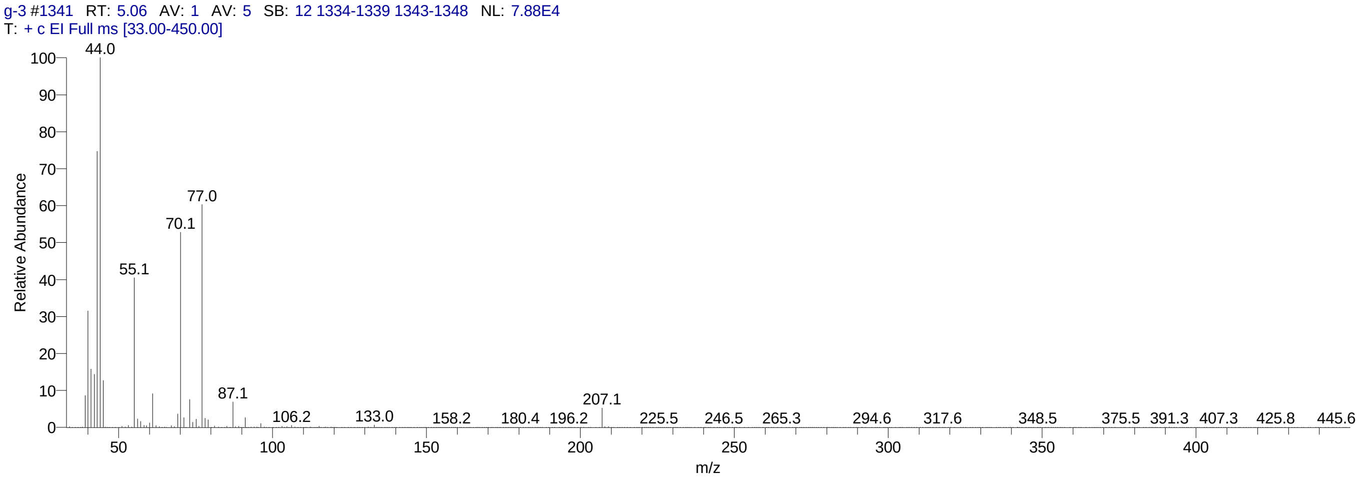

SI	Compound Name	RT	Cas #	Probability
637	Carbonic acid, methyl pentyl ester	5.06	NA	29.93
621	Butanal, 3-hydroxy-	5.06	107-89-1	17.24
602	1-Butanol, 3-methyl-, acetate	5.06	123-92-2	8.37

Compound Structure

Carbonic acid, methyl pentyl ester
Formula C7H14O3, MW 146, CAS# NA, Entry# 46767
$:28PAQGTCFSKWUKHW-UHFFFAOYSA-N

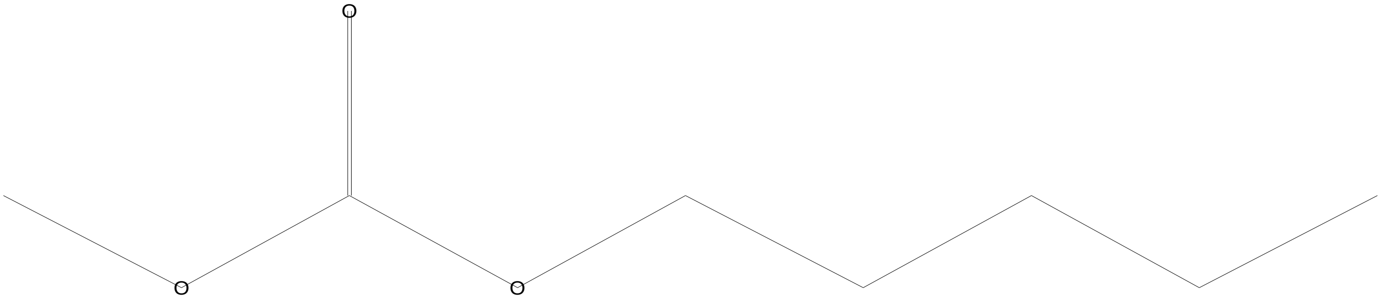

Butanal, 3-hydroxy-
Formula C4H8O2, MW 88, CAS# 107-89-1, Entry# 189
Butyraldehyde, 3-hydroxy-

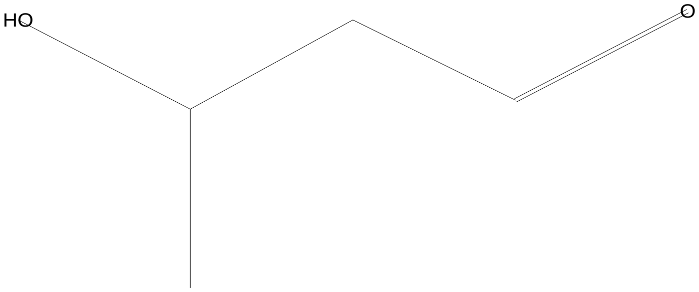

1-Butanol, 3-methyl-, acetate
Formula C7H14O2, MW 130, CAS# 123-92-2, Entry# 2564
Isopentyl alcohol, acetate

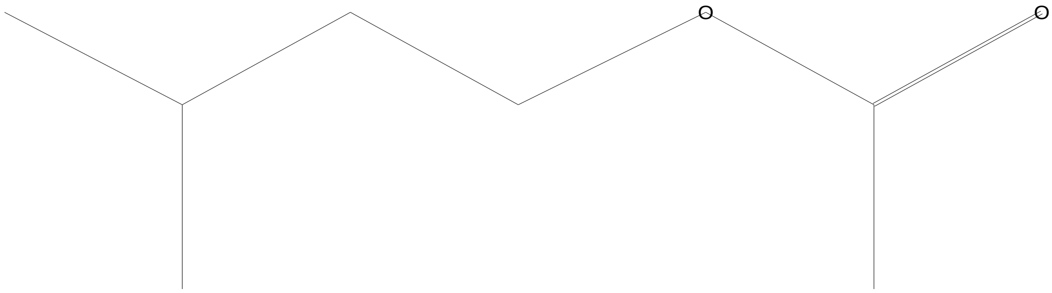

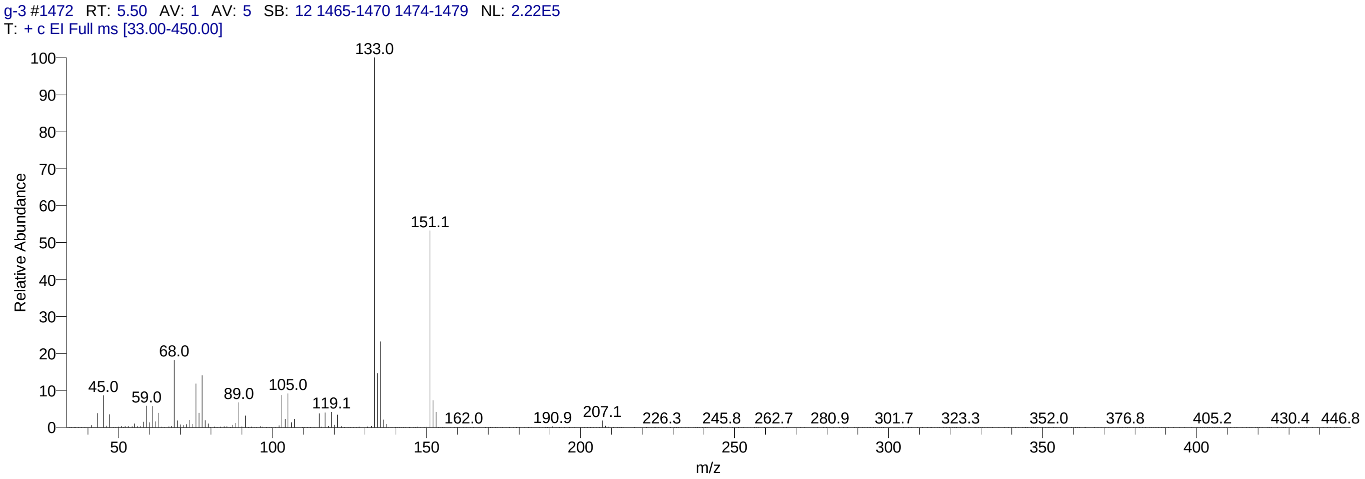

SI	Compound Name	RT	Cas #	Probability
798	Oxime-, methoxy-phenyl-_	5.50	NA	85.22
685	4-Ethylbenzoic acid, cyclopentyl ester	5.50	NA	5.24
660	4-Ethylbenzoic acid, 2-butyl ester	5.50	NA	1.60

Compound Structure

Oxime-, methoxy-phenyl-_
Formula C8H9NO2, MW 151, CAS# NA, Entry# 117767
Methyl N-hydroxybenzenecarboximidoate #

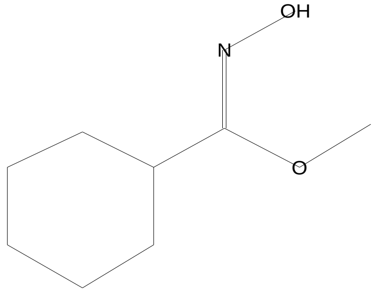

4-Ethylbenzoic acid, cyclopentyl ester
Formula C14H18O2, MW 218, CAS# NA, Entry# 117763
Cyclopentyl 4-ethylbenzoate #

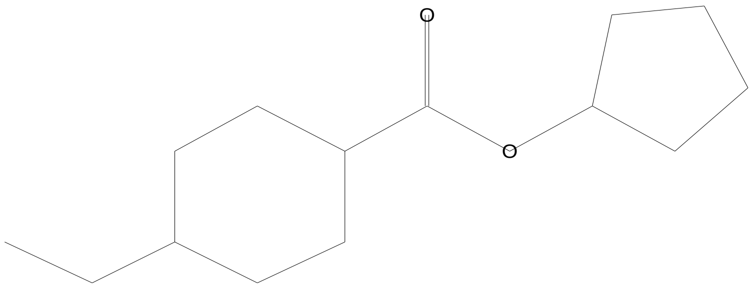

4-Ethylbenzoic acid, 2-butyl ester
Formula C13H18O2, MW 206, CAS# NA, Entry# 117762
Sec-butyl 4-ethylbenzoate #

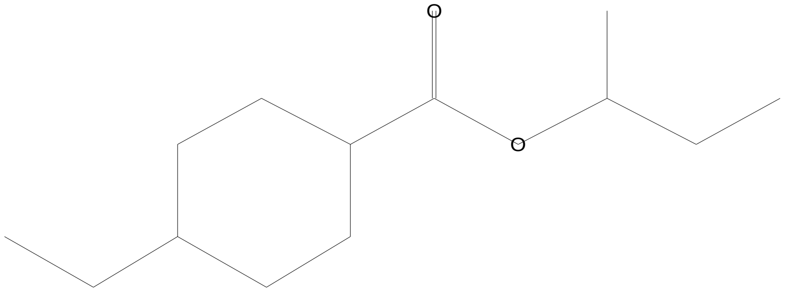

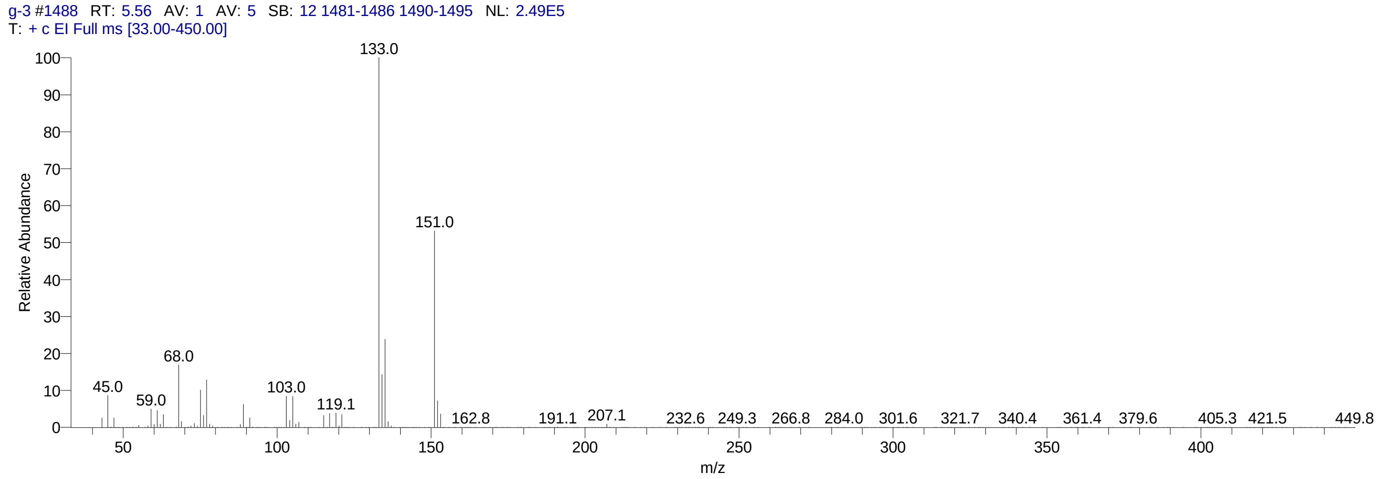

SI	Compound Name	RT	Cas #	Probability
826	Oxime-, methoxy-phenyl-_	5.56	NA	91.06
690	Esculetin	5.56	305-01-1	3.38
677	4-Ethylbenzoic acid, cyclopentyl ester	5.56	NA	2.18

Compound Structure

Oxime-, methoxy-phenyl-_
Formula C8H9NO2, MW 151, CAS# NA, Entry# 117767
Methyl N-hydroxybenzenecarboximidoate #

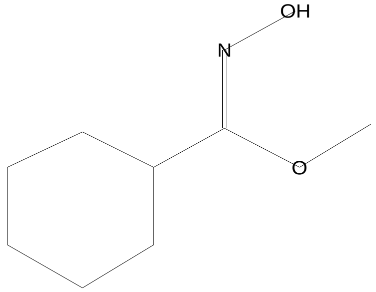

Esculetin
Formula C9H6O4, MW 178, CAS# 305-01-1, Entry# 105707
$:03[M+H]+

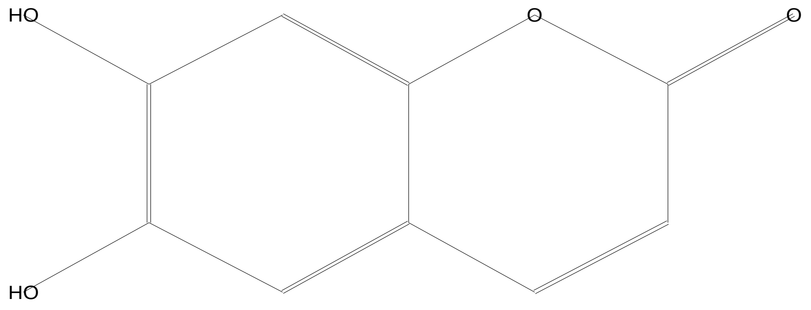

4-Ethylbenzoic acid, cyclopentyl ester
Formula C14H18O2, MW 218, CAS# NA, Entry# 117763
Cyclopentyl 4-ethylbenzoate #

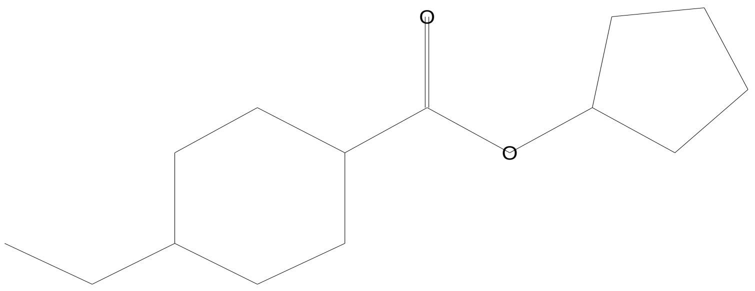

g-3 #1677 RT: 6.20 AV: 1 AV: 5 SB: 12 1670-1675 1679-1684 NL: 1.00E5
T: + c EI Full ms [33.00-450.00]

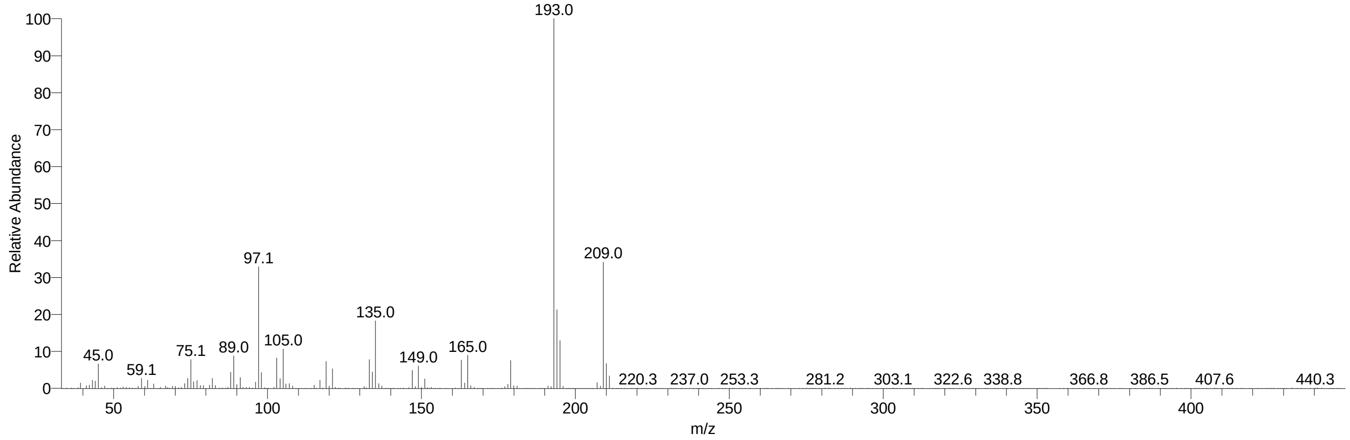

SI	Compound Name	RT	Cas #	Probability
628	Vanillin, TBDMS derivative	6.20	NA	9.86
628	Isovanillin, TBDMS derivative	6.20	NA	9.86
621	4-Isopropylphenol, TMS derivative	6.20	NA	7.55

Compound Structure

Isovanillin, TBDMS derivative
Formula C14H22O3Si, MW 266, CAS# NA, Entry# 180565
3-Hydroxy-4-methoxybenzaldehyde, tert-butyldimethylsilyl ether

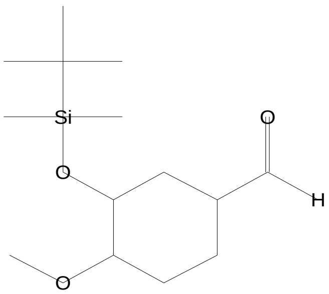

Vanillin, TBDMS derivative
Formula C14H22O3Si, MW 266, CAS# NA, Entry# 180564
Vanillin, tert-butyldimethylsilyl ether

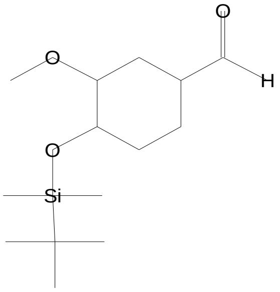

4-Isopropylphenol, TMS derivative
Formula C12H20OSi, MW 208, CAS# NA, Entry# 179480
4-Isopropylphenol, trimethylsilyl ether

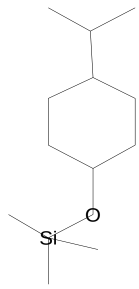

g-3 #1871 RT: 6.86 AV: 1 AV: 5 SB: 12 1864-1869 1873-1878 NL: 1.04E5
T: + c EI Full ms [33.00-450.00]

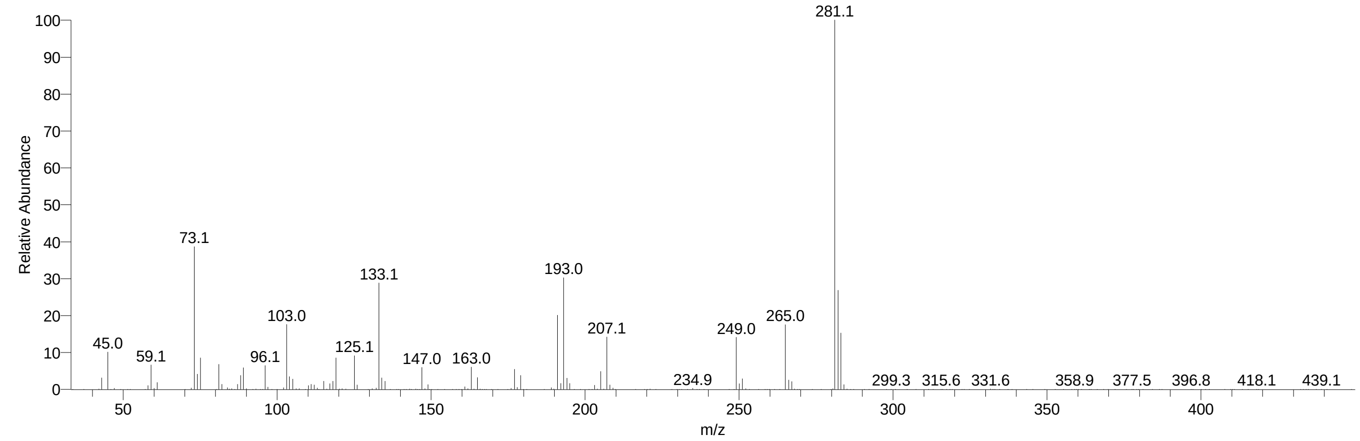

SI	Compound Name	RT	Cas #	Probability
814	Cyclotetrasiloxane, octamethyl-	6.86	556-67-2	36.67
814	Glafenin	6.86	3820-67-5	36.67
799	trisiloxane, 1,1,1,5,5,5-hexamethyl-3-[(trimethylsilyl)oxy]-	6.86	NA	22.22

Compound Structure

Glafenin
Formula C19H17ClN2O4, MW 372, CAS# 3820-67-5, Entry# 22947
$:30[M+H-H2O]+=>299.0

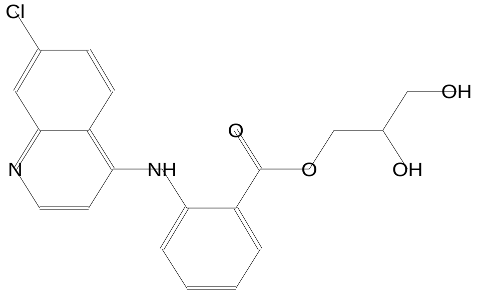

Cyclotetrasiloxane, octamethyl-
Formula C8H24O4Si4, MW 296, CAS# 556-67-2, Entry# 32128
Oktamethylcyklotetrasiloxan

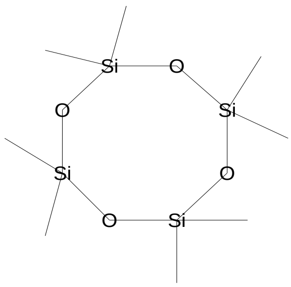

trisiloxane, 1,1,1,5,5,5-hexamethyl-3-[(trimethylsilyl)oxy]-
Formula C9H28O3Si4, MW 296, CAS# NA, Entry# 219896
1,1,1,5,5,5-hexamethyl-3-[(trimethylsilyl)oxy]trisiloxane

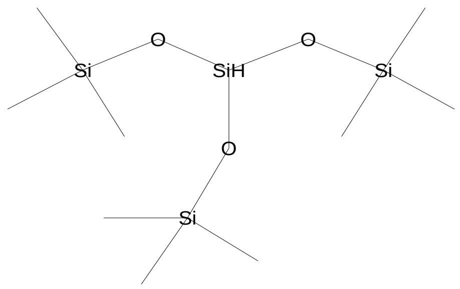

g-3 #2209 RT: 8.01 AV: 1 AV: 5 SB: 12 2202-2207 2211-2216 NL: 2.58E4
T: + c EI Full ms [33.00-450.00]

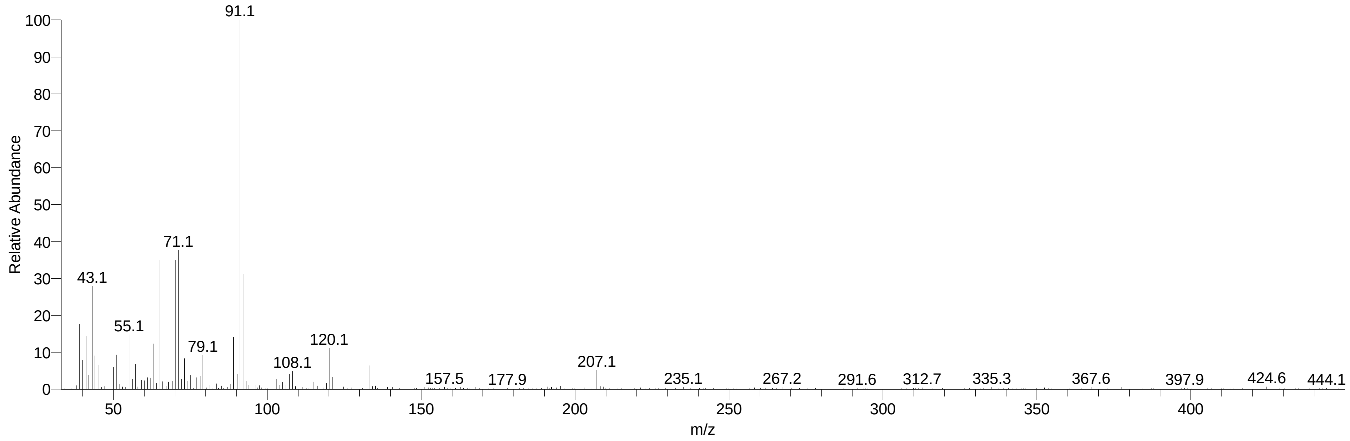

SI	Compound Name	RT	Cas #	Probability
633	Benzyl 2,3-anhydro-á-d-ribopyranoside	8.01	NA	8.36
625	Benzaldehyde, 3-benzyloxy-2-fluoro-4-methoxy-	8.01	NA	6.24
619	2-Butanone, 3-methyl-1-phenyl-	8.01	2893-05-2	4.90

Compound Structure

Benzyl 2,3-anhydro-á-d-ribopyranoside
Formula C12H14O4, MW 222, CAS# NA, Entry# 60494
Benzyl 2,3-anhydropentopyranoside #

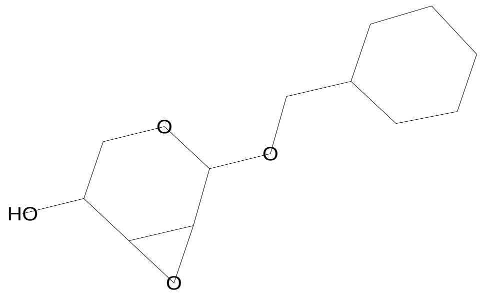

Benzaldehyde, 3-benzyloxy-2-fluoro-4-methoxy-
Formula C15H13FO3, MW 260, CAS# NA, Entry# 59644
3-(Benzyloxy)-2-fluoro-4-methoxybenzaldehyde #

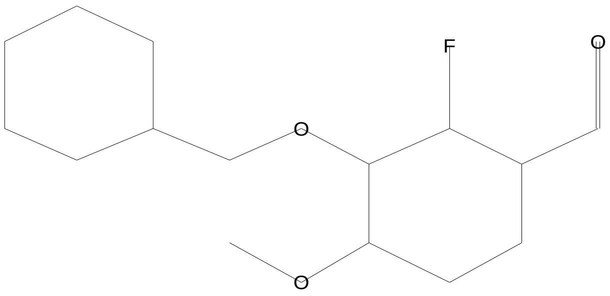

2-Butanone, 3-methyl-1-phenyl-
Formula C11H14O, MW 162, CAS# 2893-05-2, Entry# 2719
Benzyl isopropyl ketone

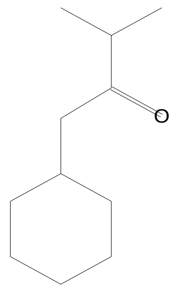

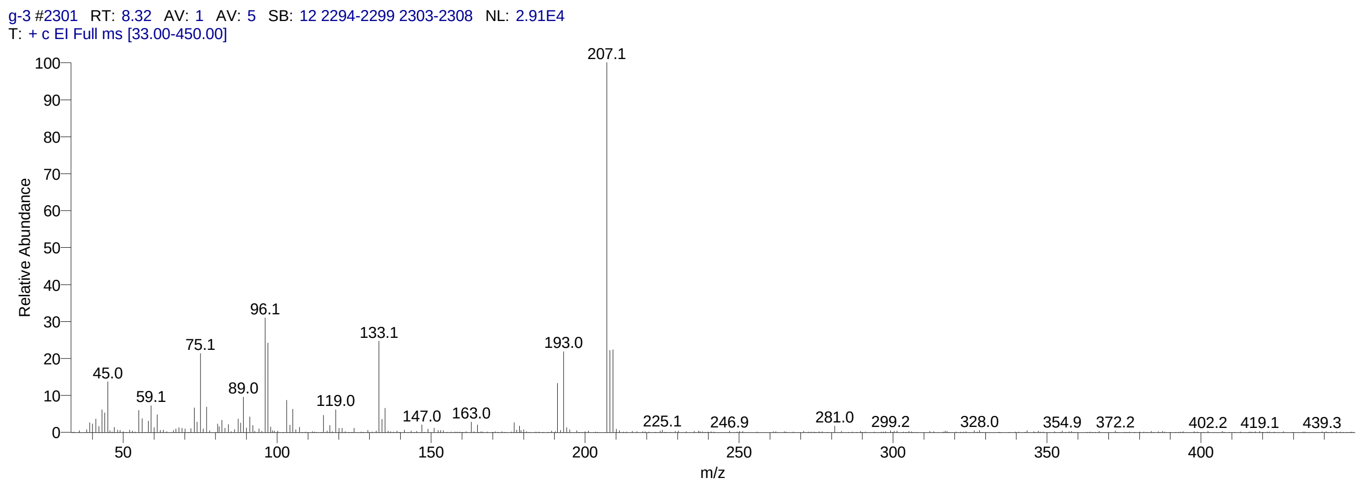

SI	Compound Name	RT	Cas #	Probability
707	Cyclotrisiloxane, hexamethyl-	8.32	541-05-9	63.49
705	Cyclotrisiloxane, hexamethyl-	8.32	541-05-9	63.49
682	Arsenous acid, tris(trimethylsilyl) ester	8.32	55429-29-3	19.36

Compound Structure

Cyclotrisiloxane, hexamethyl-
Formula C6H18O3Si3, MW 222, CAS# 541-05-9, Entry# 29125
Dimethylsiloxane cyclic trimer

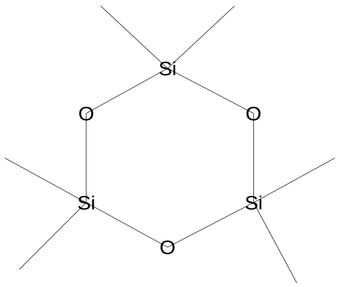

Cyclotrisiloxane, hexamethyl-
Formula C6H18O3Si3, MW 222, CAS# 541-05-9, Entry# 188945
Dimethylsiloxane cyclic trimer

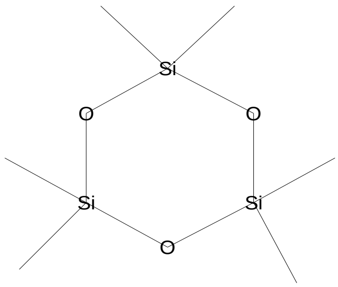

Arsenous acid, tris(trimethylsilyl) ester
Formula C9H27AsO3Si3, MW 342, CAS# 55429-29-3, Entry# 188948
$:28CWZNQWMMDOIFQC-UHFFFAOYSA-N

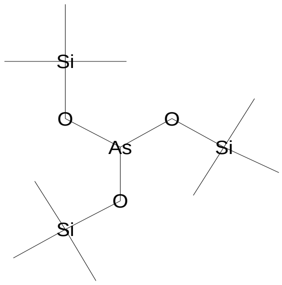

g-3 #2387 RT: 8.62 AV: 1 AV: 5 SB: 12 2380-2385 2389-2394 NL: 4.32E6
T: + c EI Full ms [33.00-450.00]

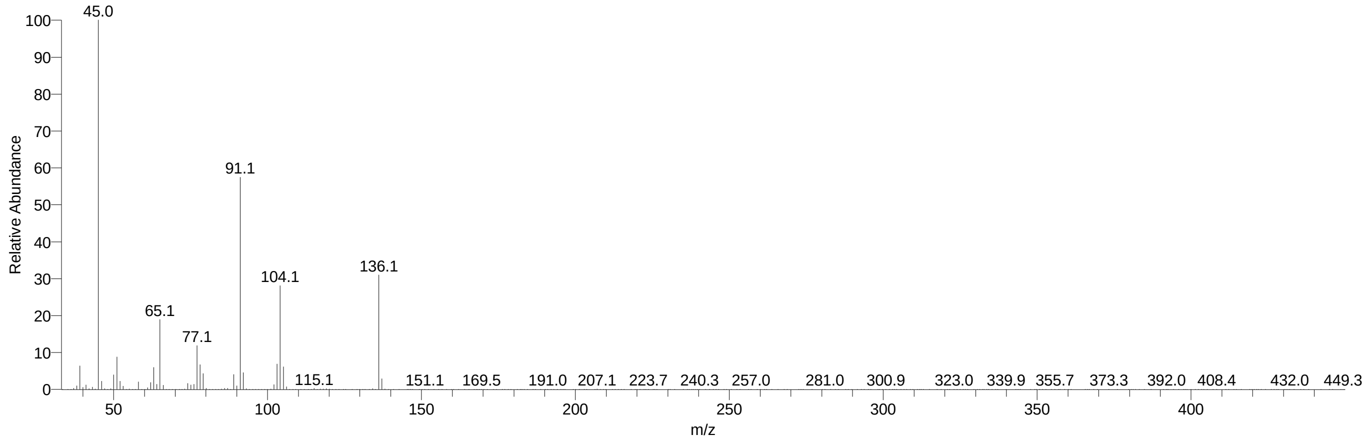

SI	Compound Name	RT	Cas #	Probability
929	Benzene, (2-methoxyethyl)-	8.62	3558-60-9	95.79
912	Benzene, (2-methoxyethyl)-	8.62	3558-60-9	95.79
867	Benzene, (2-methoxyethyl)-	8.62	3558-60-9	95.79

Compound Structure

Benzene, (2-methoxyethyl)-
Formula C9H12O, MW 136, CAS# 3558-60-9, Entry# 4204
Ether, methyl phenethyl

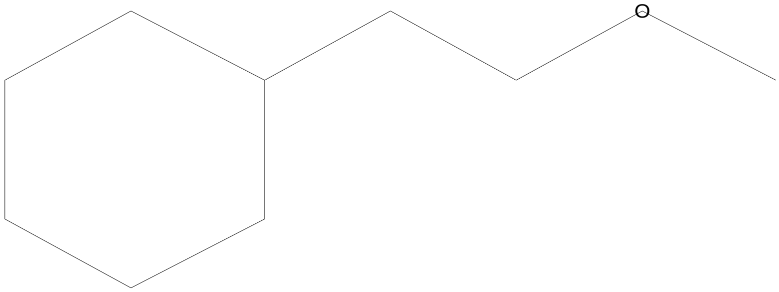

Benzene, (2-methoxyethyl)-
Formula C9H12O, MW 136, CAS# 3558-60-9, Entry# 17443
Ether, methyl phenethyl

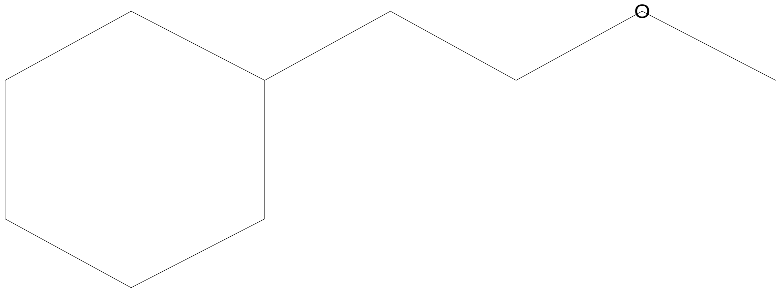

Benzene, (2-methoxyethyl)-
Formula C9H12O, MW 136, CAS# 3558-60-9, Entry# 13570
Ether, methyl phenethyl

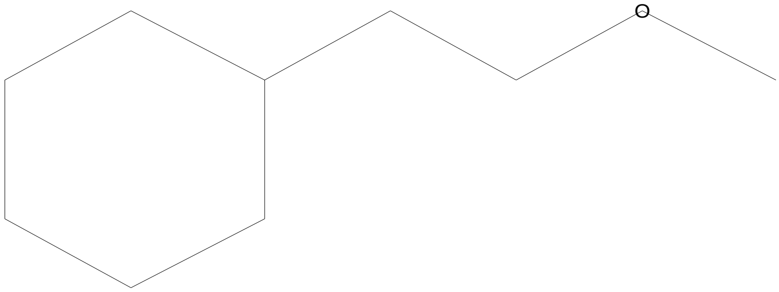

g-3 #2485 RT: 8.95 AV: 1 AV: 5 SB: 12 2478-2483 2487-2492 NL: 1.41E5
T: + c EI Full ms [33.00-450.00]

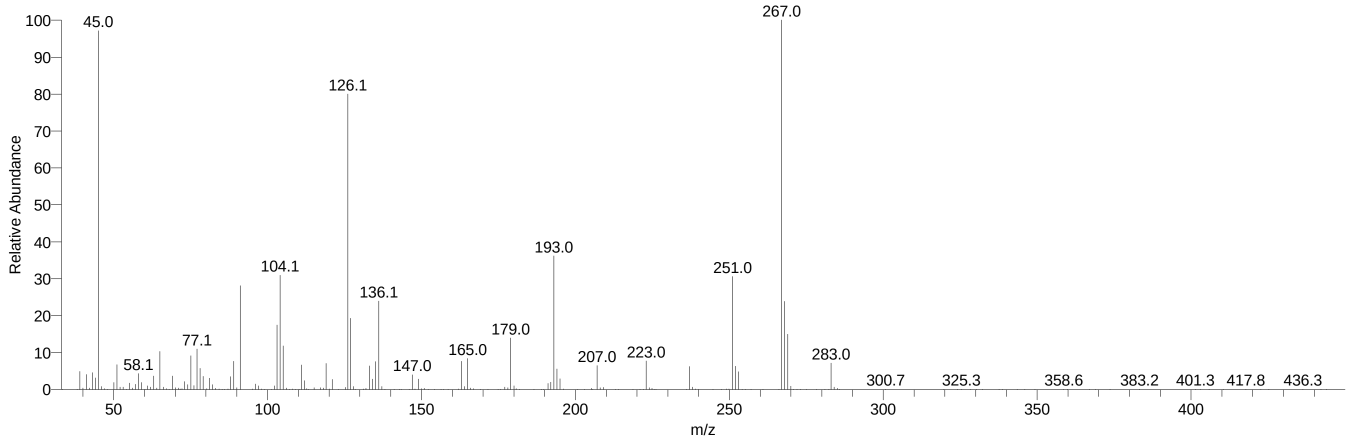

SI	Compound Name	RT	Cas #	Probability
581	4-Hydroxybenzoic acid, 2TMS derivative	8.95	2078-13-9	14.70
578	3-Hydroxybenzoic acid, 2TMS derivative	8.95	3782-84-1	12.99
577	3-Hydroxybenzoic acid, 2TMS derivative	8.95	3782-84-1	12.99

Compound Structure

4-Hydroxybenzoic acid, 2TMS derivative
Formula C13H22O3Si2, MW 282, CAS# 2078-13-9, Entry# 10205
Benzoic acid, 4-[(trimethylsilyl)oxy]-, trimethylsilyl ester

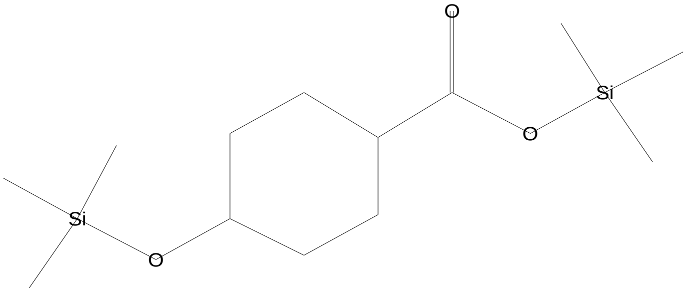

3-Hydroxybenzoic acid, 2TMS derivative
Formula C13H22O3Si2, MW 282, CAS# 3782-84-1, Entry# 215820
Benzoic acid, 3-[(trimethylsilyl)oxy]-, trimethylsilyl ester

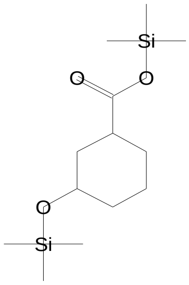

3-Hydroxybenzoic acid, 2TMS derivative
Formula C13H22O3Si2, MW 282, CAS# 3782-84-1, Entry# 31713
Benzoic acid, 3-[(trimethylsilyl)oxy]-, trimethylsilyl ester

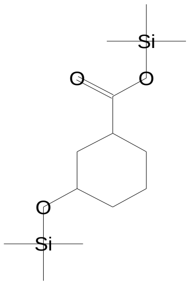

g-3 #2612 RT: 9.38 AV: 1 AV: 5 SB: 12 2605-2610 2614-2619 NL: 2.37E6
T: + c EI Full ms [33.00-450.00]

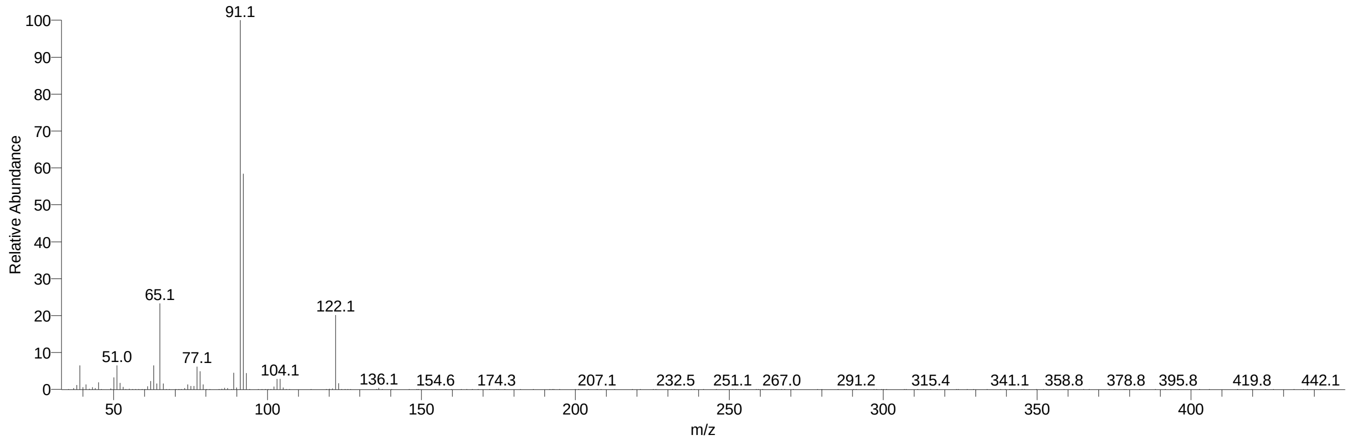

SI	Compound Name	RT	Cas #	Probability
919	Phenylethyl Alcohol	9.38	60-12-8	80.57
919	Phenylethyl Alcohol	9.38	60-12-8	80.57
915	Phenylethyl Alcohol	9.38	60-12-8	80.57

Compound Structure

Phenylethyl Alcohol
Formula C8H10O, MW 122, CAS# 60-12-8, Entry# 2079
Benzeneethanol

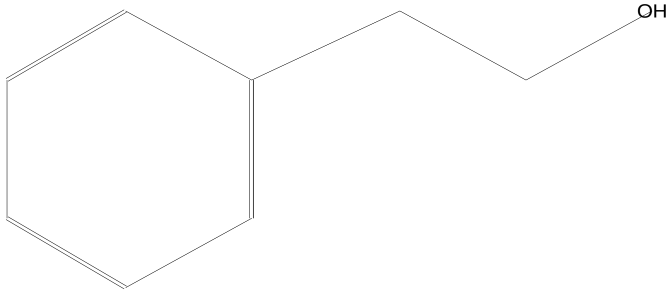

Phenylethyl Alcohol
Formula C8H10O, MW 122, CAS# 60-12-8, Entry# 60552
Benzeneethanol

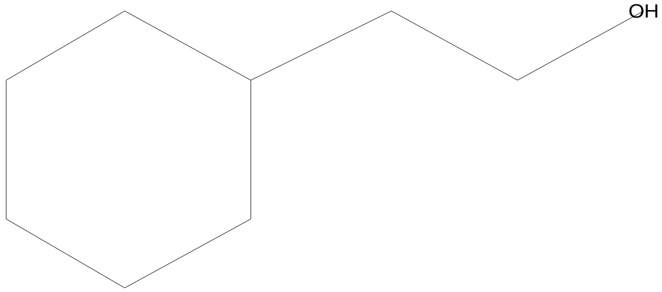

Phenylethyl Alcohol
Formula C8H10O, MW 122, CAS# 60-12-8, Entry# 13237
Benzeneethanol

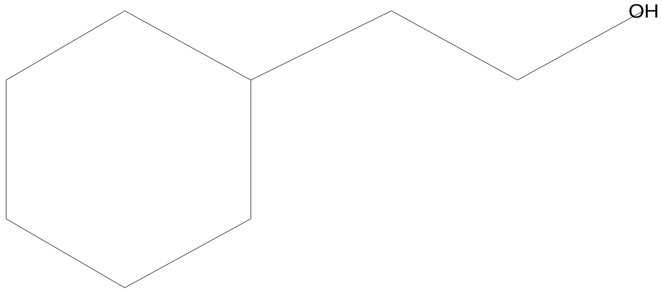

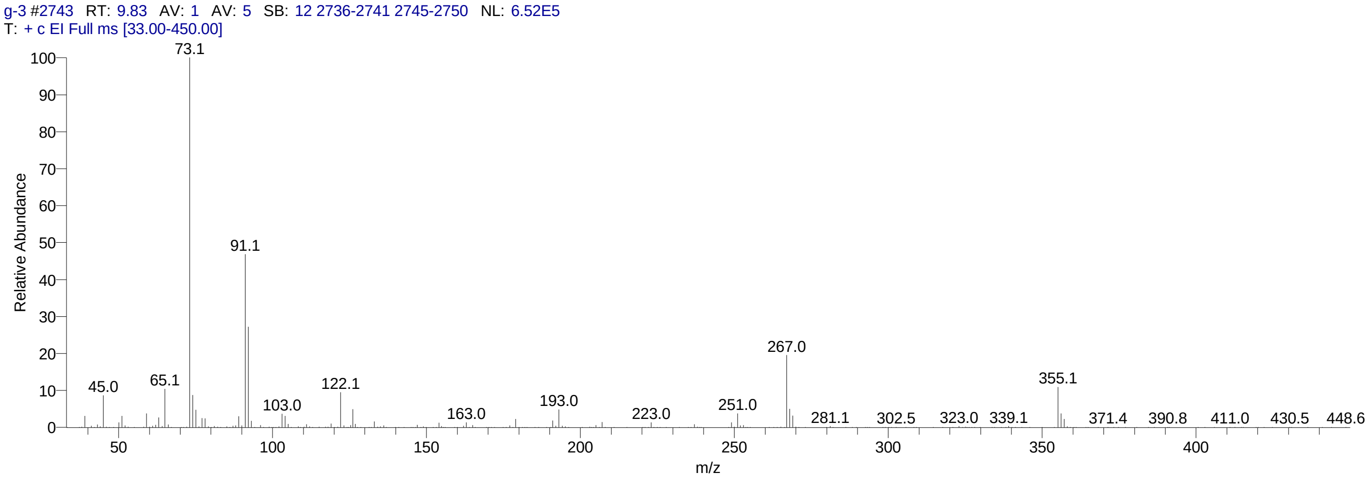

SI	Compound Name	RT	Cas #	Probability
644	Cyclopentasiloxane, decamethyl-	9.83	541-02-6	59.07
633	Cyclopentasiloxane, decamethyl-	9.83	541-02-6	59.07
593	Cyclopentasiloxane, decamethyl-	9.83	541-02-6	59.07

Compound Structure

Cyclopentasiloxane, decamethyl-
Formula C10H30O5Si5, MW 370, CAS# 541-02-6, Entry# 10279
Decamethylcyclopentasiloxane

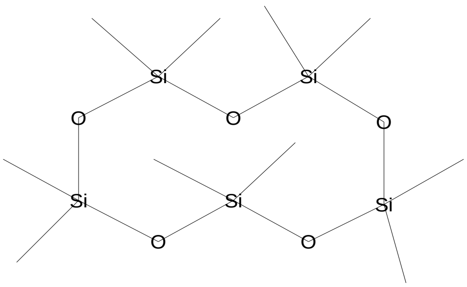

Cyclopentasiloxane, decamethyl-
Formula C10H30O5Si5, MW 370, CAS# 541-02-6, Entry# 33275
Decamethylcyclopentasiloxane

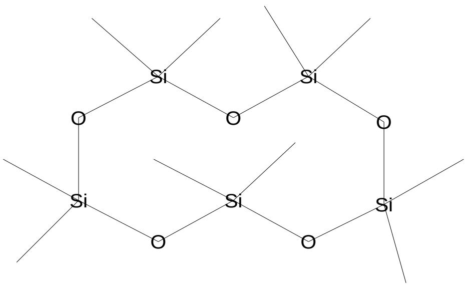

Cyclopentasiloxane, decamethyl-
Formula C10H30O5Si5, MW 370, CAS# 541-02-6, Entry# 234336
Decamethylcyclopentasiloxane

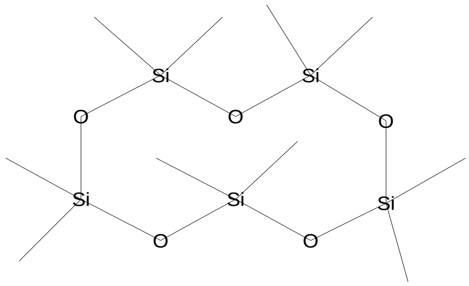

g-3 #3398 RT: 12.05 AV: 1 AV: 5 SB: 12 3391-3396 3400-3405 NL: 1.79E5
T: + c EI Full ms [33.00-450.00]

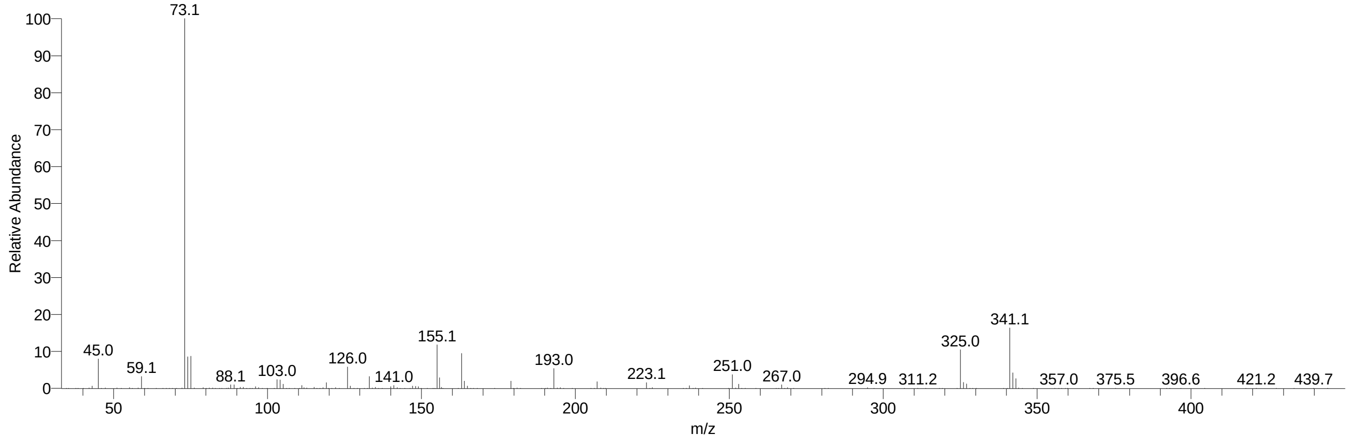

SI	Compound Name	RT	Cas #	Probability
550	Phosphonoacetic Acid, 3TMS derivative	12.05	53044-27-2	63.72
515	1,1,1,3,5,7,9,11,11,11-Decamethyl-5-(trimethylsiloxy)hexas iloxane	12.05	50694-26-3	15.99
488	Cyclohexasiloxane, dodecamethyl-	12.05	540-97-6	4.70

Compound Structure

Phosphonoacetic Acid, 3TMS derivative
Formula C11H29O5PSi3, MW 356, CAS# 53044-27-2, Entry# 232513
Acetic acid, [bis[(trimethylsilyl)oxy]phosphinyl]-, trimethylsilyl ester

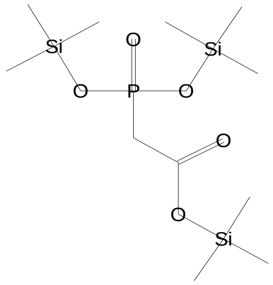

1,1,1,3,5,7,9,11,11,11-Decamethyl-5-(trimethylsiloxy)hexasiloxane
Formula C13H42O6Si7, MW 490, CAS# 50694-26-3, Entry# 42769
1,1,1,3,5,7,9,11,11,11-Decamethyl-5-[(trimethylsilyl)oxy]hexasiloxane #

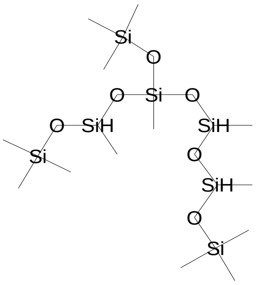

Cyclohexasiloxane, dodecamethyl-
Formula C12H36O6Si6, MW 444, CAS# 540-97-6, Entry# 10269
Dodecamethylcyclohexasiloxane

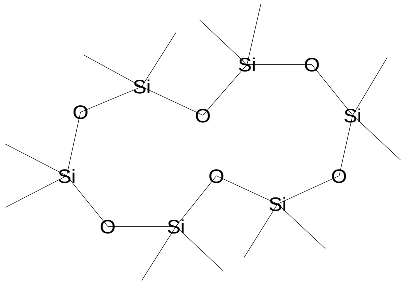

g-3 #3481 RT: 12.34 AV: 1 AV: 5 SB: 12 3474-3479 3483-3488 NL: 9.98E4
T: + c EI Full ms [33.00-450.00]

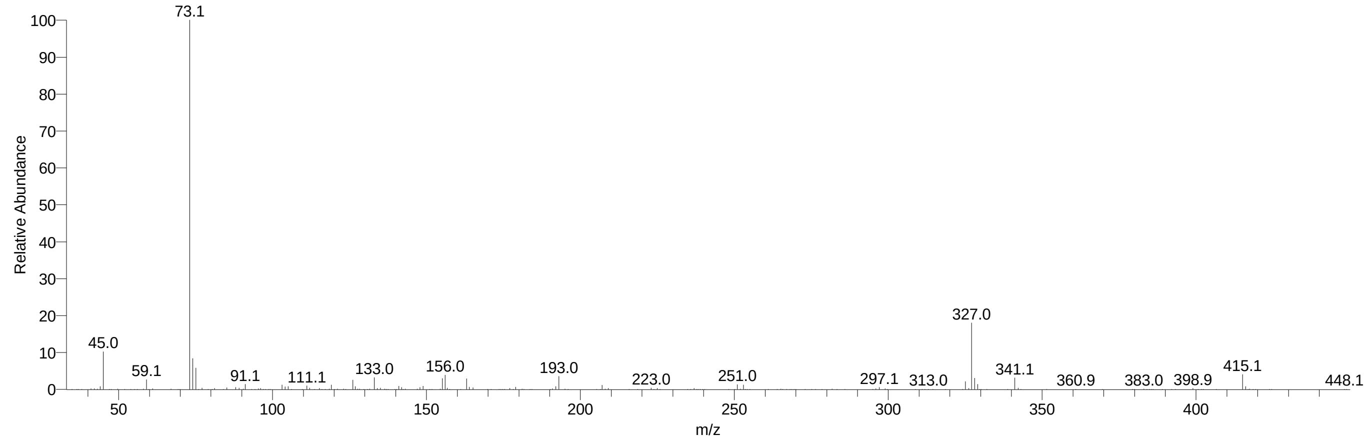

SI	Compound Name	RT	Cas #	Probability
578	Tetrasiloxane, 3,5-diethoxy-1,1,1,7,7,7-hexamethyl-3,5-bis(trimethylsiloxy)-	12.34	72439-78-2	29.87
573	3,5-Diisopropoxy-1,1,1,7,7,7-hexamethyl-3,5-bis(trimethylsiloxy)tetrasiloxane	12.34	71579-67-4	24.08
567	3,5-Dibutoxy-1,1,1,7,7,7-hexamethyl-3,5-bis(trimethylsiloxy)tetrasiloxane	12.34	72439-85-1	18.92

Compound Structure

Tetrasiloxane, 3,5-diethoxy-1,1,1,7,7,7-hexamethyl-3,5-bis(trimethylsiloxy)-
Formula C16H46O7Si6, MW 518, CAS# 72439-78-2, Entry# 44057
1-Ethoxy-3,3,3-trimethyl-1-[(trimethylsilyl)oxy]disiloxanyl ethyl bis(trimethylsilyl) orthosilicate #

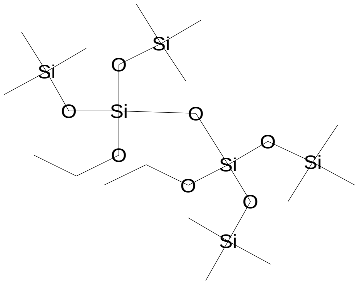

3,5-Diisopropoxy-1,1,1,7,7,7-hexamethyl-3,5-bis(trimethylsiloxy)tetrasiloxane
Formula C18H50O7Si6, MW 546, CAS# 71579-67-4, Entry# 40678
1-Isopropoxy-3,3,3-trimethyl-1-[(trimethylsilyl)oxy]disiloxanyl isopropyl bis(trimethylsilyl) orthosilicate #

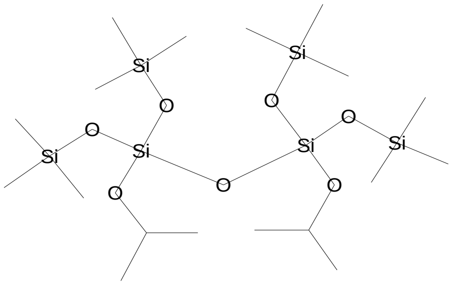

3,5-Dibutoxy-1,1,1,7,7,7-hexamethyl-3,5-bis(trimethylsiloxy)tetrasiloxane
Formula C20H54O7Si6, MW 574, CAS# 72439-85-1, Entry# 40543
1-Butoxy-3,3,3-trimethyl-1-[(trimethylsilyl)oxy]disiloxanyl butyl bis(trimethylsilyl) orthosilicate #

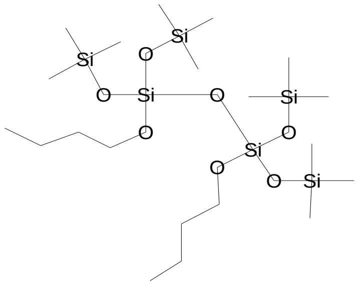

g-3 #3753 RT: 13.26 AV: 1 AV: 5 SB: 12 3746-3751 3755-3760 NL: 2.55E5
T: + c EI Full ms [33.00-450.00]

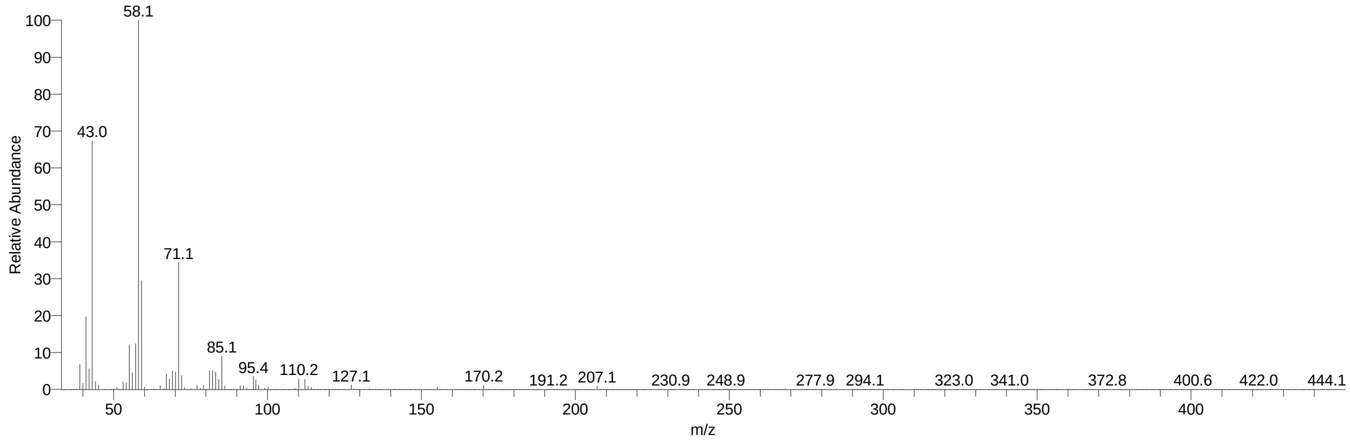

SI	Compound Name	RT	Cas #	Probability
903	2-Undecanone	13.26	112-12-9	74.07
871	2-Undecanone	13.26	112-12-9	74.07
868	2-Undecanone	13.26	112-12-9	74.07

Compound Structure

2-Undecanone
Formula C11H22O, MW 170, CAS# 112-12-9, Entry# 2415
Ketone, methyl nonyl

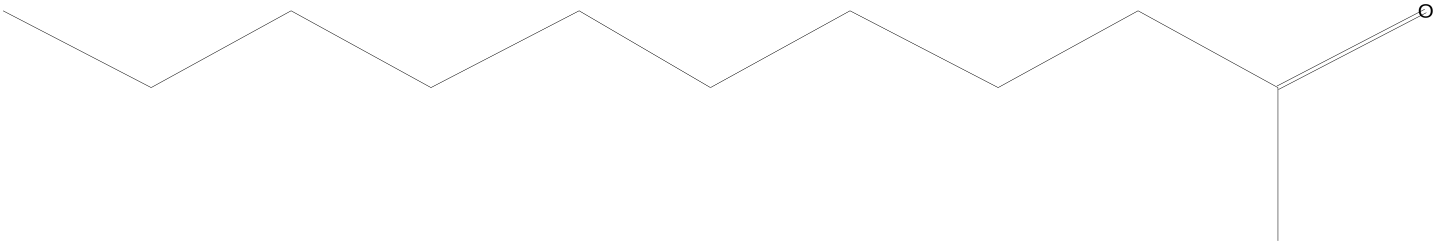

2-Undecanone
Formula C11H22O, MW 170, CAS# 112-12-9, Entry# 2414
Ketone, methyl nonyl

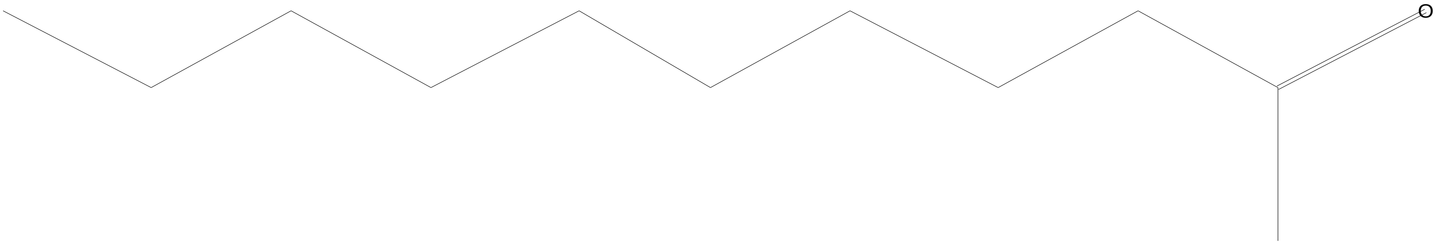

2-Undecanone
Formula C11H22O, MW 170, CAS# 112-12-9, Entry# 17
Ketone, methyl nonyl

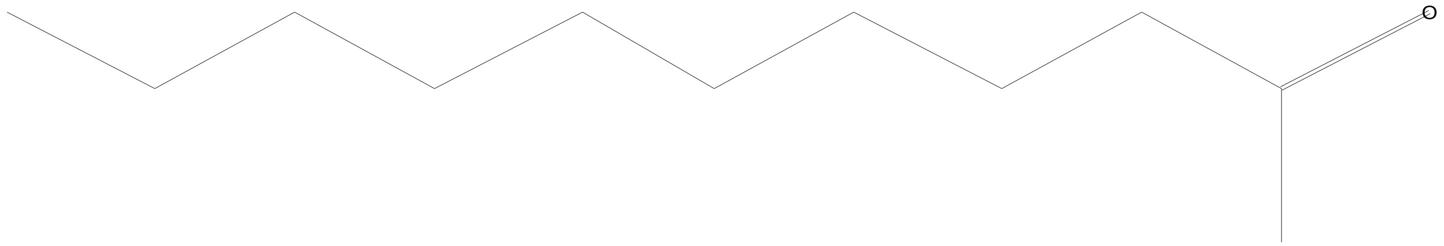

g-3 #3805 RT: 13.44 AV: 1 AV: 5 SB: 12 3798-3803 3807-3812 NL: 1.04E5
T: + c EI Full ms [33.00-450.00]

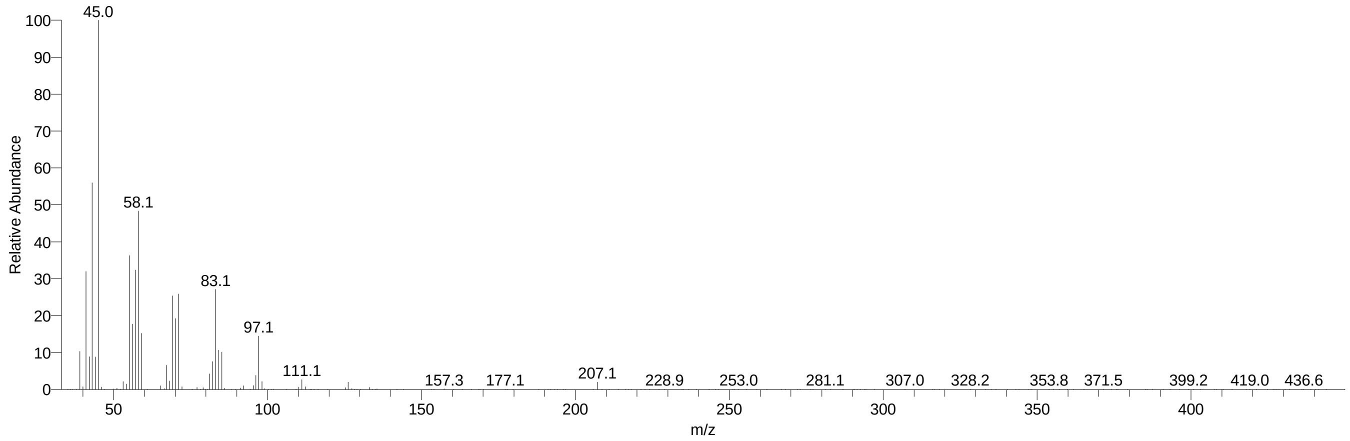

SI	Compound Name	RT	Cas #	Probability
809	2-Tetradecanol	13.44	4706-81-4	13.25
808	2-Undecanol	13.44	1653-30-1	12.73
807	2-Pentadecanol	13.44	1653-34-5	12.24

Compound Structure

2-Tetradecanol
Formula C14H30O, MW 214, CAS# 4706-81-4, Entry# 3997
sec-Tetradecyl alcohol

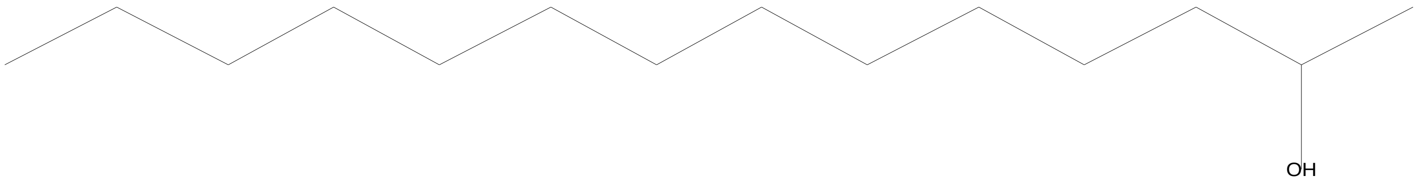

2-Undecanol
Formula C11H24O, MW 172, CAS# 1653-30-1, Entry# 3963
sec-Undecyl Alcohol

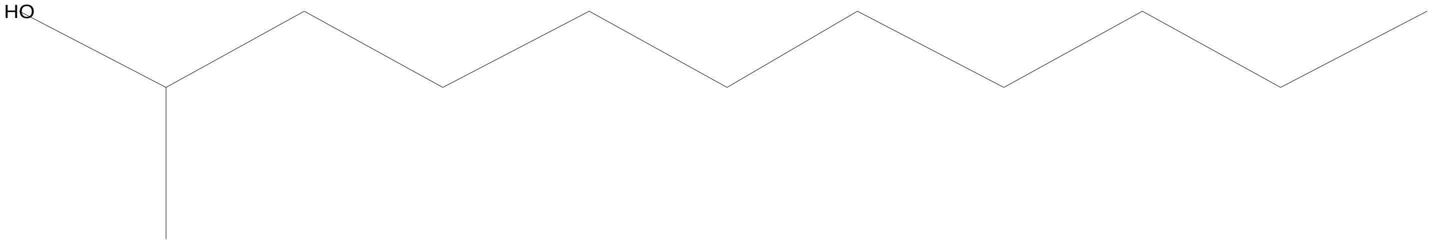

2-Pentadecanol
Formula $C_{15}H_{32}O$, MW 228, CAS# 1653-34-5, Entry# 16819
sec-Pentadecyl alcohol

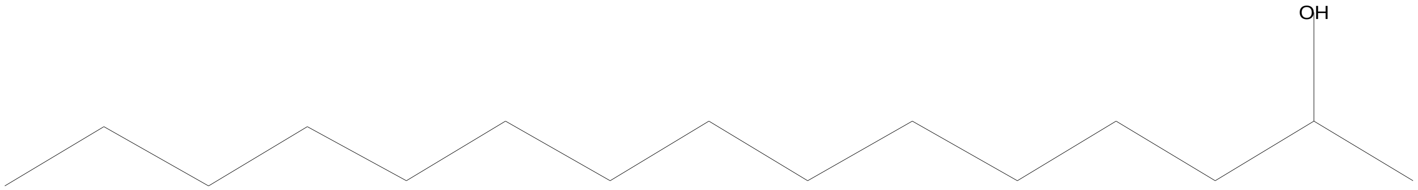

g-3 #3923 RT: 13.84 AV: 1 AV: 5 SB: 12 3916-3921 3925-3930 NL: 5.65E5
T: + c EI Full ms [33.00-450.00]

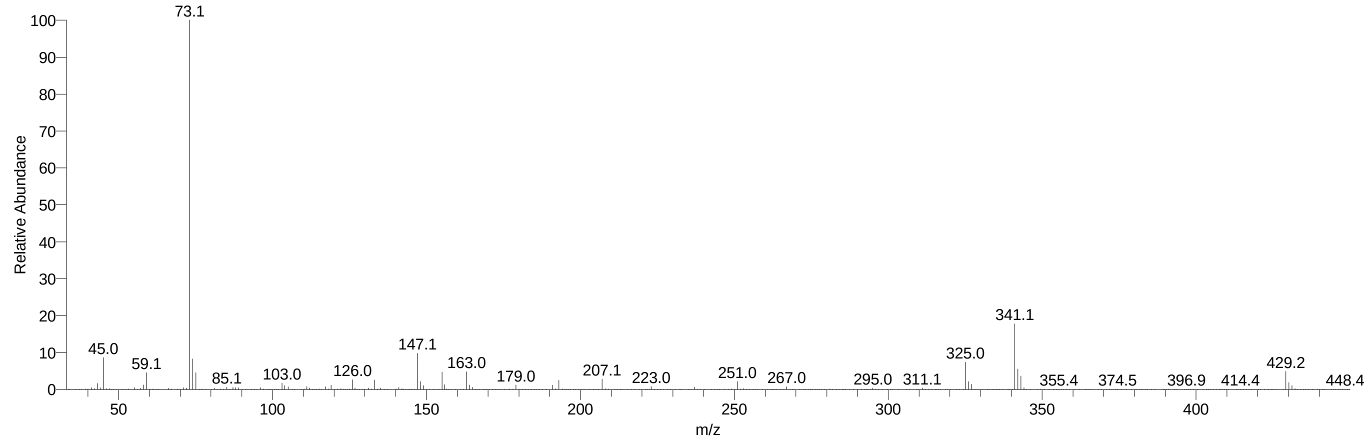

SI	Compound Name	RT	Cas #	Probability
737	Cyclohexasiloxane, dodecamethyl-	13.84	540-97-6	92.06
736	Cyclohexasiloxane, dodecamethyl-	13.84	540-97-6	92.06
728	Cyclohexasiloxane, dodecamethyl-	13.84	540-97-6	92.06

Compound Structure

Cyclohexasiloxane, dodecamethyl-
Formula C12H36O6Si6, MW 444, CAS# 540-97-6, Entry# 10270
Dodecamethylcyclohexasiloxane

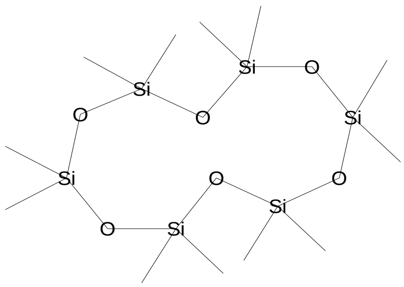

Cyclohexasiloxane, dodecamethyl-
Formula C12H36O6Si6, MW 444, CAS# 540-97-6, Entry# 10269
Dodecamethylcyclohexasiloxane

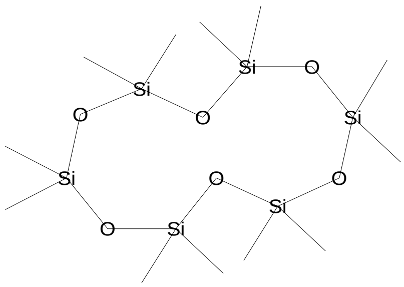

Cyclohexasiloxane, dodecamethyl-
Formula C12H36O6Si6, MW 444, CAS# 540-97-6, Entry# 44297
Dodecamethylcyclohexasiloxane

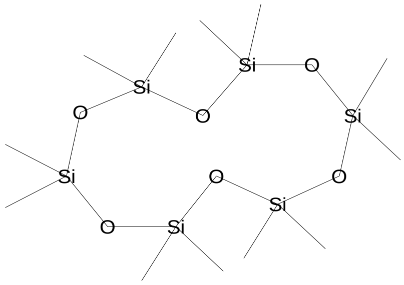

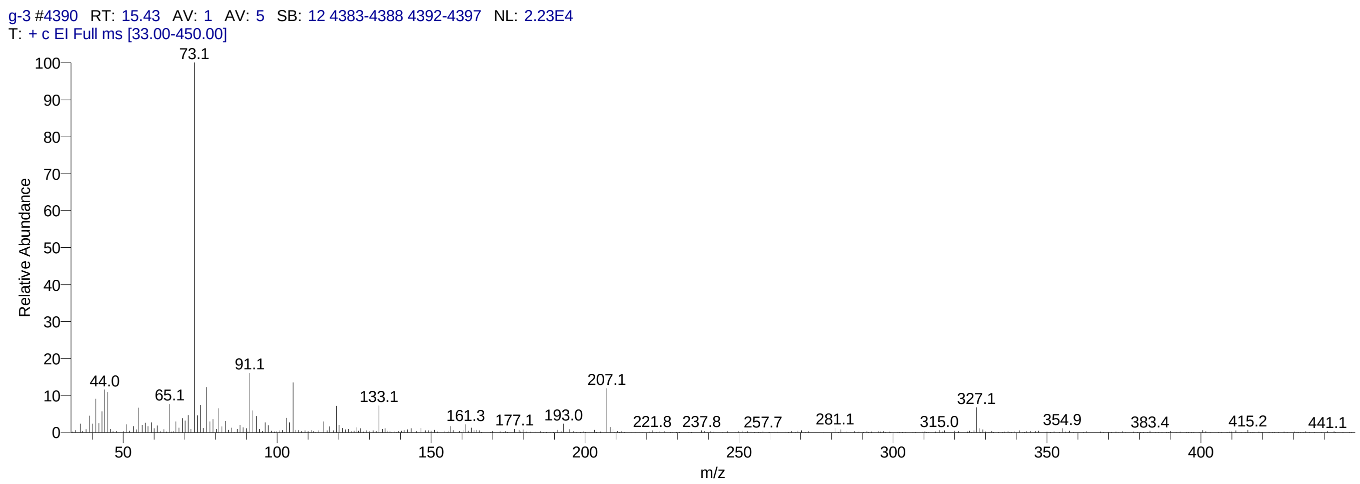

SI	Compound Name	RT	Cas #	Probability
574	.psi.,.psi.-Carotene, 3,4-didehydro-1,1',2,2'-tetrahydro-1'-hydroxy-1-methoxy-	15.43	5017-53-8	15.54
571	.psi.,.psi.-Carotene, 3,4-didehydro-1,2-dihydro-1-methoxy-	15.43	5085-16-5	13.73
546	Silane, (2-ethyl-5,5-dimethyl-4-methylene-1-cyclopenten-1-yl)trimethyl-	15.43	95798-05-3	4.18

Compound Structure

.psi.,.psi.-Carotene, 3,4-didehydro-1,1',2,2'-tetrahydro-1'-hydroxy-1-methoxy-
Formula C41H60O2, MW 584, CAS# 5017-53-8, Entry# 41742
Lycopene, 3,4-didehydro-1,1',2,2'-tetrahydro-1'-hydroxy-1-methoxy-, all-trans-

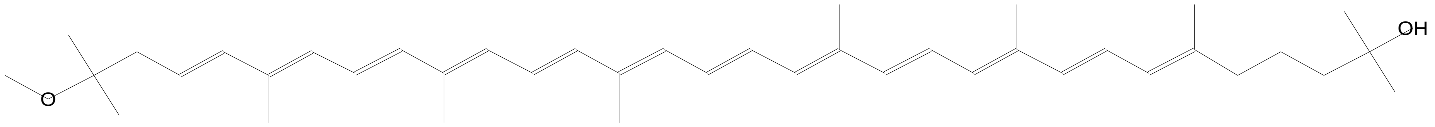

.psi.,.psi.-Carotene, 3,4-didehydro-1,2-dihydro-1-methoxy-
Formula C41H58O, MW 566, CAS# 5085-16-5, Entry# 60103
Lycopene, 3,4-didehydro-1,2-dihydro-1-methoxy-, all-trans-

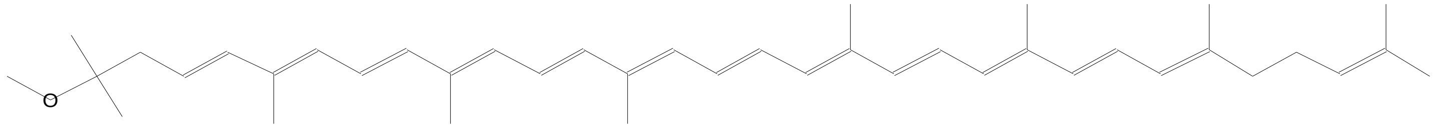

Silane, (2-ethyl-5,5-dimethyl-4-methylene-1-cyclopenten-1-yl)trimethyl-
Formula C13H24Si, MW 208, CAS# 95798-05-3, Entry# 41081
(2-Ethyl-5,5-dimethyl-4-methylene-1-cyclopenten-1-yl)(trimethyl)silane #

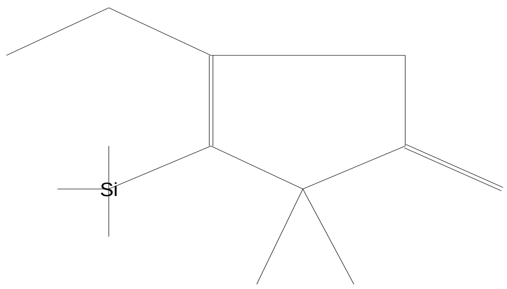

g-3 #4488 RT: 15.76 AV: 1 AV: 5 SB: 12 4481-4486 4490-4495 NL: 8.45E4
T: + c EI Full ms [33.00-450.00]

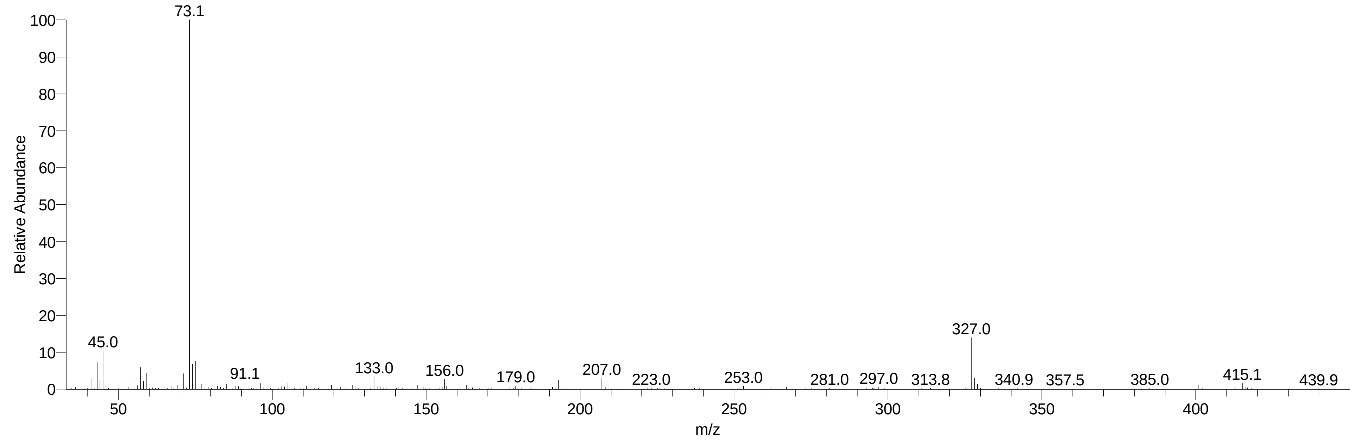

SI	Compound Name	RT	Cas #	Probability
627	Octadecane-1,2-diol, 2TMS derivative	15.76	NA	39.72
614	16-Methyl-heptadecane-1,2-diol, trimethylsilyl ether	15.76	NA	25.66
573	3,5-Dibutoxy-1,1,1,7,7,7-hexamethyl-3,5-bis(trimethylsiloxy)tetrasiloxane	15.76	72439-85-1	5.85

Compound Structure

Octadecane-1,2-diol, 2TMS derivative
Formula C24H54O2Si2, MW 430, CAS# NA, Entry# 230257
Octadecane-1,2-diol, bis(trimethylsilyl) ether

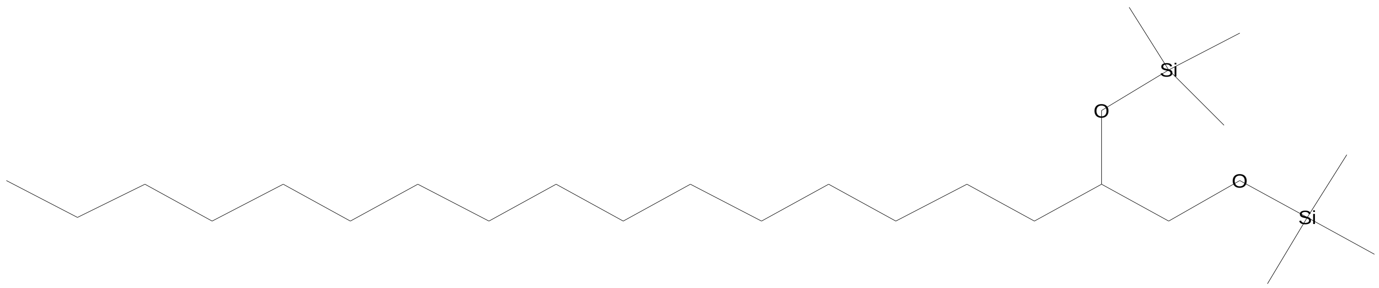

16-Methyl-heptadecane-1,2-diol, trimethylsilyl ether
Formula C24H54O2Si2, MW 430, CAS# NA, Entry# 230258
$:28VVPUYYNCVZRCTJ-UHFFFAOYSA-N

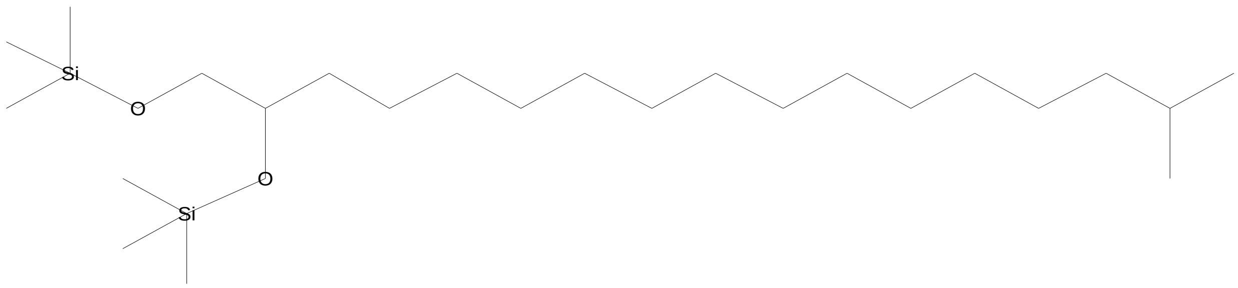

3,5-Dibutoxy-1,1,1,7,7,7-hexamethyl-3,5-bis(trimethylsiloxy)tetrasiloxane
Formula C20H54O7Si6, MW 574, CAS# 72439-85-1, Entry# 40543
1-Butoxy-3,3,3-trimethyl-1-[(trimethylsilyl)oxy]disiloxanyl butyl bis(trimethylsilyl) orthosilicate #

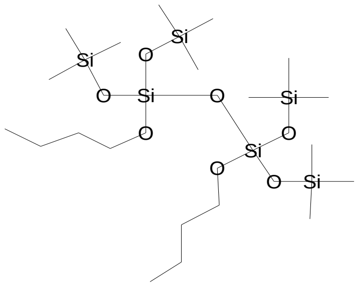

g-3 #5079 RT: 17.77 AV: 1 AV: 5 SB: 12 5072-5077 5081-5086 NL: 5.31E3
T: + c EI Full ms [33.00-450.00]

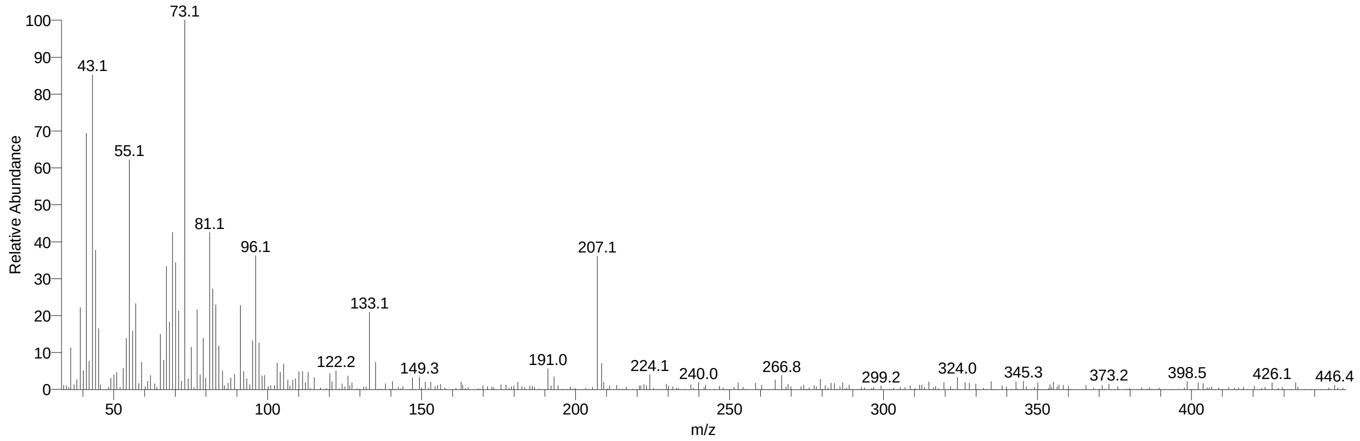

SI	Compound Name	RT	Cas #	Probability
577	Octadecanal, 2-bromo-	17.77	56599-95-2	21.49
558	Ethyl iso-allocholate	17.77	NA	10.43
536	12-Methyl-E,E-2,13-octadecadien-1-ol	17.77	NA	4.12

Compound Structure

Octadecanal, 2-bromo-
Formula C18H35BrO, MW 346, CAS# 56599-95-2, Entry# 7651
2-Bromooctadecanal #

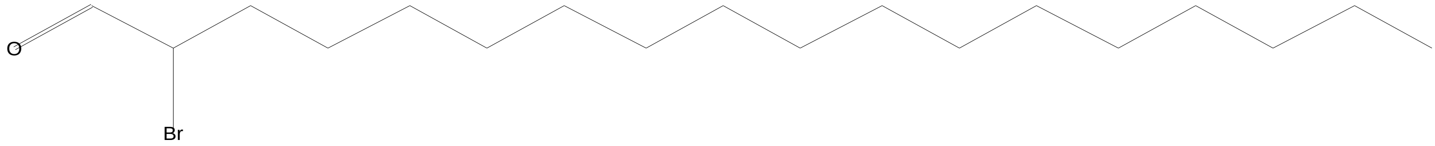

Ethyl iso-allocholate
Formula C26H44O5, MW 436, CAS# NA, Entry# 7020
Ethyl 3,7,12-trihydroxycholan-24-oate #

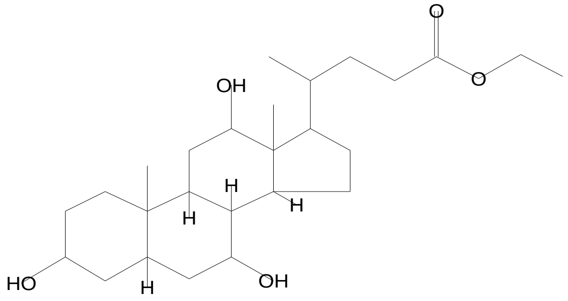

12-Methyl-E,E-2,13-octadecadien-1-ol
Formula C19H36O, MW 280, CAS# NA, Entry# 19016
(2E,15Z)-14-Methyl-2,15-octadecadien-1-ol #

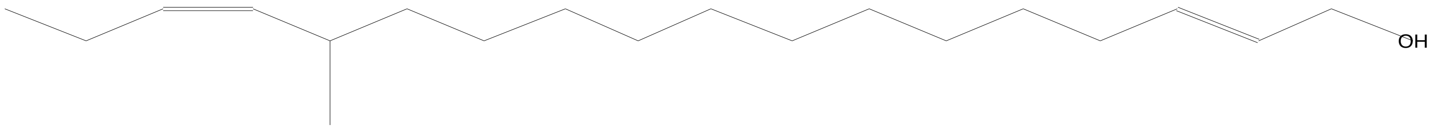

g-3 #5134 RT: 17.96 AV: 1 AV: 5 SB: 12 5127-5132 5136-5141 NL: 9.04E4
T: + c EI Full ms [33.00-450.00]

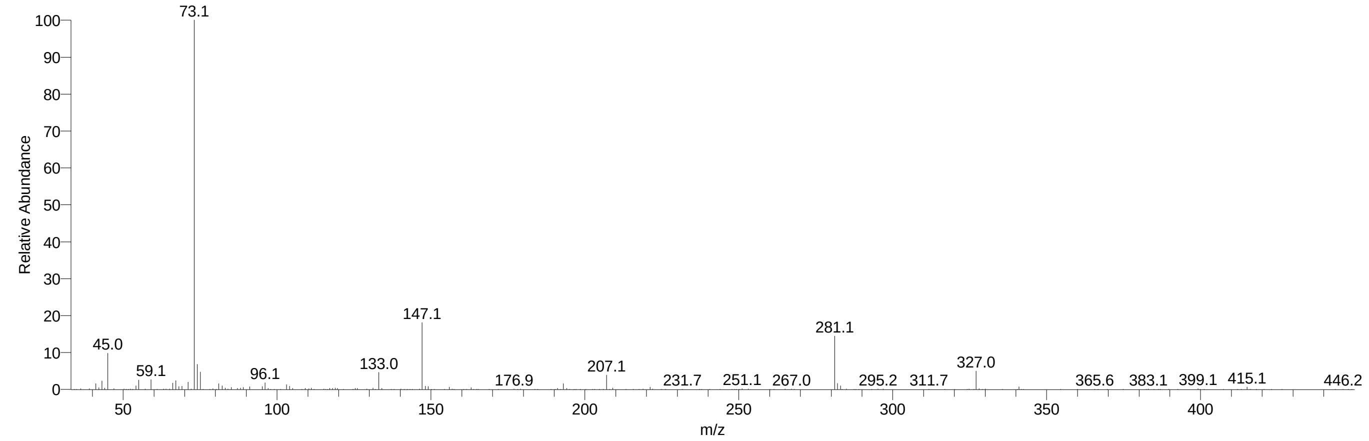

SI	Compound Name	RT	Cas #	Probability
585	3-Methylsalicylic acid, 2TMS derivative	17.96	NA	12.86
584	3-Isopropoxy-1,1,1,7,7,7-hexamethyl-3,5,5-tris(trimethylsil oxy)tetrasiloxane	17.96	71579-69-6	12.36
573	Trisiloxane, 1,1,1,5,5,5-hexamethyl-3,3-bis[(trimethylsilyl)oxy]-	17.96	3555-47-3	8.48

Compound Structure

3-Methylsalicylic acid, 2TMS derivative
Formula C14H24O3Si2, MW 296, CAS# NA, Entry# 219916
Benzoic acid, 3-methyl-2-trimethylsilyloxy-, trimethylsilyl ester

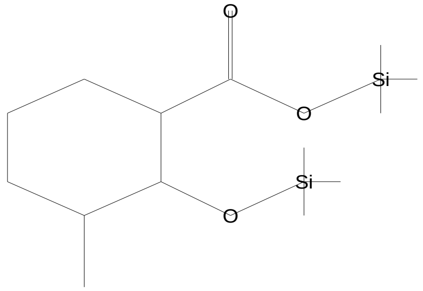

3-Isopropoxy-1,1,1,7,7,7-hexamethyl-3,5,5-tris(trimethylsiloxy)tetrasiloxane
Formula C18H52O7Si7, MW 576, CAS# 71579-69-6, Entry# 42479
1-Isopropoxy-3,3,3-trimethyl-1-[(trimethylsilyl)oxy]disiloxanyl tris(trimethylsilyl) orthosilicate #

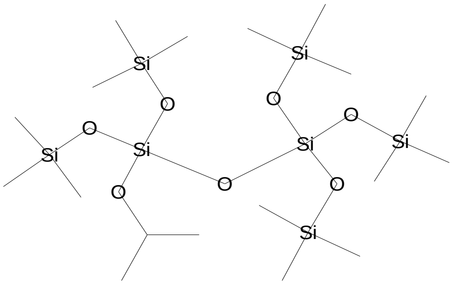

Trisiloxane, 1,1,1,5,5,5-hexamethyl-3,3-bis[(trimethylsilyl)oxy]-
Formula C12H36O4Si5, MW 384, CAS# 3555-47-3, Entry# 42730
Tetrakis(trimethylsiloxy)silane

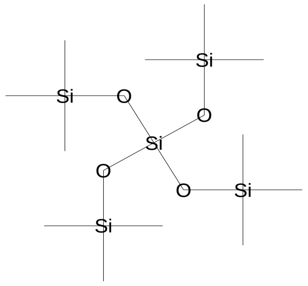

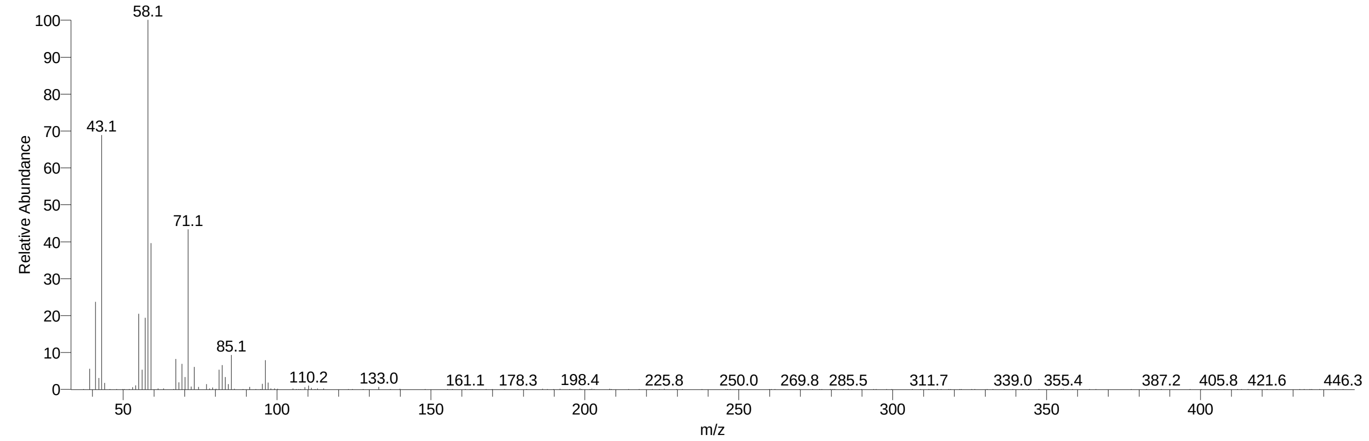

SI	Compound Name	RT	Cas #	Probability
795	2-Tetradecanone	18.13	2345-27-9	21.50
787	2-Tetradecanone	18.13	2345-27-9	21.50
782	2-Dodecanone	18.13	6175-49-1	13.89

Compound Structure

2-Tetradecanone
Formula C14H28O, MW 212, CAS# 2345-27-9, Entry# 6795
$:28POQLVOYRGNFGRM-UHFFFAOYSA-N

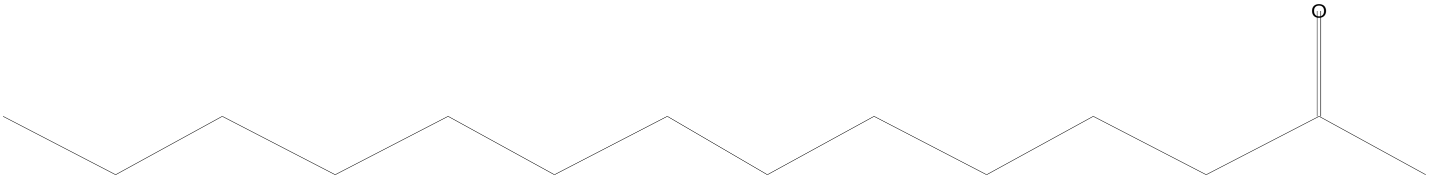

2-Tetradecanone
Formula C14H28O, MW 212, CAS# 2345-27-9, Entry# 6794
$:28POQLVOYRGNFGRM-UHFFFAOYSA-N

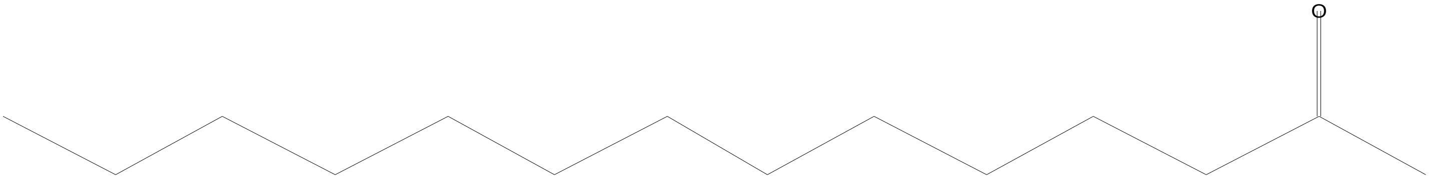

2-Dodecanone
Formula C12H24O, MW 184, CAS# 6175-49-1, Entry# 6802
Decyl methyl ketone

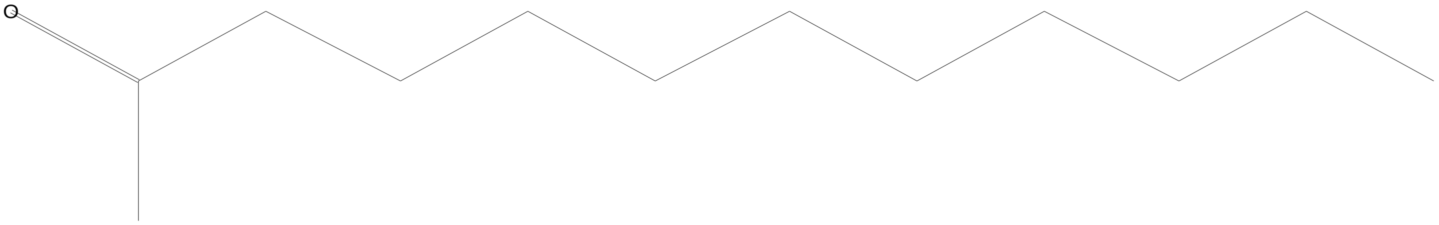

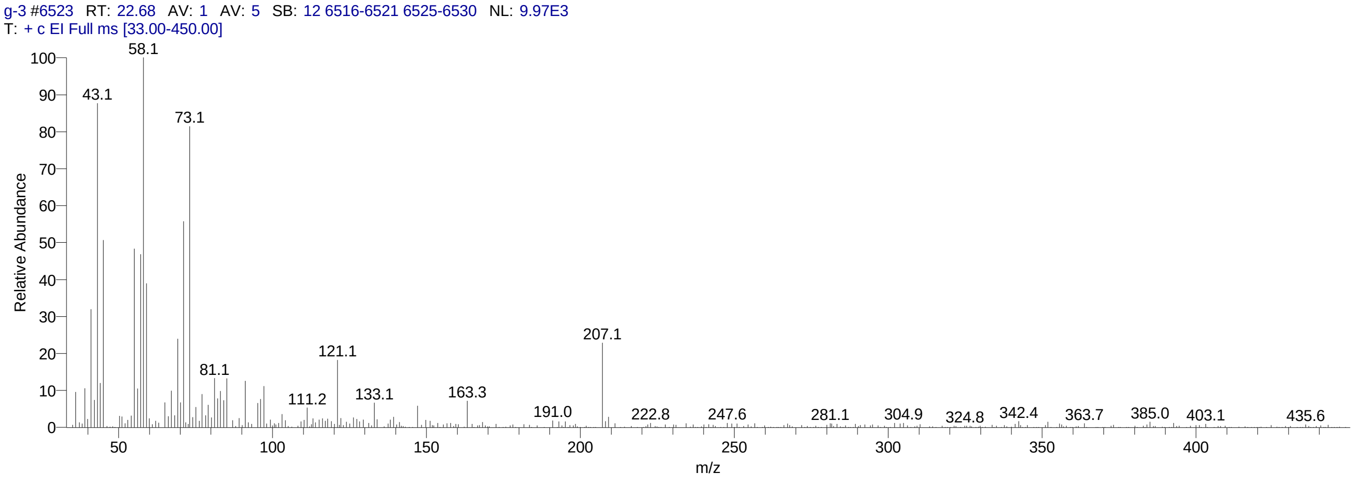

SI	Compound Name	RT	Cas #	Probability
601	[1,1'-Bicyclopropyl]-2-octanoic acid, 2'-hexyl-, methyl ester	22.68	56687-68-4	18.33
597	2-Hexadecanol	22.68	14852-31-4	15.49
576	9-Octadecenoic acid, (2-phenyl-1,3-dioxolan-4-yl)methyl ester, cis-	22.68	56599-45-2	6.59

Compound Structure

[1,1'-Bicyclopropyl]-2-octanoic acid, 2'-hexyl-, methyl ester
Formula C21H38O2, MW 322, CAS# 56687-68-4, Entry# 40749
$:28BNXIGQHDTCPKMN-UHFFFAOYSA-N

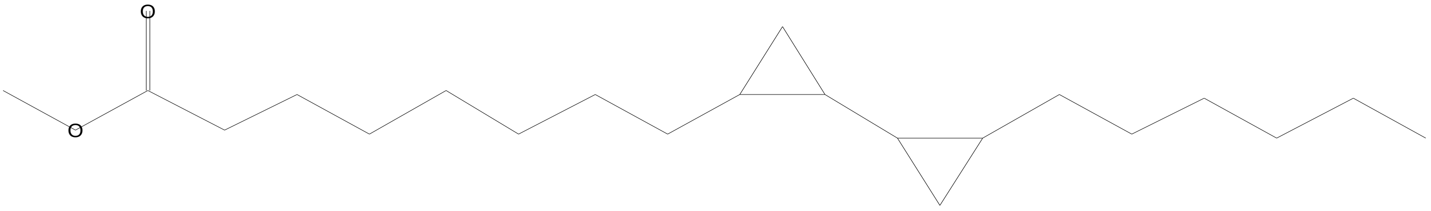

2-Hexadecanol
Formula C16H34O, MW 242, CAS# 14852-31-4, Entry# 4015
Hexadecanol-2

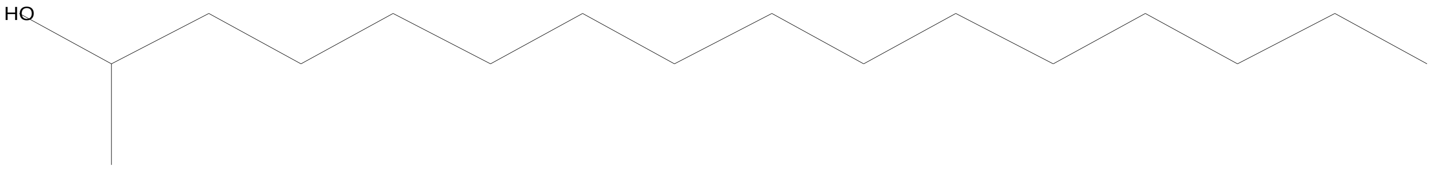

9-Octadecenoic acid, (2-phenyl-1,3-dioxolan-4-yl)methyl ester, cis-
Formula C28H44O4, MW 444, CAS# 56599-45-2, Entry# 40756
(2-Phenyl-1,3-dioxolan-4-yl)methyl 9-octadecenoate, cis-

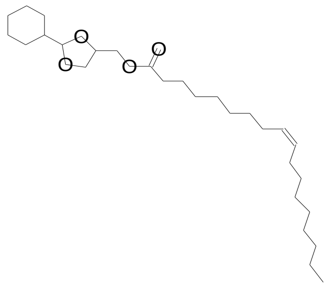

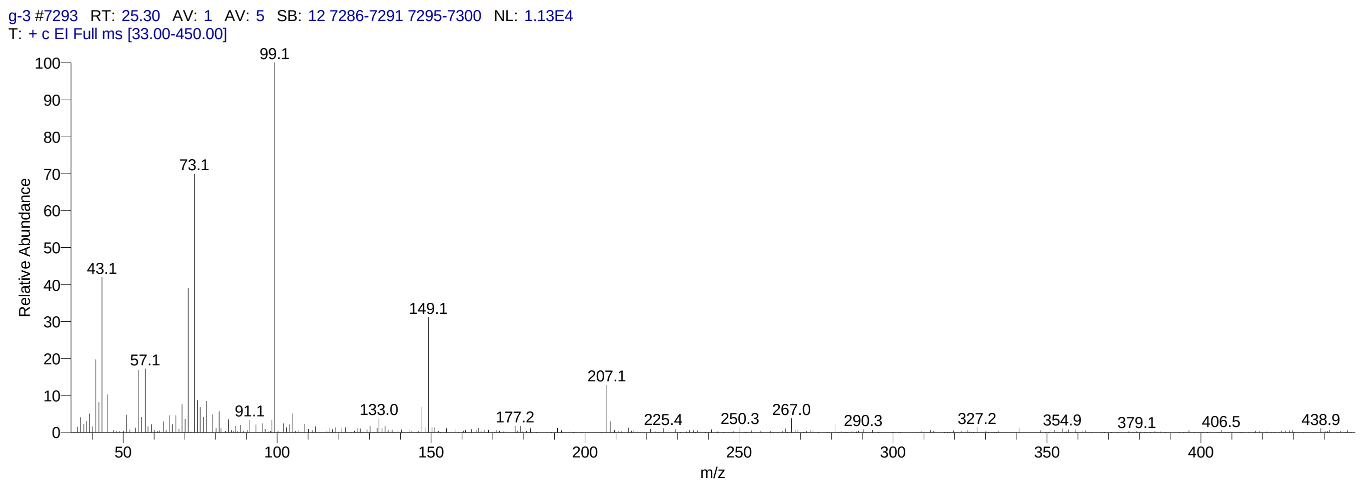

SI	Compound Name	RT	Cas #	Probability
490	Pentanoic acid, 2-methyl-, 1,2,3-propanetriyl ester	25.30	56554-55-3	6.97
481	Hexanoic acid, 3,5-difluorophenyl ester	25.30	NA	5.06
479	14-Oxononadec-10-enoic acid, methyl ester	25.30	NA	4.67

Compound Structure

Pentanoic acid, 2-methyl-, 1,2,3-propanetriyl ester
Formula C21H38O6, MW 386, CAS# 56554-55-3, Entry# 72185
2,3-Bis[(2-methylpentanoyl)oxy]propyl 2-methylpentanoate #

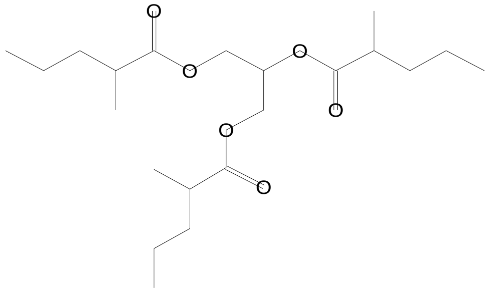

Hexanoic acid, 3,5-difluorophenyl ester
Formula C12H14F2O2, MW 228, CAS# NA, Entry# 10576
3,5-Difluorophenyl hexanoate #

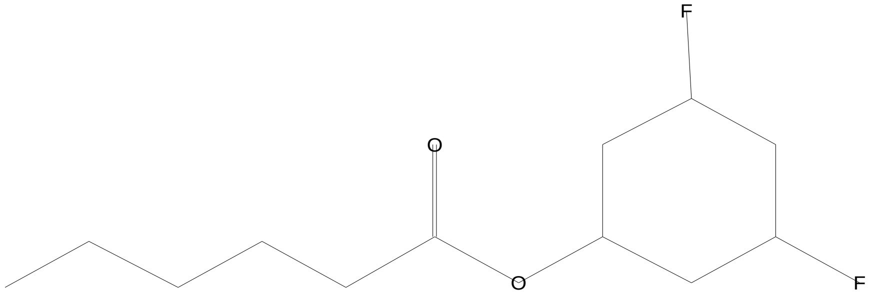

14-Oxononadec-10-enoic acid, methyl ester
Formula C20H36O3, MW 324, CAS# NA, Entry# 72175
Methyl (10E)-14-oxo-10-nonadecenoate #

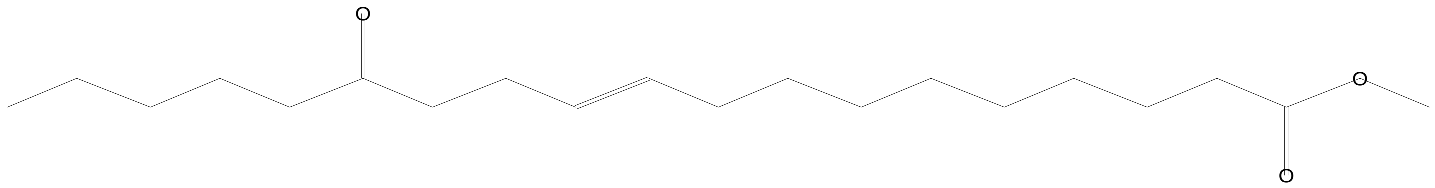

Supplement: DATA SHEET S2 — Report of GC-MS spectra from JZ-GX1. [file Data_Sheet_2.PDF]
